# Monitoring healthcare improvement for mothers and newborns: A quantitative review of WHO/UNICEF/UNFPA standards using Every Mother Every Newborn assessment tools

**DOI:** 10.3389/fped.2022.959482

**Published:** 2022-09-12

**Authors:** Gloria Mutimbwa Siseho, Thubelihle Mathole, Debra Jackson

**Affiliations:** ^1^University of the Western Cape, Cape Town, South Africa; ^2^United Nations Children’s Fund (UNICEF), Windhoek, Namibia; ^3^London School of Hygiene and Tropical Medicine, London, United Kingdom

**Keywords:** assessment tools, monitoring, standards, statements, measures, childbirth, healthcare improvement

## Abstract

**Background:**

Assessment tools with the ability to capture WHO/UNICEF/UNFPA standard quality-of-care measures are needed. This study aimed to assess the ability of Every Mother Every Newborn (EMEN) tools to capture WHO/UNICEF/UNFPA maternal and newborn quality improvement standard indicators.

**Methods:**

A quantitative study using the EMEN quality assessment framework was applied. The six EMEN tools were compared with the WHO/UNICEF/UNFPA maternal and newborn quality improvement standards. Descriptive statistics analysis was carried out with summaries using tables and figures.

**Results:**

Overall, across all EMEN tools, 100% (164 of 164) input, 94% (103 of 110) output, and 97% (76 of 78) outcome measures were assessed. Standard 2 measures, i.e., actionable information systems, were 100% (17 of 17) completely assessed by the management interview, with 72% to 96% of standard 4–6 measures, i.e., client experiences of care, fulfilled by an exit interview tool.

**Conclusion:**

The EMEN tools can reasonably measure WHO/UNICEF/UNFPA quality standards. There was a high capacity of the tools to capture enabling policy environment and experiences of care measures not covered in other available tools which are used to measure the quality of care.

## Introduction

There is an increasing demand to improve quality care as health facility deliveries increase in low- and middle-income countries (LMIC). The time of birth poses the highest risk of dying for newborns, with an occurrence of 2.5 million stillbirths and 2.6 million neonatal deaths occurring in 0–28 days of life annually (1–4). The Sustainable Development Goals for child health will be difficult to meet without a strategic focus to improve quality care around childbirth (1, 2).

Improving the quality of healthcare around the time of birth will reduce maternal and newborn mortality and stillbirths by over half (2), as the provision of high quality of care during childbirth will prevent most intrapartum stillbirths, 61% of neonatal deaths, and half of the maternal deaths (2). The increase in facility deliveries in LMIC may not result in reduced maternal and newborn deaths if the quality of care is also not improved (5, 6).

### WHO/UNICEF/UNFPA quality-of-care standards

In response to the increasing demand to prioritise the quality of care at birth, WHO/UNICEF/UNFPA developed frameworks (Supplementary Figure 1 and Supplementary Table 1) and released standards (Supplementary Table 2) for improving quality care for maternal and newborn healthcare (5, 6). The WHO/UNICEF/UNFPA quality standards include eight standards across two dimensions plus cross-cutting areas of quality The first dimension of quality is ‘provision of care’ with (1) evidence-based care practices, (2) actional information systems, and (3) functional referral systems. The second dimension is ‘experiences of care’ including (4) effective communication, (5) respect and dignified care, and (6) emotional support. The last two are ‘cross-cutting’ and include (7) competent, motivated staff and (8) availability of essential physical resources. The standards are further divided into 31 quality statements. The quality statements are then classified into 352 quality measures. The quality measures are subdivided into 164 input, 110 output/process, and 78 outcome measures. These standards along with the accompanying monitoring framework were published in 2017. While monitoring indicators have been defined for the maternal and newborn period, a specific tool to measure the WHO/UNICEF/UNFPA quality-of-care standards does not exist.

### WHO/UNICEF Every Mother Every Newborn assessment tool: Measuring quality of care

In response to the global initiatives on quality of care, United Nations Children’s Fund (UNICEF) developed nine EMEN quality standards in 2014 (Supplementary Table 1) as a precursor to the 2017 WHO/UNICEF/UNFPA maternal and newborn standards of care. While similar, the 2017 WHO and the initial 2014 UNICEF standards do differ (Supplementary Table 2). First, the EMEN standards include a standard around antenatal care, which is not included in the 2017 standards. Also, in the EMEN tool, there are nine standards that focus on the quality of care during delivery and within 24 h of birth for the mother and newborn (6). The EMEN standards are as follows: (1) Evidence-based safe care is provided during labour and childbirth, (2) evidence-based safe postnatal care is provided for all mothers and newborns, (3) human rights are observed, and the experience of care is dignified and respectful for every woman and newborn, (4) a governance system is in place to support the provision of quality maternal and newborn care, (5) the physical environment of the health facility is safe for providing maternal and newborn care, (6) essential medications, supplies, functional equipment, and diagnostic services are available for maternal and newborn care, (7) qualified and competent staff are available in adequate numbers to provide safe, consistent, and quality maternal and newborn care, (8) health information systems are in place to manage patient clinical records and service data, and (9) services are available to ensure the continuity of care for all pregnant women, mothers, and newborns.

Based on these earlier 2014 standards, the EMEN quality-of-care assessment tools (7) were developed between 2014 and 2016 by harmonising existing global tool(s) at that time. The harmonised tools included WHO Service Availability and Readiness Assessment (SARA), U.S. Agency for International Development (USAID), the Service Provision Assessment (SPA), and EmOC assessment tool for Averting Maternal Death project in Columbia University (AMDD) (8). Jointly, between 2014 and 2016, three countries (7, 8) supported by UNICEF selected relevant questions, pretested them, and organised them by EMEN standards (Supplementary Table 1) into a set of EMEN (unified) tools. The theoretical framework of the EMEN tool (Supplementary Table 3) has strengths in covering all areas of quality care from inputs, outputs/process, and outcomes to the documentation of clientele care provisions (8). The three countries which tested the EMEN tools found the existence of policies, infrastructure, and staff willingness to provide respectful maternity care (8). They also suggested that the EMEN tools assessed facility readiness for implementing the quality-of-care standards for improving maternal and newborn care (8).

### Rationale for the study

Developing strategies to improve quality care during childbirth will be difficult to achieve without the availability of unified quality assessment tools. The EMEN assessment tools exist and have been used to assess and implement quality care in three countries (8–10). However, confirming the ability of the final tool published by UNICEF in 2016 captures the 2017 WHO/UNICEF/UNFPA quality improvement standards for maternal and newborn care is needed. The ability of the EMEN tool to capture quality of care during childbirth is not documented elsewhere. Thus, this study objective is to determine the capacity of 2016 EMEN assessment tools in measuring the 2017 WHO/UNICEF/UNFPA quality improvement standard indicators for maternal and newborn care (Supplementary Table 2). The aim is to document and map the ability of the EMEN tool prior to its administration in the field. The results of this study are further crucial in addressing identified gaps and recommendations from previous studies (11–13).

## Materials and methods

Although the EMEN tool was pretested and used in three UNICEF-supported countries, for this study, pre-assessment and mapping of the capacity of the EMEN tool to gauge the quality of care around childbirth were necessary prior to data collection. The aim was to determine and document the capacity of the EMEN tool/questionnaires in capturing WHO/UNICEF/UNFPA quality standard measures around childbirth before data collection. To document and map the capacity of the EMEN tool/questionnaires, the following steps were applied. Step 1: Two documents were reviewed and gauged against each other: the first document is the WHO standards for improving the quality of maternal and newborn care in health facilities and (5) the second is the UNICEF EMEN facility training manual for assessing the implementation of the standards for improving the quality of maternal and newborn care in health facilities (7). Step 2: A table to match the eight WHO quality standards against the six EMEN tool/questionnaires was created. Each quality standard has two or three quality statements, except for standard 1, which has 13 quality statements, making a total of 31 quality statements. Each of the quality statements comprised six to eight quality measures on the elements of care (inputs, output, and outcome), resulting in 352 quality measures. Each of the six EMEN tool/questionnaires comprises a range of 113 questions (for management and exit interviews) to 197 questions for the medical record review, resulting in a total of 869 questions. Step 3: Each of the 869 questions from all the six EMEN questionnaires against the 352 quality measures within the 31 quality statements under the eight quality standards was populated and matched (Supplementary Appendix A pp. 1–284, Supplementary material). A quality measure was considered a match if one question and/or a wording from any of the EMEN questions matched with a quality measure (Supplementary Appendix A pp. 1–284, Supplementary material). Step 4: A final matrix table (Supplementary Appendix A pp. 1–284) indicating all the questions per EMEN questionnaires (in columns) gauged against each of the WHO standards, statements, and/or measures (in rows) was created. The last page of the Supplementary Appendix A table also shows which of the WHO quality measures were not assessed by any of the six EMEN questionnaires. The matrix table (Supplementary Appendix A pp. 1–284), though large, represents comprehensive summary results of this study by clearly depicting all questions/materials and quality measures assessable by the EMEN tool. To simplify the assessed elements, we further analysed, described, and presented short versions of Supplementary Appendix A 1–284 in Supplementary Figure 1, Supplementary Table 4, and Figures 2, 3.

The mapping of each element and/or question in the EMEN questionnaires to the relevant quality standards, statements, and measures in the WHO/UNICEF/UNFPA framework was carried out by the first author. The second and third authors performed the review of the analysed documents, tables, and figures to ensure alignment or agreement.

### Description of the materials/tools

The EMEN quality assessment tools are designed to narrate a story of care provision through inputs, outputs/process, and outcomes around the time of birth. The tool consists of six structured questionnaires: (1) facility physical, structural, and functional readiness form 1 (F1:PSFR), (2) facility management interview form 2 (F2:MI), (3) facility staff interview with vignettes form 3 (F3:SIV), (4) facility observation of provider–client interactions and care provision form 4 (F4:OPCIC), (5) client medical records review form 5 (F5:CMRR), and (6) women’s exit interview and companion perceptions of care form 6 (F6:WEICPC).

F1:PSFR assesses space, services, equipment, drugs, and supplies used to provide quality care. F2:MI reviews overall facility policies, guidelines, and staff rotation. F3:SIV determines formal and refresher trainings received by staff providing maternal and newborn care and performance of signal functions. F3:SIV also contains vignettes to test staff knowledge and practices. F4:OPCIC follows up a woman presenting in labour as she navigates through the various areas in the facility. F4:OPCIC provides real-time data on care provision and highlights gaps identified. F5:CMRR examines the client’s medical record to capture the quality of data on care provision; assess the quality-of-care content including partograph review, and observe the caesarean section; and also collects data on outcomes. Women’s exit interview (F6. WEICPC) assesses the client at the time of discharge for their perception of quality care provided to them during their hospital stay. F6:WEICPC obtains data from the time of admission, through labour, childbirth, and postnatal care to the time of discharge home. Also, F6:WEICPC addresses examinations, tests, providers’ attitudes, hygiene, and payment of legal and illegal fees.

This study only carried out the pre-assessment and documentation of the capacity of six EMEN tools to capture the WHO/UNICEF/UNFPA quality standards prior to data collection. The actual administration of the EMEN tools and its biases is reported elsewhere.

### Adaptation and familiarisation of the study tools and relevance to other studies

**FIGURE 1:**
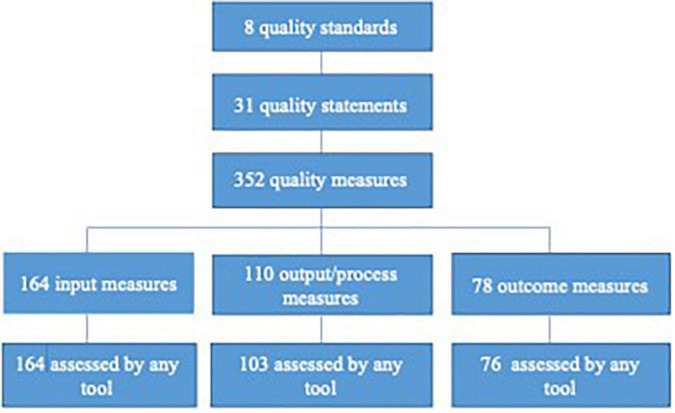
WHO/UNICEF/UNFPA quality of care standards, statements and measures.

The Donabedian and WHO/UNICEF frameworks for facility quality assessment, with three elements of inputs, process/outputs, and outcomes, were applied. Our study was built on other studies that used similar frameworks and tools (8–11, 13). This study used a similar scoring approach as the study by Brizuela et al. (11) who gauged the capacity of a variety of other tools to capture WHO measures. We expanded on these frameworks and created a conceptual model (Figure 1) on how we analysed and gauged the EMEN childbirth assessment tool against the WHO/UNICEF/UNFPA quality standards. Also, Table 1 shows a short version of the WHO/UNICEF/UNPFA and EMEN quality measures that were assessable by using the EMEN tool.

**TABLE 1:** WHO/UNICEF/UNFPA quality standards and EMEN quality measures and tool.

Eight WHO/UNICEF/UNFPA standards, nine EMEN quality measures, by six EMEN tool.		
WHO/UNICEF/UNFPA Standards of care	EQUIVALENT EMEN Quality measures	Assessable EMEN questionnaire per Standards
Standard 1: Every woman and newborn receives routine, evidence-based care and management of complications during labour, childbirth, and the early postnatal period, according to WHO guidelines.	1. Evidence-based safe care is provided during labour and childbirth. 2. Evidence-based safe postnatal care is provided for all mothers and newborns. 5. The physical environment of the health facility is safe for providing maternal and newborn care.	F1.Physical, structural, and functional readiness; F2. Management Interviews; F3. Staff interviews; F4. Observations of provider-client interactions; F5. Medical record review; F6. Women exit interviews.
Standard 2: The health information system enables the use of data to ensure early, appropriate action to improve the care of every woman and newborn.	8. Health information systems are in place to manage patient clinical records and service data	F1.Physical, structural, and functional readiness; F2. Management Interviews; F3. Staff interviews; F4. Observations of provider-client interactions; F5. Medical record review; F6. Women exit interviews.
Standard 3: Every woman and newborn with condition(s) that cannot be dealt with effectively with the available resources is appropriately referred.	9. Services are available to ensure continuity of care for all pregnant women, mothers, and newborns.	F1.Physical, structural, and functional readiness; F2. Management Interviews; F3. Staff interviews; F4. Observations of provider-client interactions; F5. Medical record review; F6. Women exit interviews.
Standard 4: Communication with women and their families is effective and responds to their needs and preferences.	3. Human rights are observed and the experience of care is dignified and respectful for every woman and newborn	F1.Physical, structural, and functional readiness; F2. Management Interviews; F3. Staff interviews; F4. Observations of provider-client interactions; F5. Medical record review; F6. Women exit interviews.
Standard 5: Women and newborns receive care with respect and preservation of their dignity.	3. Human rights are observed and the experience of care is dignified and respectful for every woman and newborn	F1.Physical, structural, and functional readiness; F2. Management Interviews; F3. Staff interviews; F4. Observations of provider-client interactions; F5. Medical record review; F6. Women exit interviews.
Standard 6: Every woman and her family are provided with the emotional support that is sensitive to their needs and strengthens the woman’s capability.	3. Human rights are observed and the experience of care is dignified and respectful for every woman and newborn	F1.Physical, structural, and functional readiness; F2. Management Interviews; F3. Staff interviews; F4. Observations of provider-client interactions; F5. Medical record review; F6. Women exit interviews.
Standard 7: For every woman and newborn, competent, motivated staff are consistently available to provide routine care and manage complications.	7. Qualified and competent staff are available in adequate numbers to provide safe, consistent, and quality maternal and newborn care. 4. A governance system is in place to support the provision of quality maternal and newborn care	F1.Physical, structural, and functional readiness. F2. Management Interviews. F3. Staff Interviews with Vignettes. F4.Observation of Provider-Care Interactions. F5. Medical Record Review. F6. Women Exit Interviews
Standard 8: The health facility has an appropriate physical environment, with adequate water, sanitation and energy supplies, medicines, supplies and equipment for routine maternal and newborn care and management of complications.	5. The physical environment of the health facility is safe for providing maternal and newborn care. 6. Essential drugs, supplies and functional equipment, and diagnostic services are consistently available for maternal and newborn care.	F1.Physical, structural, and functional readiness. F2.Management Interviews. F3.Staff Interviews with Vignettes. F4.Observation of Provider-Care Interactions. F5. Medical Record Review. F6. Women Exit Interviews

### Data analysis

All questions in each assessment tool were considered and matched against the WHO/UNICEF/UNFPA quality improvement standards for maternal and newborn care (5). We conducted descriptive data analysis summaries of all eight quality standards, 31 quality statements, and 352 quality measures. The descriptive analysis was carried out by summarising results into figures and tables. Our analysis included cross-matching questions from the tools with each quality measure within the quality statement under each quality standard (Supplementary Appendix A pp. 1–284).

Our scoring system was similar to that of Brizuela et al. (11). A question/wording from any tool that matched a quality measure was regarded as a match against a WHO quality measure within a particular quality statement (Supplementary material, pp. 1–284). A score of 1 was allocated for a quality measure matching a question. A quality measure was considered a match if one question and wording from the tool fulfilled one of the subcomponents of the quality measure. For instance, quality measure 1.1b:output/process 1.1b.3: “the proportion of all newborns who received all four elements of essential newborn care: immediate and thorough drying, immediate skin to skin contact, delayed cord clamping and initiation of breastfeeding in the first hour” can be matched by a question in the tool asking if the baby was exposed to skin-to-skin contact with the mother immediately after birth (5, 7). In addition, a single question and wording can match with more than one quality measure. For instance, a tool with a question regarding the availability of injectable antibiotics or was the woman/baby given antibiotics can be matched with a quality measure (QM) stating “the health facility has supplies of oral and injectable first-and-second-line antibiotics are available in sufficient quantities at all times for the expected case load” (QM1.7a: input1.7a.1), prophylactic antibiotics (QM1.6a. outcome1.6a.1), and also “appropriate antibiotic therapy” (QM1.8. output/process 1.8.2). Meanwhile, it can also match with the “availability of essential lifesaving medicines in the past three months” (QM8.3 output/process 8.3.1).

### Ethical considerations

No human participants were involved in this study as the focus is on assessing the capacity of the EMEN tools to measure WHO/UNICEF/UNFPA quality improvement standards.

## Results

**FIGURE 2:**
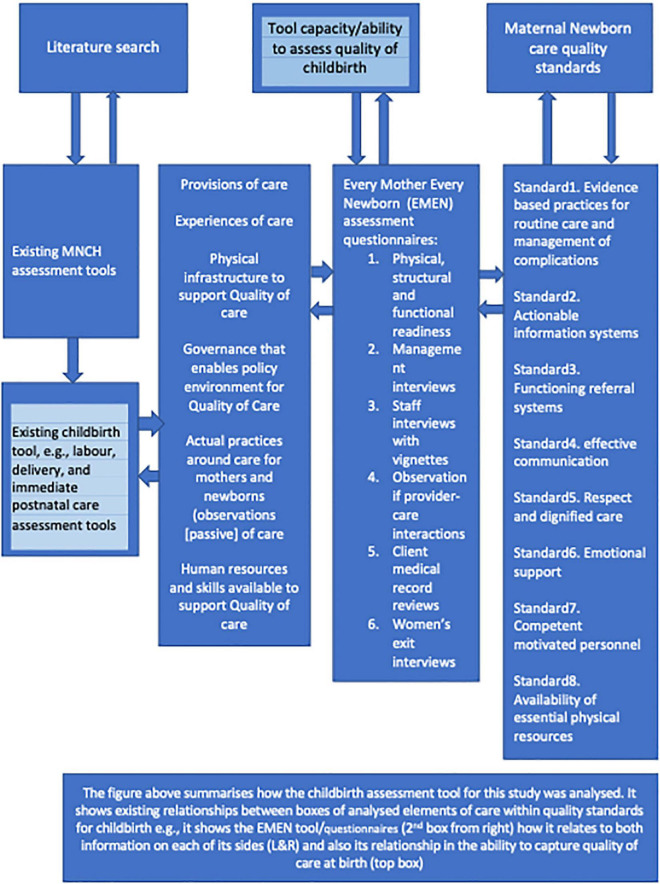
Conceptual model for analysis of childbirth healthcare assessment tools: A comprehensive measure of care elements (inputs, process and output, and outcomes).

**FIGURE 3:**
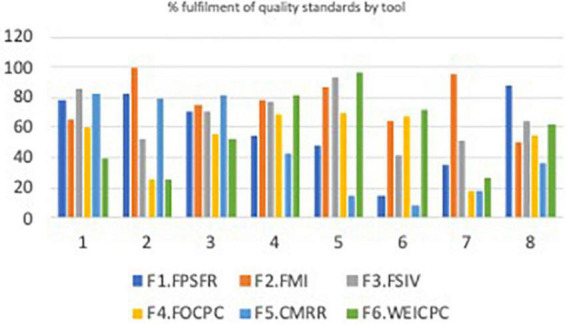
Fulfilment of the quality standards by tool.

The study results outlining the levels of strengths for the six EMEN tool/questionnaires is summarised in various tables and figures. For example, Supplementary Figure 1 presents a summary of assessed WHO/UNICEF/UNFPA quality-of-care standards, statements, and measures. Figure 1 depicts a conceptual model of how the analysis to determine the ability of the EMEN tool in capturing the WHO/UNICEF/UNFPA standards was conducted. Supplementary Table 4 depicts key findings of this study by showing the performance of the EMEN tool against each of the WHO/UNICEF/UNFPA quality standards and statements. Supplementary Table 4 also shows which tool was able to completely, partially, and not assess one or all quality measures. Figures 2, 3 extend the description of results in Supplementary Table 4 to show the fulfilment of the quality standards and statements by the EMEN tool, respectively.

### Summary coverage of WHO/UNICEF/UNFPA standards by the Every Mother Every Newborn tool

All the tools were able to fully assess (e.g., score 100%) two (2) or more quality statements. The management interview (F2:MI) fully assessed eight of the 31 quality statements (1.5, 1.6b, 2.1–2.2, 3.2, 5.1–5.2, and 7.3). The client medical record review (F5:CMRR) fully assessed seven of the 31 quality statements (1.2, 1.3, 1.4, 1.5, 1.7a, 1.7b, and 3.1). The physical structural readiness (F1:PSFR) tool was able to fully assess six of the 31 quality statements (1.2, 1.5, 1.8, 2.2, 8.2, and 8.3). Meanwhile, the staff interview with the vignette form (F3:SIV) fully assessed five of the 31 quality statements (1.6b, 1.7a, 1.7b, 5.1, and 5.2). Our analysis shows that structural, management, staff, and observation tools partially measured all 31 statements by assessing 66, 72, 74, and 55% of the 352 quality measures, respectively. However, the record review and women’s exit interview tools partially measured 29 of the 31 quality statements by capturing 55 and 50% of the quality measures, respectively.

### Completeness of quality measures coverages by Every Mother Every Newborn tool

**FIGURE 4:**
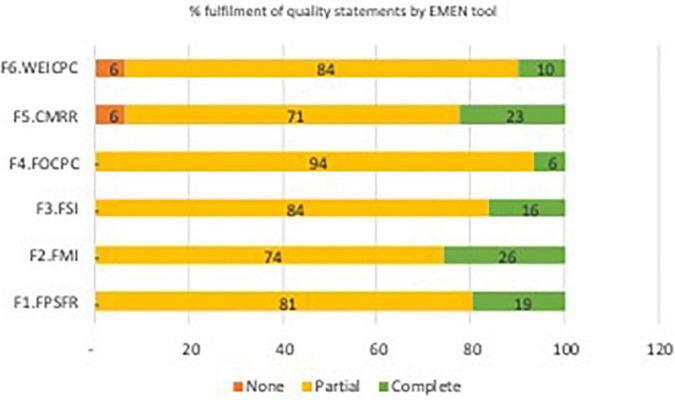
Fulfilment of quality statements by tool.

Figure 3 shows how well each of the tools assessed each of the eight standards. While Figure 4 displays how well each tool was able to assess the 31 quality statements; nine (3%) of the 352 quality measures (Supplementary material, pp. 284) were not assessed by any tool. Further analysis of the quality measures not assessed shows that 56% (five of nine) of the measures were within the experience of the care domain. Of the nine measures, three were related to evidence-based management (standard), two to effective communication, three to emotional support, and one to human resources (standard 7). All the EMEN tools were able to at least partially assess all eight quality standards, although women’s exit interview and record review tools did not assess any measures for quality statements 1.5, 1.7b, 5.2, and 6.1 in standards 1, 5, and 6.

Overall, the tools were most comprehensive in assessing all the three components of care. For example, 100% (164 of 164) of input, 94% (103 of 110) of output/process, and 97% (76 of 78) of outcome measures (Supplementary material, pp. 1–284).

Although, all the tools assessed partially the eight standards. Our analysis shows that structural readiness, management, staff, and record review tools are strong (average 77%) in assessing evidence-based care, actionable information systems, and functioning referral systems (provisions of care). Effective communication, respect and dignity, and emotional support (experience of care domain) measures were highly assessable by management, observation, and exit interview tools at 76% average. At 65% average, management and staff interview tools were strong in capturing human and physical resources, whereas structural readiness captured 88% of physical resources.

## Discussion

This is the first study to assess and compare the capacity of the EMEN childbirth assessment tool to measure WHO/UNICEF/UNFPA quality improvement standards. Thus, our results contribute to providing evidence of the UNICEF EMEN assessment tools for assessing current global standards of quality care at birth. Our results show that the EMEN assessment tools have the capacity to assess across the WHO/UNICEF/UNFPA quality improvement standards for maternal and newborn care at birth. The EMEN tools were able to broadly capture the three provisions of care, three experiences of care, and the two cross-cutting quality standards. This result supports comments from a study (8) on EMEN tools’ strengths in capturing widely maternal–newborn quality improvement standards. In this study, the EMEN tools demonstrated a strong ability by assessing 97% (343 of 352) of the quality measures.

These results show the high capacity of EMEN tools in capturing the eight quality standard measures when compared to 274 measures included in the Brizuela et al. study (11). Our analysed measures were more because we included outcome measures, which were excluded in Brizuela et al. (11). When comparing the proportion of the 274 measures (inputs and outputs) covered in this study to Brizuela et al.’s study (11), we noted that very high percentage of measures were assessable (97.4%) by the EMEN tool. Of the total measures included in our analysis, only 3% measures were not measured by any EMEN tool when compared to 25% of the same measures not assessable in Brizuela et al.’s (12) study, which compared five existing tools separately (Demographic and Health Surveys programme Service Provision Assessment (SPA), the WHO Service Availability and Readiness Assessment, the Averting Maternal Death and Disability programme Needs Assessment of Emergency Obstetric and Newborn Care, and the World Bank’s Service Delivery Indicator (SDI) and Impact Evaluation Toolkit).

Our results suggest that the EMEN tool has a strong ability to assess all three components of care. The completeness of the available measurement was high across the standards. We noted the highest captured measures for standard 2 information systems, standard 3 referral resources, standard 4 effective communication, standard 7 human resources, and standard 8 physical resources. The high completeness for referral systems, physical resources, and standards 3 and 8, respectively, were consistent with the results of Brizuela et al. (11), which focussed primarily on tools that also assess systems and physical resources.

Our analysis also shows the very strong ability of the EMEN tool to capture experiences of the care domain or women’s voices on how they perceived care, whereas previous tools had limited capacity to assess the experience of care within the WHO/UNICEF/UNFPA maternal and newborn quality standards. Capturing women’s voices about their perception of care is crucial for improved healthcare (5, 12). Our analysis shows that women’s exit interviews demonstrated the highest capacity in documenting women’s reports of their experiences of care. Previous studies call to harmonise existing tools to include women’s voices and capture outcome measures (11–13), which are addressed in the EMEN tool as demonstrated by this study. Yet, there is room for improvement as four of the nine measures not assessed by any tool are from experiences of care.

What is also new in this study is that we included an analysis of outcome measures in addition to the inputs and process measures commonly included in other studies (11, 12). The strength of the EMEN tool can be due to the inclusion of detailed input, output/process, and outcome items in their checklists/questionnaires. This is likely due to the EMEN tool being developed by pulling together best interventions of WHO SARA and those used in vigorous research settings (8). It is therefore not surprising that the EMEN tool’s strengths in assessing the eight standards are high and address key weakness from widely available tools (11–13). We however encourage more researchers to implement the EMEN tool and document lessons, strengths, and weaknesses to strengthen the evidence on the ability of the tool.

However, we do note that the EMEN tool is long with pages ranging from 113 (for management and exit interviews) to 197 for the record review. At this stage, we cannot make any recommendations as to whether to shorten and or maintain them. What we can say is that the long and detailed questionnaires cover comprehensively the inputs, outputs, and outcomes and align with most measures across the eight standards. However, there are duplications across tools for some measures, but using any one tool alone does not adequately capture measures across the entire eight standards. There is a need to increase the implementation of the tools to document substantive experience in their usage to inform future recommendations.

### Limitations

Our limitations are similar to the two (2) peer-reviewed published studies (11, 12), which used similar methods. The process of matching or deciding whether a question/item in the tool matched a quality measure within the WHO/UNICEF/UNFPA quality standards can be considered a limitation. Also, we only assessed the EMEN tool which does not assess beyond the childbirth or early newborn period.

## Conclusion

The results of this study can benefit and contribute to future revisions of EMEN assessment tools and WHO/UNICEF/UNFPA quality improvement standards. We call on academia, researchers, programmes, and policymakers to use EMEN tools to assess and determine quality care at birth. More use of the EMEN tools will ensure documentation of gaps, strengths, and opportunities toward maternal and newborn improved birth outcomes.

Although the EMEN tools widely assessed measures across the WHO/UNICEF/UNFPA standards, our analysis clearly shows that six EMEN questionnaires are intertwined and complement each other. Thus, the use of the full suite of tools, instead of single use is recommended, as no single questionnaire is comprehensive enough to capture all the measures.

The EMEN tools have demonstrated good capacity in capturing or determining quality healthcare provided at birth. As determining the quality of care at birth is crucial in informing strategic interventions at birth toward improved birth outcomes for women and newborns. Our results noted that other existing tools tended to emphasise input measures and were inadequate in assessing the experience of care. The need for consensus and harmonised key indicators from the WHO/UNICEF/UNFPA standards into a unified tool cannot be overstated.

## Data availability statement

The original contributions presented in this study are included in the article/Supplementary material, further inquiries can be directed to the corresponding author.

## Ethics statement

The Ethical Review Board of the national-level Ministry of Health and Social Services (MoHSS), Namibia (Ref: 17/3/3) and the Research Ethics Committee of the University of the Western Cape (Ref: BM17/10/4) gave approval to carry out the study in Namibia. This study did not involve human participants.

## Author contributions

GS led data collection and data analysis, and linkage with ministry and local stakeholders. TM and DJ were co-investigators and supervisors for the research. All authors participated in the conceptualisation of the study and approved the final manuscript.
