# Supplementary material for: Monitoring healthcare improvement for mothers and newborns: A quantitative review of WHO/UNICEF/UNFPA standards using Every Mother Every Newborn assessment tools
Source: Front Pediatr. 2022 Sep 12;10:959482. doi: 10.3389/fped.2022.959482 (PMC9510702; doi:10.3389/fped.2022.959482)
Supplement: Supplementary file 1 [file Data_Sheet_3.PDF]

Appendix A. Operationalization of each of the WHO/UNICEF/UNFPA standards according to items measured through the forms on physical structure (F1.PSFR), management interview (F2.MI), staff interview and vignette (F3.SIV), observations (F4.OPCI), records (F5.CMRR) and women exit interviews (F6.WEIPC)

**WHO quality measures**	**Tools**					
**Standar1: Every woman and newborn receives routine, evidence-based care and management of complications during labour, childbirth and the early postnatal period, according to WHO guidelines.**						
**Standard1.1a**						
**Quality statement 1.1a: Women are assessed routinely on admission and during labour and childbirth and are given timely, appropriate care**						
Input measures	F1.PSFR	F2.MI	F3.SIV	F4.OPCI	F5.CMRR	F6.WEICPC
1.The facility has the basic essential equipment and supplies for routine care and detection of complications (thermometers, sphygmomano meter, fetal stethoscopes, urine dipsticks) available in sufficient quantities at all times in the	1.Does the facility has the following and functioning: BP apparatus/sphyg momanometer, stethoscope, thermometer-digital/mercury column, uristix/dipstix, glucometer, monitor (pulse oximeter), ultrasound scan machine, wall	Does this facility have Sphygmomano meters?	Does this facility provide Weight/tempera ture as newborn signal functions and were they done in the past 6 months?<br><br>Vignettes: Measure woman's blood pressure?<br><br>Check her urine for protein?	1.Abdominal palpation for uterine contractions<br><br>2. Listened to the fetal heart beats<br><br>3. Blood pressure taken<br><br>4. Urine test done	1. How many times was the woman's temperature checked and recorded between first exam and delivery?<br><br>2. How many times was the blood pressure checked and recorded between first	1.Check your BP<br><br>2. Take your urine and checked<br><br>3. Examined your abdomen<br><br>4. Listened to your baby's fetal heart beats

areas of the maternity unit for labour and childbirth.	clock with second hand, fetoscope/sonic aid, trolley, stretcher, examination table,		Check fetal heart rate?		exam and delivery?<br><br>3. Was the fetal heart rate measured before Caesarean Section?	
2. The health facility has written, up to date clinical protocols for assessing intrapartum care and action in the labour and childbirth areas of the maternity unit that are consistent with WHO guidelines.		1.What are the main policies of this facility regarding maternity and newborn care? Collect any point	Is there an IPC policy available in your facility?			
3.The health care staff in the labour and childbirth areas		1. Are there additional refresher	1.Have you receive refresher/in-service training		1. What was done for the woman who suffered from	Did you have any of the following

of the maternity unit receive in-service training and regular refresher sessions at least once every 12 moths in the identification and management of obstetric emergencies during labour and childbirth		training for the staff?<br>3. Have there been training over the past year?<br>4. How often have they been trained over the past year?<br>5. What do the following training sessions cover? (Prompt if not mentioned): Management of pregnancy complications, management of birth complications, triage, essential newborn care.	in the past months on the following: management of obstetric triage/initial assessment?; Haemorrhage (ante-and postpartum?; Management of puerperal sepsis?; management of pre-eclampsia and/ or eclampsia? ; management of prolonged or obstructed labour?		pregnancy complication?<br><br>2. what was done for the woman with an obstetric complication that occurred during labour/delivery/ immediate postpartum?<br><br>3. if live birth with complication (whether dead or alive) what was done for the baby(ies) with the complications?	complications after the birth?<br>a)excessive bleeding that wet your clothes, poured on the floor, needed transfusion with blood or needed operation again?<br><br>b) Collapsed or convulsed?<br><br>c) you were told your BP was high<br><br>d) you had a temperature or fever
4.Health care staff in labour and childbirth areas receive at		1. Are there additional refresher training for the	1.Have you receive refresher/in-service training		1. What was done for the woman who suffered from	Did you have any of the following

least monthly drills or simulation exercises and supportive supervision in routine care and detection of obstetric complications during labour and childbirth.		staff in maternity and paediatric ward?<br><br>3. How often have they been trained over the past year?<br><br>4. What do the following training sessions cover? (Prompt if not mentioned): Management of pregnancy complications, management of birth complications, triage, quality of care, newborn resuscitation, essential newborn care?	in the past months? Yes/no (in management of obstetric triage/initial assessment, obstetric haemorrhage (ante-and postpartum/management of puerperal sepsis/management of pre-eclampsia and/ or eclampsia, management of prolonged or obstructed labour, management of third stage of labour, labour monitoring, partograph use.		pregnancy complication?<br><br>2. what was done for the woman with an obstetric complication that occurred during labour/delivery/ immediate postpartum?<br><br>3. if live birth with complication (whether dead or alive) what was done for the baby(ies) with the complications?	complications after the birth? a)excessive bleeding that wet your clothes, poured on the floor, needed transfusion with blood or needed operation again?<br><br>b) Collapsed or convulsed?<br><br>c) you were told your BP was high<br><br>d) you had a temperature or fever

Output/process measures	F1.PSFR	F2.MI	F3.SIV	F4.OPCI	F5.CMRR	F6.WEICPC
1.The proportion of all women who gave birth in the health facility whose blood pressure, pulse, and temperature were appropriately recorded during labour, childbirth and the early postnatal period (and acted on if appropriate)	Does the facility have any of the following and are they functioning?<br>-monitor (pulse oximeter), BP apparatus/Sphygmomanometer, Stethoscope, Thermometer-digital/mercury column	Is there a form of external calibration of equipment used in this facility such as standard boards/authority?<br><br>Sphygmomanometers?	Does this facility provide Weight/temperature as newborn signal functions and were they done in the past 6 months?<br><br>Vignettes:<br>1.Measure woman's blood pressure<br><br>2. Check the woman`s vital signs<br><br>3. Take the temperature with a thermometer	1.Was the history taken?<br><br>2. Was blood pressure taken?<br><br>3. Baby's temperature taken after birth?	1.How many times was the woman's temperature checked and recorded between first exam and delivery?<br>2. How many times was blood pressure checked and recorded between first exam and delivery?<br>3. How many times was the mother's pulse checked and recorded between first exam and delivery?	1.Did they check your BP?<br><br>2. you were told your BP was high<br><br>3. you had a temperature or fever

2.The proportion of all women who gave birth in the health facility who received oxytocin within 1 min of the birth of their baby.	Does the facility provide Oxytocic (syntometrine/Ergot) as an essential drug?		Was Injectable oxytocic (synto/Ergo) drugs administration provided in this facility as a signal EmOC function in the past 6 months?	1.Was oxytocin administered?	1.Was the woman given parenteral oxytocin?	Were you given any of IV fluid/medicine to take/Injection(IM)/pain relief during labour?
3.The proportion of all women who gave birth in the health facility whose progress in labour was correctly monitored and documented with a partograph and a 4-h action line.			Have you received refresher/in-service training in the past 12 months? If yes, on partograph use?	Was the woman started on the partograph?	1.Was partograph used to monitor labour?<br><br>2. Was the woman's partograph opened?<br><br>3. Was the women's dilatation on the alert line of partograph when it started?	

4.The proportion of all women who gave birth in the health facility whose urinalysis result was appropriately recorded during labour, childbirth and the early postpartum period (and acted on if appropriate).	Does the facility provide the Uristix/dipstix service?		Vignette: Check her urine for protein	Was urine test done?	What was done for the woman with a complication?	Did they take your urine and checked?
5.The proportion of all women who gave birth in the health facility who received any option for pain relief during labour and childbirth.	Does the facility have essential drugs? (IV antibiotics/Magnesium Sulphate/IV Diazepam/Ampicillin/Penicillin for IV/Gentamicin for IV…)					Were you given pain relief during labour?

Outcome measures	F1.PSFR	F2.MI	F3.SIV	F4.OPCI	F5.CMRR	F6.WEICPC
1.The health facility perinatal mortality rate [number of foetal death (stillbirths) or early neonatal deaths / the total number of births of babies weighing at least 1000 g or of 28 weeks' gestation (stillbirths + live births) x 1000]	1.Does the facility routinely conduct audit for early neonatal deaths?<br><br>2. Does this facility routinely conduct audit for stillbirths	Does your facility have a quality improvement programme which includes i) quality improvement teams; iii) quality improvement mentoring/coaching; iv) capturing and use of data; v) monitoring and evaluation	Is weight provided in this facility as a signal newborn function and was it provided in the last 6 months?	What was the weight for the baby?<br><br>Newborn not breathing spontaneously at birth?	1. If stillbirth/early neonatal death, what was the primary underlying cause of death?<br><br>2. If yes what was the birth weight of baby1/baby2/baby3?<br><br>3. Number of completed weeks of gestation at delivery?	
2.Intrapartum stillbirth rate (number of stillbirths occurring during the intrapartum	1.Does the facility routinely conduct audit for early	Does your facility have a quality improvement programme which includes i) quality	Is weight provided in this facility as a signal newborn function and was it provided	What was the weight for the baby?<br><br>Newborn not breathing	1. If stillbirth/early neonatal death, what was the primary underlying cause of death?	

period per 1000 births).	neonatal deaths?<br><br>2. Does this facility routinely conduct audit for stillbirths	improvement teams; ii) quality improvement plans; iii) quality improvement mentoring/coac hing; iv) capturing and use of data; v) monitoring and evaluation	in the last 6 months?	spontaneously at birth?	2. If yes what was the birth weight of baby1/baby2/ba by3?<br><br>3. Number of completed weeks of gestation at delivery?	
3.The proportion of all women admitted to the health facility in active labour who gave birth within 12 h.				1.Time of arrival of woman<br><br>2. Record delivery time<br><br>3. Birth attendant notes/shouts time of delivery	1.Date of presentation at the health facility?<br><br>2. Time of presentation at the health facility?<br><br>3. Date and time of delivery?	
Quality statement 1.1b						
Newborns receive routine care immediately after birth						

Input measures	F1.PSFR	F2.MI	F3.SIV	F4.OPCI	F5.CMRR	F6.WEICPC
1.The health facility has written, up-to-date, clinical protocols for essential newborn care that are consistent with WHO guidelines and are available in the labour and childbirth areas of the maternity unit.		1. The facility has specific management policies on the following:<br>i)Baby friendly<br>ii) Early Initiation of breastfeeding<br>iii)Rooming in<br>iv)KMC for babies<br>v) family/partner involvement in in maternity and newborn care	1.Recorded evidence of KMC (prolonged STSC, EBF/feeding support, growth monitoring (weight)<br><br>2. Is there an IPC policy available in your facility?			
2.The health facility has supplies of sterile cord ties (or clamps) and scissors (or blades), available in sufficient	Does the facility provide services including?<br>i) cord clamps?<br><br>ii) delivery forceps		1. Does this facility provide delayed cord cutting as a newborn signal function and was it done in the past 6 months?	List contents of the delivery tray (cord clamp/sharp scissors/sutures and needle).		

quantities at all times for the expected number of births			2. Does this facility provide cord care cutting as a newborn signal function and was it done in the past 6 months?			
3.The health facility has supplies of clean towels in the labour and childbirth areas for immediate drying of newborns, available in sufficient quantities at all times for the expected number of births	Does the facility have functioning radiant warmers?		Vignette:<br>1.Ensure extra warmth for the baby<br><br>2. Give SSC/KMC by mother put in incubator for rewarming<br><br>3. check if windows are open and close them all as well as any fans on the ward to prevent draught	1.List the items in the resuscitation area)?<br>i)Clean towels – at least 2<br>ii) radiant warmer<br><br>2. Baby dried immediately and thoroughly<br><br>3. list the contents of the delivery tray (clean towels x 2)w1		

4.Health-care staff in the labour and childbirth areas of the maternity unit receive in-service training or regular refresher sessions in essential newborn care and breastfeeding support at least once every 12 months		1. Are there additional refresher training for the staff?<br>3.How often have they been trained over the past year?<br><br>4.What do the following training sessions cover? (Prompt if not mentioned): quality of care, newborn resuscitation, essential newborn care.	1.Have you receive refresher/in-service training in the past months?<br>2.Yes/no . If yes, was it in breastfeeding counselling and lactation management?		Was the woman counselled on newborn danger signs, breastfeeding?	1. Are you satisfied with the information you received on breastfeeding?<br><br>2. Can you mention/give some of the danger signs in newborn? (having difficult in breathing/has chest indrawing/convulsions/fever or too hot to touch/feels very cold to touch/unable to feed or stopped feeding/develops yellow skin/others, specify?
5.The health facility has local	Does the facility have		1. Was temperature	1. Baby's temperature	How many times was the	

arrangements and a mechanism to maintain a documented room temperature in the labour and childbirth areas at or above 25 °C and free of draughts	Thermometer- digital/mercury column?		provided in this facility as part newborn signal function and was it provided in the past 6 months?<br><br>Vignette:<br>2. take the temperature with a thermometer<br><br>3. check if windows are open and close them all as well as any fans on the ward to prevent draught	taken after birth?<br><br>2. Baby's weight	woman's temperature checked and recorded between the first and last exam?	
6.Health-care staff in the labour and childbirth areas receive at least monthly drills or simulation	1. Graduated cup to measure expressed breast milk.<br><br>2. Small cup for feeding	1. What do the training sessions cover? : quality of care, newborn resuscitation,	1.Have you receive refresher/in-service training in the past 12 months? Yes/No	1.Baby dried immediately and thoroughly.<br><br>2. Cord clamped and cut.	Was the woman counselled on breastfeeding?	1.For how long after birth did you first put your baby (ies) to the breast

exercises and supportive supervision in essential newborn care and supporting breastfeeding.	expressed breast milk.	essential newborn care.<br><br>5. How does the facility monitor staff performance?<br><br>6. Do you receive external supervisory visits to this facility?	2. If yes was it in breastfeeding counselling and lactation management?<br><br>3. Early Essential Newborn Care<br><br>4. Essential Care during labour and childbirth	3. Baby placed in skin to skin contact with the mother immediately after birth.<br><br>4. Time baby was first put to the breast.		2. Was the newborn put skin to skin with the mother immediately after birth?
Output/process measures	F1.PSFR	F2.MI	F3.SIV	F4.OPCI	F5.CMRR	F6.WEICPC
1.The proportion of all newborns who were breastfeed within 1 h of birth	1. Graduated cup to measure expressed breast milk.<br><br>2. Small cup for feeding expressed breast milk.	1. Facility specific management policy on early initiation of breastfeeding<br><br>2. Facility specific management policy on	Was Early initiation and exclusive breastfeeding provided in this facility as newborn signal functions and was it done in the past 6 months.	Time baby was first put to breast?	How long after birth was the baby put to the mother's breast?	For how long after birth did you first put your baby(ies) to the breast?

		exclusive breastfeeding or breastmilk feeding<br><br>3. Facility specific management policy on Baby friendly				
2.The proportion of all newborns who were kept skins to skin contact (with body and head covered) with their mothers at least 1 h after birth	Facility specific management policy on Baby friendly	Does the training sessions for health workers in maternity and children's ward cover i) Essential newborn care?	Is skin to skin contact provided in this facility as part of newborn signal function, and was it done in the last 6 months?	1.Baby placed skin to skin contact with the mother? immediately after birth.<br><br>2.Baby delivered into mother's abdomen?<br><br>3.When was the baby taken out of skin to skin contact with the mother?		Was the newborn put skin to skin with the mother immediately after birth?

3.The proportion of all newborns who received all four elements of essential newborn care: immediate and thorough drying, immediate skin-to-skin contact, delayed cord clamping and initiation of breastfeeding in the first hour.	The facility has functional Cord clamps?	1.Does the training sessions for health workers in maternity and children's ward cover i) Essential newborn care?<br><br>2. facility specific management policies include: i) Early Initiation of breastfeeding? ii) Exclusive breastfeeding or breastmilk feeding iii)Rooming in	Is essential newborn care, drying thoroughly, skin-to-skin contact, delayed cord cutting, and Early initiation of exclusive breastfeeding newborn signal functions provided in this facility and were they done in the past 6 months?	1.Baby dried immediately and thoroughly.<br>2.Baby placed skin to skin contact with the mother immediately after birth.<br>3.Cord clamped and cut.<br>4.Time baby was first put to breast?	How long after birth was the baby first put to the mother's breast?	1.For how long after birth did you first put your baby(ies) to the breast?<br>2.Was the newborn put skin to skin with the mother immediately after birth?
4.The proportion of all newborns whose umbilical cord was clamped 1-3 min after birth	The facility has functional Cord clamps?	Does the training sessions for health workers in maternity and children's	Delayed cord cutting a newborn signal function provided in this facility and was	Cord clamped and cut.		

		ward cover i) Essential newborn care?	it done in the past 6 months?			
5. The proportion of all newborn who were dried immediately and thoroughly at birth.		Does the training sessions for health workers in maternity and children's ward cover Essential newborn care?	Is Drying thoroughly a newborn signal function provided in this facility and was it done in the past 6 months?	Baby dried immediately and thoroughly.		
Outcome measure	F1.PSFR	F2.MI	F3.SIV	F4.OPCI	F5.CMRR	F6.WEICPC
The proportion of newborns who had a normal body temperature (36.5-37.5 0c) at the first complete examination (60-120 min after birth).	Does the facility have functional Thermometers-digital/mercury column?		1.Is temperature checking/monitoring provided as a newborn signal function in this facility and was it done in the past 6 months?<br><br>Vignette:<br>2. Feel if baby is too cold? Take	1.Was the baby temperature taken after birth?<br>2.If yes, what was the baby temperature?		

			the temperature with a thermometer.			
Quality statement 1.1c						
Newborns receive routine postnatal care						
Input/process measure	F1.PSFR	F2.MI	F3.SIV	F4.OPCI	F5.CMRR	F6.WEICPC
1.The health facility has written, up-to-date clinical protocols for postnatal care in the maternity and/ or postnatal care areas of the maternity unit that are consistent with WHO guidelines	Is there a protocol for infection control	1.What are the main policies of this facility regarding maternity and newborn care? Collect any point.<br><br>2.Does the facility has specific management policies on Baby friendly?<br><br>3. Policy on rooming in?	1.Is there IPC policy available in your facility?<br><br>2. do you routinely follow up mothers and newborns after discharge from the facility?			Are you satisfied with the information you received about Postpartum care and hygiene?

2.The health facility practises and enables rooming-in to allow mothers and babies to remain together 24 h a day		1. Health facility specific management policy on Baby friendly<br><br>2.Facility specific management policy on rooming in.				
3.The health facility has a written breastfeeding policy that is routinely communicated to all health care and support staff	1. Graduated cup to measure expressed breastmilk<br><br>2. Small cup for feeding expressed breastmilk	1.What are the main policies of this facility regarding maternity and newborn care? Collect any point.<br>2.Does the facility has specific management policies on<br>i)Baby friendly;<br>ii) early initiation of breastfeeding;	1.Have you received refresher/in-service training in the past 12 months on<br>i)Breastfeeding counselling and lactation management?<br>ii) Early Essential newborn care<br><br>2. Vignette:<br>i) Ensure breastfeeding is	Was the woman counselled on breastfeeding?	Was the woman counselled on breastfeeding?	1. Are you satisfied with the information n you received about breastfeeding?<br><br>2. Do you feel competent enough to be able to breastfeed your baby on your own?

		iii)exclusive breastfeeding or breastmilk feeding?	established and provide support if necessary;<br><br>ii)watch her breastfeed her baby and teach her good positioning and attachment;<br><br>iii) if baby not breastfeeding, teach her to express the milk and feed with a clean cup;<br><br>iv) Encourage infant formula only if EBM is not possible and mother can afford<br><br>v) Educate her and encourage her to practice			

			exclusive breastfeeding for the 1st 6 months of the baby's life			
4.Health-care staff in the maternity unit receive in-service training and regular refresher sessions in routine postnatal care and breastfeeding at least every 12 months	Graduated cup to measure expressed breastmilk<br><br>2. Small cup for feeding expressed breastmilk	1.What additional training is provided to workers posted there?<br>2.Are there additional refresher training for the staff?<br>3.Have there been training over the past year?<br>4.How often have they been trained over the past year?	1.Have you received refresher/in-service training in the past months? If yes, was it in breastfeeding counselling and lactation management?<br>2.management of third stage of labour?<br>3.Management of obstetric complication (postpartum)	Was the woman counselled on breastfeeding?	Was the woman counselled on breastfeeding?	1.Are you satisfied with the information n you received about breastfeeding?<br><br>2. Do you feel competent enough to be able to breastfeed your baby on your own?
5.The health facility has local arrangements to ensure that		1.The facility management policies on Pre-discharge	1.Do you routinely follow up mothers and newborns after	Mother counselled on postnatal care.	1.Was the woman counselled on breastfeeding?	1.Are you satisfied with the information you received

every mother knows when and where postnatal care for herself and her newborn will be provided after discharge from the hospital.		evaluation of mother and baby;<br><br>2. The facility management policies on Pre-discharge counselling for mother and baby?<br><br>3. do you have a system to make postnatal home visits?	discharge from the facility?<br><br>2. How many days of the week do you offer PNC services?<br><br>3. is postpartum family planning services available?		2.Was the woman counselled on care seeking for illness?<br>3.Was the woman counselled on postpartum care?<br>4.Was the woman counselled on immunization?	about the postpartum care and hygiene?
6. The health facility has local arrangements for alternative feeding methods, including cup or cup-and spoon feeding, and avoids bottle-feeding	1.Does the facility have functional graduated cup to measure expressed breast milk?<br><br>2. Does the facility have functional small cup for	1. Health facility specific management policy on baby friendly<br><br>2. Health facility specific management policy on Early Initiation of breastfeeding	If baby not breast feeding teach the mother to express the milk and feed with a clean cup.	Was the woman counselled on breastfeeding?	Was the woman counselled on breastfeeding?	1.Are you satisfied with the informatio n you received about breastfeeding?<br><br>2. Do you feel competent enough to be able to breastfeed your

	feeding expressed breast milk?	2. Health facility specific management policy on excusive breastfeeding or breastmilk feeding				baby on your own?
7.The health facility has local arrangement to inform pregnant women and their families about the benefits and management of breastfeeding	1.Does the facility have functional graduated cup to measure expressed breast milk?<br><br>2. Does the facility have functional small cup for feeding expressed breast milk?	1.Does the health facility has policy on Early initiation of breastfeeding?<br><br>2.Does the health facility has policy on exclusive breastfeeding or breastmilk feeding?<br><br>3. specific management policy on partner/family	1.Do you offer ANC care to pregnant women in this facility?<br><br>2. have you received refresher/in-service training in the past 12 months on breastfeeding counselling and lactation management?	Was the woman counselled on breastfeeding?	Was the woman counselled on breastfeeding?	1.Are you satisfied with the information you received about breastfeeding?<br><br>2.Do you feel competent enough to be able to breastfeed your baby on your own?

		involvement in maternity and newborn care.				
8.The health facility ensures that feeding of infant formula is demonstrated to mothers and family members of newborns only when needed, with a full explanation of the hazards of improper use.	1. Facility has functional Graduated cup to measure expressed breastmilk.<br><br>2. facility has functional small cup for feeding expressed breast milk.	1.Health facility management policy on Early initiation of breastfeeding?<br><br>2. Health facility management policy on exclusive breastfeeding or breastmilk feeding?<br><br>3. Health facility management policy on baby friendly	Vignette: Encourage infant formula only if EBM is not possible and mother can afford.	Time baby was first put to the breast?	Was the woman counselled on breastfeeding?	1. Are you satisfied with the information you received about breastfeeding?<br><br>2.Do you feel competent enough to be able to breastfeed your baby on your own?
Output/process measures	F1.PSFR	F2.MI	F3.SIV	F4.OPCI	F5.CMRR	F6.WEICPC
1.The proportion of all newborns on postnatal care			1.Is Vitamin K provided in this facility as part of newborn	Newborns immunized for Hep B0, BCG, bOPV.	Was the woman counselled on immunization?	

wards or areas in the health facility who received vitamin K and full vaccination as per national guidelines			signal function, and was it done in the last 6 months?<br><br>2. Does this facility offer immunization (EPI vaccines) to children?			
2.The proportion of all stable newborns in the health facility who are fed exclusively on breast milk from birth to discharge	1. Facility has functional Graduated cup to measure expressed breastmilk.<br><br>2. facility has functional small cup for feeding expressed breast milk.	Facility specific management policies on i) Baby friendly; ii) Early Initiation of breastfeeding; iii) Exclusive breastfeeding or breastmilk feeding	1.Is Early Initiation and Exclusive breastfeeding provided and done in this facility as part of newborn signal function, in past 6 months?<br>Vignette:<br>2. Educate her and encourage her to practice exclusive breastfeeding for the 1st 6	Was the woman counselled on breastfeeding?	Was the woman counselled on breastfeeding?	1.Are you satisfied with the information you received about breastfeeding?<br>2. Do you are competent enough to be able to breastfeed your baby on your own?

			months of the baby's life			
3.The proportion of all women in postnatal care wards or areas in the health facility who have documented problems of blood pressure, pulse rate, vaginal bleeding, lochia or breastfeeding	Bp apparatus/sphygmomanometer<br><br>Stethoscope<br><br>Monitor (pulse oximeter)	Is there a form of external calibration of equipment used in this facility such as standards boards/authority? Sphygmomanometers	Vignette:<br>1.Measure woman's blood pressure<br><br>2. If baby not breastfeeding, teach her to express the milk and feed with a clean cup.	1.Was there any complications in the labour and delivery?<br>2. What complications? List (tears, rupture, PPH, obstruction, etc)	1.Which of the following obstetric problem occurred during labour and/ or delivery and/or immediately postpartum?<br><br>2.Pre-eclampsia/eclampsia<br><br>3.Postpartum haemorrhage.<br>4.Third degree tear	1.Check your BP?<br>2.Check whether you were bleeding?<br><br>3. examined your abdomen?
4.The proportion of all newborns in the health facility who received a full clinical examination before discharge	Does the facility have a functioning examination table?	1.Does the facility have a specific management policy on pre-discharge evaluation of	1.Have you received formal training in the care or routine care of newborns?	Was the baby examined from head to toe before discharge?	Was/were the baby(ies) thoroughly examined any time after birth before discharge?	1.Did the health worker examine your baby after birth?<br><br>2. Was the baby examined from head to toe

		mother and baby?				before discharge?
5.The proportion of all healthy mothers and newborns who received care for at least 24 h after an uncomplicated vaginal birth in a health facility	1.Does this facility admit patients over night?<br><br>2. Does this facility have a separate maternity ward?	Are maternity and children's wards separately run?	1.Does this facility offer 24hr services?<br><br>2. Are delivery services available 24hrs in a day?	1. Time of arrival of woman<br><br>2. Day of the week<br><br>3. Time of first professional health worker contact<br><br>4. Record time of delivery	1.How long was the woman hospitalized before discharge? (days/hours).<br><br>2.Date and Time of delivery<br><br>4.Date of discharge.<br><br>5.Time of discharge	If you had your way, how would you have preferred your length of stay after delivery?
6.The proportion of all newborns on postnatal care wards or areas in the health facility for whom there is documented information on	Does the facility have functioning monitor (pulse oximeter), thermometer-digital/mercury column?		Vignettes:<br>1.Check fetal heart rate<br><br>2. Take the temperature with a thermometer	1.Baby's temperature taken after birth?<br>2.What was baby temperature?	1.Was the fetal heart rate recorded?<br>2.What was the highest FHR?<br>3.What was the lowest FHR?<br>4.What was the last FHR measured before	

the newborn body temperature, respiratory rate, feeding behaviour and the absence or presence of danger signs			3. Is temperature provided in this facility as a newborn signal function and was it done in the past 6 months.		the Caesarean Section?<br>4.If live birth with complications (whether dead or alive) what complication did the baby(ies) have?	
7.The proportion of all healthy mothers on postnatal wards or areas in the health facility who received breastfeeding counselling and support from a skilled health care provider	1. Facility has functional Graduated cup to measure expressed breastmilk.<br><br>2. facility has functional small cup for feeding expressed breast milk.	Facility has specific policies i)Baby friendly ii) Early Initiation of breastfeeding iii) Exclusive breastfeeding or breastmilk feeding	1.Is Early Initiation and exclusive breastfeeding provided as a newborn signal function in this facility and was it done in the past 6 months?<br><br>2. Have you received refresher/in-service training in the past 12 months on	Was the woman counselled on breastfeeding?	Was the woman counselled on breastfeeding?	1. Are you satisfied with the information you received on breastfeeding?<br><br>2.Do you feel you are competent enough to be able to breastfeed your baby on your own?

			breastfeeding counselling and lactation management?<br><br>3. Educate her and encourage her to practice exclusive breastfeeding for the 1st 6 months of the baby's life.			
8. The proportion of all women who gave birth in the health facility who were allowed to room-in with their newborn 24 h a day		Facility specific management policy on rooming?<br><br>Facility specific management policy on baby friendly?				
9. The proportion of all postpartum women in the health facility		Pre-discharge counselling for the mother and baby	1.Does the facility offer contraceptive services?	Was the woman counselled on family planning?	Was the woman counselled on contraception?	Are you satisfied with the information you received on

who were offered counselling on birth spacing and family planning methods before discharge			2. Is postpartum family planning services available?			family planning?
Outcome measures	F1.PSFR	F2.MI	F3.SIV	F4.OPCI	F5.CMRR	F6.WEICPC
1.The proportion of all newborns in the health facility who were exclusively breastfed at the time of discharge from hospital	1. Facility has functional Graduated cup to measure expressed breastmilk.<br><br>2. facility has functional small cup for feeding expressed breast milk.	Facility specific management policies on i) Baby friendly; ii) Early Initiation of breastfeeding; iii) Exclusive breastfeeding or breastmilk feeding	1.Is Early Initiation and Exclusive breastfeeding provided and done in past 6 months as part of newborn signal function?<br><br>Vignette:<br>2. Educate her and encourage her to practice exclusive breastfeeding for the 1st 6	Was the woman counselled on breastfeeding?	Was the woman counselled on breastfeeding?	1.Are you satisfied with the information you received about breastfeeding?<br><br>2. Do you are competent enough to be able to breastfeed your baby on your own?

			months of the baby's life			
2.The proportion of all postpartum women in the health facility who received contraception counselling on birth spacing and family planning before discharge.		Pre-discharge counselling for the mother and baby	1.Does the facility offer contraceptive services?<br><br>2. Is postpartum family planning services available?	Was the woman counselled on family planning?	Was the woman counselled on contraception?	Are you satisfied with the information you received on family planning?
Quality statement 1.2						
Women with pre-eclampsia or eclampsia promptly receive appropriate interventions.						
Input measures	F1.PSFR	F2.MI	F3.SIV	F4.OPCI	F5.CMRR	F6.WEICPC
1.The health facility has supplies of oral and intravenous antihypertensive agents and magnesium sulphate available in sufficient	1. Does the health facility have the Magnesium Sulphate(MgS04)?<br>2. Does the health facility have the IV Diazepam?		Is i) Injectable antibiotics administration; ii) injectable anticonvulsant administration provided in this facility as EmOC signal functions and were the	Did the health worker set an IV line on woman?<br><br>Did the woman have an IV-line access?	1. if woman received IV fluid, why was it given?<br><br>2. what was done for the woman with an obstetric complication?	1. Were you given IV fluid?<br><br>2. Were you given medicine to take?<br><br>3. Were you given injection (IM?

quantities at all times in the antenatal, labour and childbirth areas of the maternity unit			available in the past 6 months?<br><br>Vignettes:<br>2. give Magnesium Sulphate or, if not available Diazepam.			
2.The health facility has written, up-to-date clinical protocols on the management of pre-eclampsia that are available in the labour, childbirth and postnatal areas of the maternity unit and are consistent with WHO guidelines	1. Does the health facility have the Magnesium Sulphate(MgS04)?<br>2. Does the health facility have the IV Diazepam?	What are the main policies of this facility regarding maternity and newborn care? collect any points.	Have you received refresher/in-service training in the past 12 months on management of Pre-Eclampsia and/ or eclampsia?		What was done for the woman with Eclampsia complication during pregnancy?<br><br>What was done for the woman with Pre-eclampsia/ecla mpsia obstetric complication that occurred during labour and/ or delivery and/ or immediately postpartum?	1. Were you given IV fluid?<br><br>2. Were you given medicine to take?<br>3. Were you given injection (IM?<br><br>4. collapsed or convulsed?<br><br>5. you were told your BP was high?

3. Health-care staff in the maternity unit receive in-service training and regular refresher sessions in the use of antihypertensive agents, intravenous infusion and magnesium sulphate for treating pre-eclampsia and eclampsia at least once every 12 months.	1. Does the health facility have the Magnesium Sulphate(MgS04)?<br><br>2. Does the health facility have the IV Diazepam?		1.Have you received refresher, and/ or in-service training in the last 12 months on management of pre-eclampsia and/ or eclampsia?<br><br>2. Is i) Injectable antibiotics administration; ii) injectable anticonvulsant administration provided and done in past 6 months as EmOC signal functions?<br><br>3. Vignettes: give Magnesium Sulphate or, if	Did the health worker set an IV line on woman?<br><br>Did the woman have an IV-line access?	1. if woman received IV fluid, why was it given?<br>3. what was done for the woman with an obstetric complication?	1. Were you given IV fluid?<br><br>2. Were you given medicine to take?<br>3. Were you given injection (IM?

			not available Diazepam.			
Output/process measures	F1.PSFR	F2.MI	F3.SIV	F4.OPCI	F5.CMRR	F6.WEICPC
1. proportion of all women with severe pre-eclampsia or eclampsia in the health facility who received the full dose of magnesium sulphate	1. Does the health facility have the Magnesium Sulphate(MgS04)?<br>2. Does the health facility have the IV Diazepam?		Vignette: Upon examination she had a blood pressure of 170/120, 3+ protein in her urine and brisk reflexes. How would she be managed at this facility? DO NOT PROMPT!<br><br>Vignettes: give Magnesium Sulphate or, if not available Diazepam.		1.What was done for the woman with eclampsia as a pregnancy complication?<br><br>2.What was done for the woman with Pre-eclampsia /eclampsia as an obstetric problem that occurred during labour and/ or delivery and/or immediately postpartum?	
2.The proportion of all women with severe	1. Does the health facility have the Magnesium		1.Have you received refresher, and/ or in-service	Did the health worker set an IV line on woman?	1. if woman received IV fluid, why was it given?	1. Were you given IV fluid?

pregnancy-induced hypertension in the health facility who received the recommended antihypertensiv es	Sulphate(MgS04)?<br>2. Does the health facility have the IV Diazepam?		training in the last 12 months on management of pre-eclampsia and/ or eclampsia?<br><br>2. Is i) Injectable antibiotics administration; ii) injectable anticonvulsant administration provided and done in past 6 months as EmOC signal functions?<br><br>3. Vignettes: give Magnesium Sulphate or, if not available Diazepam.	Did the woman have an IV-line access?	3. what was done for the woman with an obstetric complication?	2. Were you given medicine to take?<br>3. Were you given injection (IM?
3.The proportion of all women with pre-eclampsia			Vignettes:<br>1.a 26-year-old women is 7 months	1. Time of arrival of woman	Did the woman have Hypertension as a chronic	

in the health facility whose condition progressed to eclampsia.			pregnant comes in complaining of headaches, blurred vision and epigastric pain and her face looks swollen. In this facility, what would you usually do to establish a diagnosis? DO NOT PROMPT!<br><br>2. Upon examination she had a blood pressure of 170/120, 3+ protein in her urine and brisk reflexes. How would she be managed at this facility? DO NOT PROMPT!	2. History taken<br><br>3. Blood pressure taken<br><br>4. Urine test done<br><br>5. How often were partograph filled after examination?<br><br>Was there any complication in the labour or delivery?<br><br>What complications? List	medical condition?	

Outcome measures	F1.PSFR	F2.MI	F3.SIV	F4.OPCI	F5.CMRR	F6.WEICPC
1.The proportion of all women with pre-eclampsia or eclampsia in the health facility who died as a result of pre-eclampsia or eclampsia	Does the facility routinely conduct audit for maternal deaths?	Facility has specific management policies on systematic mortality audits?			If maternal death, what was the main underlying cause?	
2.The proportion of all women with pre-eclampsia or eclampsia who arrived at the health facility with a live foetus whose baby died in the perinatal period (stillbirths or early neonatal deaths).	1.Does the facility routinely conduct audit for early neonatal deaths?<br><br>2. Does the facility routinely conduct audit for stillbirths?	1.Facility has specific management policies on systematic mortality audits?<br><br>2. Facility has specific management policies on systematic "near miss" audits?		1.Newborn not breathing spontaneously after birth?<br><br>2. If newborn does not begin to breath spontaneous after bag and mask ventilation, and effective ventilation, did the newborn receive	If stillbirth/early neonatal death, what was the primary underlying cause of death?	

				advanced resuscitation?		
3.The proportion of all women with pre-eclampsia or eclampsia in the health facility who experienced maternal near-misses due to pre-eclampsia or eclampsia	Does the facility routinely conduct audit for maternal deaths?	1.Facility has specific management policies on systematic mortality audits?<br><br>2. Facility has specific management policies on systematic "near miss" audits?	Vignette:<br>Upon examination she had a blood pressure of 170/120, 3+ protein in her urine and brisk reflexes. How would she be managed at this facility? DO NOT PROMPT!		1. Did the woman have any chronic medical condition? specify<br><br>3. which of the complication did the woman suffer from during pregnancy?	
Quality statement 1.3						
Women with postpartum haemorrhage promptly receive appropriate interventions						
Input measures	F1.PSFR	F2.MI	F3.SIV	F4.OPCI	F5.CMRR	F6.WEICPC
1.The health facility has written, up-to-date clinical protocols for post-partum haemorrhage		What are the main policies of this facility regarding maternity and newborn care?	Have you received refresher/in-service training in the past 12 months on management of		Did postpartum haemorrhage as an obstetric problem occur during labour, and/ or delivery, and/ or	Did you have any complication after birth due to excessive bleeding that wet your

management that are available in the childbirth and postnatal care areas and are consistent with WHO guidelines		collect any points.	obstetric haemorrhage (ante-and postpartum)?		immediately postpartum?	clothes, poured on the floor, needed transfusion with blood or needed operation again?
2.The health facility has uterotonic drugs and supplies for intravenous fluid and blood administration (syringes, needles, intravenous cannulas, intravenous fluid solutions, blood) available in sufficient quantities at all times in the childbirth and postnatal care areas.	1. Does the facility provide IV stand, IV fluid, IV canula, IV antibiotics?<br><br>2. Does the facility have means of hanging IV fluids, drugs or blood inside the ambulance (referral care)?		1.Was i) Injectable antibiotics; ii) injectable oxytocic(Synto/ Ergo) drugs administration, iii) injectable anticonvulsant administration; iv) blood transfusion services; provided in this facility in the past 6 months<br><br>2. Was Comprehensive	1.Was her blood sample taken?<br><br>2. Did the health worker set an IV line on woman?	1. did woman receive IV fluid?<br><br>2.Did the woman receive any antibiotics?<br><br>3. what antibiotics was given?<br><br>4. did the newborn receive antibiotic?<br><br>5. Baby(ies) received IV fluids?	Were you given any of the i) IV fluid;<br>ii) medicine to take;<br>iii) Injection (IM)

			IV fluid administration done in the past 6 months?<br><br>3. Was management of suspected sepsis with Injectable antibiotics (Ampicillin/Penicillin and Gentamicin) done in the past 6 months.<br><br>4. Vignettes: Give IV fluids rapidly/Give blood transfusion		6. if baby received IV fluid, what IV fluid was given?	
3. A functional blood transfusion service is available in the health facility at all times	1.Does the facility provide the laboratory services?<br><br>2. Does the facility have	Is there a form of external calibration of equipment used in this facility such as Laboratory	1.Was i) blood transfusion services; provided in this facility in the past 6 months			

	means of hanging IV fluids, drugs or blood?<br><br>3.Does the facility have IV stand, IV fluid, IV canula?	equipment, glucometers?	2. Was Comprehensive IV fluid administration done in the past 6 months?<br><br>3. Vignettes: Give blood transfusion			
4.Health-care staff in the labour, childbirth and postnatal care areas of the maternity unit receive in-service training and regular refresher sessions in management of post-partum haemorrhage at least once every 12 months		1.What additional training is provided to workers posted in maternity and children's ward?<br><br>2. are there refresher training for the staff?<br><br>3. have there been training	Have you received in-service training, and/ or refresher in the management of obstetric haemorrhage (ante-and-postpartum) in the last 12 months?	1.Was there any complications in the labour and the delivery?<br><br>2. what complications? List (tears, rupture, PPH, obstruction, etc)	What was done with the woman with a Postpartum haemorrhage obstetric problem which occurred during labour, and/ or delivery, and/ or immediate postpartum?	Did you have any complication after birth due to excessive bleeding that wet your clothes, poured on the floor, needed transfusion with blood or needed operation again?

		over the past year?<br><br>4. how often have they been trained over the past year.				
Output/process measures	F1.PSFR	F2.MI	F3.SIV	F4.OPCI	F5.CMRR	F6.WEICPC
1.The proportion of all women with post-partum haemorrhage in the health facility who received therapeutic uterotonic drugs.	Oxytocic (syntomentrine/ Ergot)		Is injectable Oxytocic (Synto/Ergo) drugs administration performed in this facility as EmOC signal functions and was it done in the past 6 months?		What was done for a woman with postpartum haemorrhage that occurred during labour and/or delivery and / or immediately postpartum?	
2.The proportion of all women in the health facility with post-partum			1.Is manual removal of retained placenta, and manual removal of retained		What was done for a woman with an obstetric complication that occurred	Did you have any complication after birth due to excessive bleeding that

haemorrhage due to a retained placenta for whom manual removal of the placenta was performed by a skilled birth attendant			products of conception provided in this facility and were they performed in the past 6 months?<br><br>2. have you received refresher/in-service training in the past 12 months on active management of third stage of labour?		during labour, and/ or delivery, and/ or immediate postpartum?	wet your clothes, poured on the floor, needed transfusion with blood or needed operation again?
Outcome measures	F1.PSFR	F2.MI	F3.SIV	F4.OPCI	F5.CMRR	F6.WEICPC
1.The proportion of all women who had post-partum haemorrhage in the health facility who died	Does the facility routinely conduct audit for maternal deaths?	1.Facility specific management policies on i) systematic mortality audits			1.Delivery outcome of the woman?<br><br>2.If maternal death, what was the main	

as a result of post-partum haemorrhage.		2. Does your facility have a quality improvement programme, which includes quality improvement teams, capturing and use of data, and monitoring and evaluation			underlying cause?	
2.The proportion of all women who gave birth in the health facility by caesarean section who received a blood transfusion	Does this facility provide laboratory services?<br><br>Does the inside of an ambulance have means of hanging IV fluids, drugs or blood?		1.Is blood transfusion services provided in this facility and was it done in the past 6 months?<br><br>2. Vignette: Give blood transfusion		1.Type of delivery<br><br>2.Was it an emergency or elective C/S?<br><br>3.Did the women receive IV fluid?<br><br>4.If woman received IV fluid why was it given?	Type of delivery<br><br>Did the woman have any complication after the birth with excessive bleeding that wet your clothes, poured on the floor, needed transfusion with blood or needed

					5.If woman received IV fluid which fluid was given?	operation again?
3.The proportion of all women who gave birth vaginally in the health facility who received a blood transfusion	Does this facility provide laboratory services?<br><br>Does the inside of an ambulance have means of hanging IV fluids, drugs or blood?		1.Is blood transfusion services provided in this facility and was it done in the past 6 months?<br><br>2. Vignette: Give blood transfusion		1.Type of delivery<br>2.Was it an emergency or elective C/S?<br>3.Did the women receive IV fluid?<br>4.If woman received IV fluid why was it given?<br>5.If woman received IV fluid which fluid was given?	Did the woman have any complication after the birth with excessive bleeding that wet your clothes, poured on the floor, needed transfusion with blood or needed operation again?
4.The proportion of all women who gave birth in the health facility who had severe post-partum	Does this facility provide laboratory services?		1.Is blood transfusion services provided in this facility and was it done in the past 6 months?	1.Was there any complications in the labour and the delivery?	1.Type of delivery<br>2.Was it an emergency or elective C/S?	Excessive bleeding that wet your clothes, poured on the floor, needed transfusion with

haemorrhage (abnormal bleeding of >1000 mL or any bleeding with hypotension or requiring blood transfusion).	Does the inside of an ambulance have means of hanging IV fluids, drugs or blood?		2. Vignette: Give blood transfusion	2. what complications? List (tears, rupture, PPH, obstruction, etc)	3.Did the women receive IV fluid?<br>4.If woman received IV fluid why was it given?<br>5.If woman received IV fluid which fluid was given?	blood or needed operation again?
5.The proportion of all women who had post-partum haemorrhage in the health facility who experienced maternal near-misses due to post-partum haemorrhage.	Does this facility routinely conduct audit for maternal deaths?	Facility specific management policies on systematic ”near miss” audits		1.Was there any complications in the labour and the delivery?<br><br>2. what complications? List (tears, rupture, PPH, obstruction, etc)	1. Did Postpartum haemorrhage occur during labour and/ or delivery and/ or immediately postpartum as an obstetric complication?<br>2.Delivery outcome of the woman?	Excessive bleeding that wet your clothes, poured on the floor, needed transfusion with blood or needed operation again?
Quality statement 1.4						
Women whose process in labour is delayed or whose labour is obstructed receive appropriate interventions, according to WHO guidelines.						

Input measures	F1.PSFR	F2.MI	F3.SIV	F4.OPCI	F5.CMRR	F6.WEICPC
1.The health facility has written, up-to-date clinical protocols for preventing and managing prolonged labour, which are available in the labour and childbirth areas and are consistent with WHO guidelines		What are the main policies of this facility regarding maternity and newborn care? collect any points.	Have you received refresher/in-service training in the management of prolonged or obstructed labour?	1.Was there any complications in the labour and the delivery?<br><br>2. what complications? List (tears, rupture, PPH, obstruction, etc)	What was done for the woman with an obstruction obstetric complication?	
2.The health facility has the essential supplies and equipment for vacuum or forceps-assisted delivery, including newborn resuscitation equipment,	1. Does the facility has functioning i) delivery forceps?<br>ii) vacuum aspirator?<br>iii) bag and mask (for babies)?<br>iv) spot light?		Was Assisted vaginal/instrumental (Vacuum or forceps) delivery as a signal EmOC function provided in this facility and was it done in past 6 months?	1.Plan for delivery communicated to the mother (SVD/Assisted vaginal delivery/C/S)<br><br>2. List the items in the resuscitation area (clean	What was done with baby(ies) with Asphyxia as a baby complication?	Type of delivery (i. spontaneous vaginal delivery; ii. Assisted with Vacuum/instruments; iii. Caesarean Section)

available in sufficient quantities at all times in the childbirth area of the maternity unit	v) resuscitation tray in the ambulance?		Vignette:<br>2. Prepare to resuscitate the baby<br><br>3. use bag and mask to ventilate if baby does not cry after sanctioning.<br><br>4. Apply cardiac message if ventilation alone does not help.	towels – at least x2, ambu bag, neonatal sized mask, radiant warmer, buld syringe for aspiration of fluids, oxygen cylinder with oxygen).<br><br>3. Check delivery trolley and instruments for their functioning status		
3.The health facility has an adequately equipped operating theatre close to the childbirth area of the maternity unit	1. Does the facility have a functional theatre?		Was caesarean section delivery as a signal EmOC function provided in this facility and was it done in the past 6 months?	Plan for delivery communicated to the mother (SVD/Assisted vaginal delivery/C/S)	Was it an emergency or elective C/S?<br><br>What was the indication for the C/S?	Type of delivery (i. spontaneous vaginal delivery; ii. Assisted with Vacuum/instru ments; iii. Caesarean Section)

4.The health facility has an adequate number of staff skilled in performing caesarean section, 24 h a day	1. Level of the facility?<br>2. How many of the following professionals (neonatologist/obstetrician & gynaecologist/paediatrician/medical officers/midwives/nurses) do you have in this facility?<br>3. how many professionals are in the maternity ward?	1. What additional training is provided to workers posted in maternity and children's wards?<br><br>2. Are there additional refresher training for staff?	Was caesarean section delivery as a signal EmOC function provided in this facility and was it done in the past 6 months?	Plan for delivery communicated to the mother (SVD/Assisted vaginal delivery/C/S)	Was it an emergency or elective C/S?<br><br>What was the indication for the C/S?	Type of delivery (i. spontaneous vaginal delivery; ii. Assisted with Vacuum/instruments; iii. Caesarean Section)
5.Health-care staff in the labour and childbirth areas of the maternity unit receive in-service training and regular refresher sessions (every		1. What are the minimum qualifications to work in the maternity ward?<br><br>2. What additional training is provided to	Have you received refresher/in-service training in the management of prolonged or obstructed labour?	1.Was there any complications in the labour and the delivery?<br><br>2. what complications? List (tears,	What was done for the woman with an obstruction obstetric complication?	

6 months) in managing prolonged and obstructed labour.		workers posted in maternity and children's wards?<br><br>3. Are there additional refresher training for staff?		rupture, PPH, obstruction, etc)		
Output/process measures	F1.PSFR	F2.MI	F3.SIV	F4.OPCI	F5.CMRR	F6.WEICPC
1.The proportion of all nulliparous women in the health facility with a singleton cephalic foetus at ⩾ 37 weeks' gestation who underwent caesarean section during spontaneous labour (Robson group			1.How many years of formal training have you received in the care of pregnant women?<br><br>2. Is caesarean delivery provided in this facility and was it performed in the past 6 months?	1.Gestational age of the pregnancy<br><br>2. Plan for delivery communicated to mother (SVD/Assisted vaginal delivery/C/S)	1.Gravida of woman?<br>2.Parity of the woman?<br>3.Number of completed gestational weeks of gestation at delivery? What type of delivery did she have?<br>4.Birth weight of	1. Number of all previous pregnancies (including this one).<br><br>2. Number of all previous deliveries (including this one).<br><br>3. Type of delivery (i. spontaneous

			3. Vignettes:<br>3.1 Prepare for caesarean section<br><br>3.2 Refer to hospital where caesarean section can be done		baby1/baby2/baby3?	vaginal delivery; ii. Assisted with Vacuum/instruments; iii. Caesarean Section).
2.The proportion of all women in the health facility with prolonged and/or obstructed labour who gave birth by caesarean section	Does the facility have a functional theatre?		1.Is caesarean delivery provided in this facility and was it performed in the past 6 months?<br><br>2. Have you received refresher/in-service training in the past 12 months on management of prolonged or	Plan for delivery communicated to mother (SVD/Assisted vaginal delivery/C/S)	1.Obstructed labour during labour and/or delivery?<br>2.What type of delivery did she have?<br>What was the 3.indication for the C/S?	If caesarean section, was it already planned during pregnancy?

			obstructed labour			
3. The proportion of all women who gave birth in the health facility who underwent instrumental vaginal birth for delayed second stage of labour	Does the facility has functioning i) delivery forceps?<br>ii) vacuum aspirator?		Was Assisted vaginal/instrumental (Vacuum or forceps) delivery as a signal EmOC function provided in this facility and was it done in the past 6 months?	1.Plan for delivery communicated to the mother (SVD/Assisted vaginal delivery/C/S)<br><br>2. Check delivery trolley and instruments for their functioning status	Type of delivery (i. Spontaneous vaginal delivery, ii. Assisted vaginal delivery (vacuum or forceps), iii. A planned Caesarean section delivery, iv. An emergency caesarean section delivery	Type of delivery (i. spontaneous vaginal delivery; ii. Assisted with Vacuum/instruments; iii. Caesarean Section)
4.The proportion of women with prolonged or obstructed labour who underwent emergency caesarean section within 30 min of the	Does the facility have a functional theatre?		1.Is caesarean delivery provided in this facility and was it performed in the past 6 months?<br><br>2. Have you received refresher/in-	1. Feedback to mother after examination?<br><br>2. Plan for delivery communicated to the mother (SVD/Assisted vaginal delivery/C/S)	1.What type of delivery did she have? (i. Spontaneous vaginal delivery, ii. Assisted vaginal delivery (vacuum or forceps), iii. A planned Caesarean	

decision to perform caesarean section			service training in the past 12 months on management of prolonged or obstructed labour		section delivery, iv. An emergency caesarean section delivery,<br><br>2. How long (hours and minutes) after the decision for the CS was the actual surgery performed?<br><br>3. if the delay was more than 30 minutes, what was the main cause of the delay?	
5.The proportion of all women in the health facility with confirmed delay in progress of the first stage of	Does the facility have Oxytocic (Syntometrine/Ergot) as essential drugs?		Is injectable oxytocic (Synto/Ergo) drugs administration as signal EmOC functions provided in this	1.Was labour augmented at any time?<br><br>2. Oxytocin administered?	1.Was the labour augmented?<br>2.Was the woman given parenteral oxytocin?	During labour were you given any IV fluid/medicine to take/injection (IV)?

labour who received oxytocin for augmentation.			facility and was it done in the past 6 months?			
6.The proportion of all women who gave birth in the health facility whose active phase of first stage of labour exceeded 12 h			Have you received refresher/in-service training in the past 12 months on i) labour monitoring; ii) partograph use?	1.Time of arrival of woman?<br><br>2. Vaginal examination done<br><br>3. Is the woman in labour?<br><br>4. started a partograph in the labour ward?<br><br>5. Record time of delivery	1. Date of presentation at the health facility?<br>2. Time of presentation at the health facility?<br>3. At what stage of labour did the women present?<br>4. Was a partograph used to monitor labour?<br>5. Date of delivery at the health facility?<br>Time of delivery at the health facility?	
7.The proportion of all women who			Have you received refresher/in-	1.Time of arrival of woman?	1.Was a partograph used	

gave birth in the health facility who had a prolonged second stage of labour.			service training in the past 12 months on the management of prolonged or obstructed labour?	2. Is the woman in labour?<br><br>3. started a partograph in the labour ward?<br><br>4. Record time of delivery	to monitor labour?<br><br>2. At what stage of labour did the women present?<br><br>3. Date and time partograph was started?<br><br>4. How many times was a vaginal examination carried out and recorded between first exam and delivery?<br><br>5. according to the partograph when did the woman deliver?	
Outcome measures	F1.PSFR	F2.MI	F3.SIV	F4.OPCI	F5.CMRR	F6.WEICPC

1.The proportion of all women who gave birth in the health facility whose uterus ruptured during labour				Was there any complications in the labour and the delivery?<br><br>What complications? List (tears, rupture, PPH, obstruction, etc).	Which of the obstetric problems occurred during labour and/or delivery and/or immediately postpartum?	
2.The proportion of all perinatal deaths that occurred in the health facility after prolonged or obstructed labour	Does this facility routinely conduct audit for early neonatal deaths?<br><br>Does this facility routinely conduct audit for stillbirths?	Facility specific policies on systematic mortality audits	Have you received refresher/in-service training in the past 12 months on Asphyxia/resuscitation?	1.Spontaneous breathing assessed at birth?<br><br>2. Newborn not breathing spontaneously at birth.<br><br>3. if newborn does not begin to breath spontaneously after bag and mask	1.Which of the following obstetric problems occurred during labour and/or delivery and/or immediately postpartum?<br>2. Delivery outcome of the newborn?	

				ventilation, and effective ventilation, did the newborn received advanced resuscitation?		
3.The proportion of all newborns born in the health facility who had birth injuries (brachial palsy, fractures, cephalhematom a).		Facility specific policies on systematic "near miss" audits			If live birth with complications (whether alive or dead), what complication did the baby (ies) have?	
4.The proportion of women with prolonged and/or obstructed labour in the health facility who experienced maternal near-	Does this facility routinely conduct audit for maternal deaths?	Facility specific policies on systematic "near miss" audits			1.Which of the obstetric problems occurred during labour and/or delivery and/or immediately postpartum?<br>2. Delivery outcome of the mother?	

misses due to prolonged and/or obstructed labour.						
Quality statement 1.5						
Newborns who are not breathing spontaneously receive appropriate stimulation and resuscitation with a bag and mask within 1 min of birth, according to WHO guidelines						
Input measures	F1.PSFR	F2.MI	F3.SIV	F4.OPCI	F5.CMRR	F6.WEICPC
1.The health facility has suction device, at least two sizes of neonatal mask and a self-inflating bag in the childbirth and neonatal areas of the maternity unit	1. The facility has functioning Bag and mask (for babies)<br><br>2.The facility has functioning Sucker machine	Does the training sessions for workers in maternity and children's ward cover?<br>i. newborn resuscitation, ii. Essential newborn care	Vignettes:<br>Give oxygen by mask or nasal cannulae<br><br>Prepare to resuscitate the baby<br><br>Examine and suction the mouth<br><br>Use bag and mask to ventilate if baby does not cry after suctioning	List the items in the resuscitation area: i) ambu bag, ii) neonatal sized mask	If live birth with complications (whether alive or dead), did the baby(ies) have Asphyxia as a complication?	

2. The health facility has written, up-to-date clinical protocols for managing newborns who are not breathing spontaneously in the childbirth areas of the maternity unit that are consistent with WHO guidelines.	Does the facility have functioning i. examination table, ii. filled oxygen cylinder, iii. oxygen cylinder carrier, iv. bag and mask (for babies), iv. radiant warmers	What are the main policies of this facility regarding maternity and newborn care? collect any points.<br><br>Does the training sessions for workers posted in maternity and children's ward cover? i. management of birth complications, ii. Newborn resuscitation, iii. Essential newborn care	1.Have you received in-service training, and/ or refresher in the management of asphyxia/resuscitation in the last 12 months?<br><br>2.Vignettes:<br>2.1 Give oxygen by mask or nasal cannulae<br><br>2.2 Prepare to resuscitate the baby<br><br>2.3 Use bag and mask to ventilate if baby does not cry after suctioning	1.Spontaneous breathing assessed at birth?<br><br>2. Newborn not breathing spontaneously at birth.<br><br>3. if newborn does not begin to breath spontaneously after bag and mask ventilation, and effective ventilation, did the newborn received advanced resuscitation?<br><br>4. list the items in the resuscitation area: i. ambu	If live birth with complications (whether alive or dead), did the baby(ies) have Asphyxia as a complication?	

				bag, ii. Neonatal sized mask		
3. All health-care workers providing care for pregnant and postpartum women and newborns in the health facility are skilled in basic newborn resuscitation, as demonstrated by simulating positive-pressure ventilation with a bag-and-mask on a manikin.	Does the facility have functioning i. examination table, ii. filled oxygen cylinder, iii. oxygen cylinder carrier, iv. bag and mask (for babies), iv. radiant warmers	Does the training sessions for workers posted in maternity and children's ward cover? i. management of birth complications, ii. Newborn resuscitation, iii. Essential newborn care	1.Have you received in-service training, and/ or refresher in the management of asphyxia/resuscitation in the last 12 months?<br><br>2.Vignettes:<br>2.1 Give oxygen by mask or nasal cannulae<br><br>2.2 Prepare to resuscitate the baby<br><br>2.3 Use bag and mask to ventilate if baby does not cry after suctioning	1.Spontaneous breathing assessed at birth?<br><br>2. Newborn not breathing spontaneously at birth.<br><br>3. if newborn does not begin to breath spontaneously after bag and mask ventilation, and effective ventilation, did the newborn received advanced resuscitation?<br><br>4. list the items in the	If live birth with complications (whether alive or dead), did the baby(ies) have Asphyxia as a complication?	

				resuscitation area: i. ambu bag, ii. Neonatal sized mask		
4. Health care staff in the childbirth and neonatal areas of the maternity unit receive in-service training and regular refresher sessions s in basic newborn resuscitation at least once every 12 months.	Does the facility have functioning i. examination table, ii. filled oxygen cylinder, iii. oxygen cylinder carrier, iv. bag and mask (for babies), iv. radiant warmers	Does the training sessions for workers posted in maternity and children's ward cover? i. management of birth complications, ii. Newborn resuscitation, iii. Essential newborn care	1.Have you received in-service training, and/ or refresher in the management of asphyxia/resuscitation in the last 12 months?<br><br>2.Vignettes:<br>2.1 Give oxygen by mask or nasal cannulae<br><br>2.2 Prepare to resuscitate the baby<br><br>2.3 Use bag and mask to ventilate if baby	1.Spontaneous breathing assessed at birth?<br><br>2. Newborn not breathing spontaneously at birth.<br><br>3. if newborn does not begin to breath spontaneously after bag and mask ventilation, and effective ventilation, did the newborn received advanced resuscitation?	If live birth with complications (whether alive or dead), did the baby(ies) have Asphyxia as a complication?	

			does not cry after suctioning	4. list the items in the resuscitation area: i. ambu bag, ii. Neonatal sized mask		
5.Health care staff in the childbirth and neonatal areas of the maternity unit receive monthly drills or simulation exercises and supportive supervision in basic newborn resuscitation.	Does the facility have functioning i. examination table, ii. filled oxygen cylinder, iii. oxygen cylinder carrier, iv. bag and mask (for babies), iv. radiant warmers	Does the training sessions for workers posted in maternity and children's ward cover? i. management of birth complications, ii. Newborn resuscitation, iii. Essential newborn care	1.Have you received in-service training, and/ or refresher in the management of asphyxia/resuscitation in the last 12 months?<br><br>2.Vignettes:<br>2.1 Give oxygen by mask or nasal cannulae<br><br>2.2 Prepare to resuscitate the baby<br><br>2.3 Use bag and mask to	1.Spontaneous breathing assessed at birth?<br><br>2. Newborn not breathing spontaneously at birth.<br><br>3. if newborn does not begin to breath spontaneously after bag and mask ventilation, and effective ventilation, did the newborn received	If live birth with complications (whether alive or dead), did the baby(ies) have Asphyxia as a complication?	

			ventilate if baby does not cry after suctioning	advanced resuscitation?<br><br>4. list the items in the resuscitation area: i. ambu bag, ii. Neonatal sized mask		
Output/process measures	F1.PSFR	F2.MI	F3.SIV	F4.OPCI	F5.CMRR	F6.WEICPC
1.The proportion of all newborns who were not breathing spontaneously after additional stimulation at the health facility who were resuscitated with a bag-and-mask	Does the facility have functioning i. bag and mask (for babies), iv. radiant warmers	Does the training sessions for workers posted in maternity and children's ward cover? i. Newborn resuscitation, iii. Essential newborn care	1.Have you received in-service training, and/ or refresher in the management of asphyxia/resuscitation in the last 12 months?<br><br>2.Vignettes:<br>2.1 Give oxygen by mask or nasal cannulae	1.Spontaneous breathing assessed at birth?<br><br>2. Newborn not breathing spontaneously at birth.<br><br>3. if newborn does not begin to breath spontaneously after bag and mask	If live birth with complications (whether alive or dead), did the baby(ies) have Asphyxia as a complication?	

			2.2 Prepare to resuscitate the baby<br><br>2.3 Use bag and mask to ventilate if baby does not cry after suctioning	ventilation, and effective ventilation, did the newborn received advanced resuscitation?		
2. The proportion of all newborns who were not breathing spontaneously after additional stimulation at the health facility who were resuscitated with a bag-and-mask within 1 min of birth.	Does the facility have functioning i. bag and mask (for babies), iv. radiant warmers	Does the training sessions for workers posted in maternity and children's ward cover? i. Newborn resuscitation, iii. Essential newborn care	1.Have you received in-service training, and/ or refresher in the management of asphyxia/resuscitation in the last 12 months?<br><br>2.Vignettes:<br>2.1 Give oxygen by mask or nasal cannulae<br><br>2.2 Prepare to resuscitate the baby	1.Spontaneous breathing assessed at birth?<br><br>2. Newborn not breathing spontaneously at birth.<br><br>3. if newborn does not begin to breath spontaneously after bag and mask ventilation, and effective ventilation, did	If live birth with complications (whether alive or dead), did the baby(ies) have Asphyxia as a complication?	

			2.3 Use bag and mask to ventilate if baby does not cry after suctioning	the newborn received advanced resuscitation?<br><br>4. list the items in the resuscitation area: i. ambu bag, ii. Neonatal sized mask		
Outcome measures	F1.PSFR	F2.MI	F3.SIV	F4.OPCI	F5.CMRR	F6.WEICPC
1.The proportion of all live babies at term (≥37 weeks) with no major congenital malformations in the health facility who died within 7 days of birth (early neonatal mortality).	Does this facility routinely conduct audit for early neonatal deaths?<br><br>Does this facility routinely conduct audit for stillbirths?	Facility specific policies on systematic mortality review			1.Number of completed weeks of gestation at delivery?<br><br>2. if stillbirth/early neonatal death, what was the primary cause of death?	

2.The proportion of all live babies born at term (⩾ 37 weeks) in the health facility who were not breathing spontaneously but were breathing spontaneously 5 min after resuscitation.	Does the facility have functioning i. bag and mask (for babies), iv. radiant warmers	Does the training sessions for workers posted in maternity and children's ward cover? i. Newborn resuscitation, iii. Essential newborn care	1.Have you received in-service training, and/ or refresher in the management of asphyxia/resuscitation in the last 12 months?	1. gestational age of the pregnancy<br><br>2. Spontaneous breathing assessed at birth?<br><br>3. Newborn not breathing spontaneously at birth.<br><br>4. if newborn does not begin to breath spontaneously after bag and mask ventilation, and effective ventilation, did the newborn received advanced resuscitation?	1. number of completed weeks of gestation at delivery?<br><br>2. If live birth with complications (whether alive or dead), did the baby(ies) have Asphyxia as a complication?	

Quality statement 1.6a						
Women in preterm labour receive appropriate interventions for both themselves and their babies, according to WHO guidelines.						
Input measures	F1.PSFR	F2.MI	F3.SIV	F4.OPCI	F5.CMRR	F6.WEICPC
1.The health facility has written, up-to-date clinical protocols for management of preterm labour in the childbirth areas of the maternity unit that are consistent with WHO guidelines	Facility has Dexamethasone /Betamethasone (parenteral) as essential drugs	What are the main policies of this facility regarding maternity and newborn care? collect any points.	Is surfactant provided in this facility as comprehensive functions and was it provided for in the past 6 months?			
2. The health facility has supplies of antenatal corticosteroids (dexamethasone or betamethasone) , antibiotics and magnesium sulphate	Health facility has availability of essential drugs (IV antibiotics, Magnesium Sulphate (MgS04), Diazepam, Oxytocic (syntometrine/E		1.Is injectable antibiotics administration signal EmOC functions provided in this facility and was it done in the past 6 months?<br><br>2. Vignette:		1. what was/were done with the baby(ies) with complications?<br><br>2. Did the newborn(s) receive any antibiotic?	Were you given any of the following during labour?<br>IV fluid/medicine to take/injection (IM)

available in sufficient quantities at all times to manage preterm birth in accordance with WHO guidelines	rgot), Dexamethasone /Betamethasone (parenteral), Ampicillin/Penicillin for IV, Gentamicin for IV)		Give Magnesium Sulphate or if not available, Diazepam		3. what antibiotics was given?<br><br>4. what was done with a woman with a complication during pregnancy?	
3. Health-care staff in the maternity unit receive in-service training and regular refresher sessions in the management of preterm labour at least once every 12 months		1. Have there been training over the past year?<br><br>4. How often have they been trained over the past year?<br><br>5. Do the training session cover Essential newborn care?	Have you had formal training in the management of sick or low birthweight newborns?<br><br>Have you received refresher/in-service training in the past 12 months on Kangaroo Mother Care?		1.If live birth with complications (whether alive or dead), did the baby(ies) have preterm birth as a complication?<br><br>2. what was/were done with the baby(ies) with the above complications?	

4. Health-care staff in the maternity unit receive monthly drills or simulation exercises and supportive supervision in the management of preterm labour.		1.What additional training is provided to workers posted in the children's ward?<br>2. Did the training in the past year cover management of birth complications?	Have you had formal training in the management of sick or low birthweight newborns?<br><br>Have you received refresher/in-service training in the past 12 months on Kangaroo Mother Care?		1.If live birth with complications (whether alive or dead), did the baby(ies) have preterm birth as a complication?<br><br>2. what was/were done with the baby(ies) with the above complications?	
Output/process measures	F1.PSFR	F2.MI	F3.SIV	F4.OPCI	F5.CMRR	F6.WEICPC
1.The proportion of all preterm newborns born between 24 and 34 weeks of gestation in the health facility	Essential drugs: Dexamethasone /Betamethasone (parenteral)			1.Gestational age of the pregnancy<br><br>2. what was the weight of the baby?	1.Number of completed weeks of gestation at delivery?<br><br>2. What was done for a	

whose mothers received at least one dose of antenatal corticosteroids when indicated					woman with a pregnancy related complication.<br><br>3. What was done for a woman with an obstetric complication during labour/delivery or immediate postpartum?	
2. The proportion of all preterm newborns born before 32 weeks of gestation in the health facility whose mothers received magnesium sulphate to protect their baby from foetal	Essential drugs: Magnesium Sulphate (MgS04)			1.Gestational age of the pregnancy<br><br>2. what was the weight of the baby?	1.Number of completed weeks of gestation at delivery?<br><br>2. What was done for a woman with a pregnancy related complication.	

neurological complications					3. What was done for a woman with an obstetric complication during labour/delivery or immediate postpartum?<br><br>4. What was done with the woman with the above obstetric complication(s)	
3. The proportion of all women with preterm pre-labour rupture of membranes who gave birth in the health facility who received prophylactic antibiotics.	Essential drugs: IV antibiotics		Is injectable antibiotics administration provided in this facility and was it done in the past 6 months?	1. Was there any complications in the labour and the delivery?<br><br>2. What complications? List (tears, rupture, PPH, Obstruction, etc).	1. Number of completed weeks of gestation at delivery?<br><br>2. What was done for a woman with an obstetric complication during labour/delivery	1. Did you break water before coming to the facility?<br><br>2. For how long your water broke before coming to the facility?<br><br>3. Were you given any of the

					or immediate postpartum?	following during labour? IV fluid, medicine to take, injection (IM)
Outcome measures	F1.PSFR	F2.MI	F3.SIV	F4.OPCI	F5.CMRR	F6.WEICPC
1.The proportion of all women with preterm pre-labour rupture of membranes who gave birth in the health facility who received prophylactic antibiotics	Essential drugs: IV antibiotics		Is injectable antibiotics administration provided in this facility and was it done in the past 6 months?	Was there any complications in the labour and the delivery?<br><br>What complications? List (tears, rupture, PPH, Obstruction, etc).	1.Number of completed weeks of gestation at delivery?<br><br>2. What was done for a woman with an obstetric complication during labour/delivery or immediate postpartum?	1. Did you break water before coming to the facility?<br><br>2. For how long your water broke before coming to the facility?<br><br>3. Were you given any of the following during labour? IV fluid, medicine to take, injection (IM)

2.The proportion of all babies at risk for birth in the health facility at 24 to < 34 weeks of gestation who were exposed to antenatal corticosteroids	Essential drugs: Dexamethasone /Betamethasone (parenteral)			1.Gestational age of the pregnancy<br><br>2. what was the weight of the baby?	1.Number of completed weeks of gestation at delivery?<br><br>2. What was done for a woman with a pregnancy related complication.<br><br>3. What was done for a woman with an obstetric complication during labour/delivery or immediate postpartum?	
3.The proportion of all babies at risk for birth in the health facility at ≥37 weeks of	Essential drugs: Dexamethasone /Betamethasone (parenteral)			1.Gestational age of the pregnancy	1.Number of completed weeks of gestation at delivery?	

gestation who were exposed to antenatal corticosteroids.				2. what was the weight of the baby?	2. What was done for a woman with a pregnancy related complication.<br>3. What was done for a woman with an obstetric complication during labour/delivery or immediate postpartum?	
Quality statement 1.6b						
Preterm and small babies receive appropriate care, according to WHO guidelines						
Input measures	F1.PSFR	F2.MI	F3.SIV	F4.OPCI	F5.CMRR	F6.WEICPC
1.The health facility has written, up-to-date clinical protocols for the care of small and preterm babies in the childbirth areas	Does this facility provide services for Neonatal Intensive Care Unit?<br><br>Does this facility provide	1.What are the main policies of this facility regarding maternity and newborn care? collect any points.	1.Does this facility have dedicated space for Kangaroo Mother Care? if yes, was it done in the past 6 months?		What was/were done for the baby(ies) with preterm birth complications?	

of the maternity unit that are consistent with WHO guidelines	services for Kangaroo Mother Care ward/beds?	2. Facility specific management policies on i. Baby friendly, ii. Early Initiation of breastfeeding, iii. KMC for babies	2. Does this facility have recorded evidence of KMC (prolonged STSC, EBF/feeding support, growth monitoring (weight)? If yes, was it done in the past 6 months?<br><br>3. does this facility provide newborn intensive care unit? if yes, was it done/provided in the past 6 months?<br><br>4. is surfactant provided in this facility and was it provided in			

			the past 6 moths?			
2. The health facility has supplies and materials to provide optimal thermal care to stable and unstable preterm babies, including kangaroo mother care (support binders, baby hats, socks), clean incubators and radiant warmers	1. Does the facility provide services on NICU, KMC ward/beds?<br><br>2. Does the facility have functioning incubators, radiant warmers and thermometer-digital/mercury column?	Facility specific management policies on i. Early Initiation of breastfeeding, ii. KMC for babies	1.Is temperature provided in this facility as Newborn signal function and was it done in the past 6 months?<br><br>2. Does this facility have dedicated space for Kangaroo Mother Care? if yes, was it done in the past 6 months?<br><br>3. Does this facility have recorded evidence of KMC (prolonged STSC, EBF/feeding support, growth	List the items in the resuscitation area:<br>1.Clean towels-at least 2<br><br>2. Radiant warmers<br><br>Immediate care of the newborn:<br>3. Baby dried immediately and thoroughly;<br><br>4. Baby placed skin to skin contact with the mother immediately after birth<br><br>5. Time baby was first put to breast	What was/were done for the baby(ies) with preterm birth complications?	

			monitoring (weight)? If yes, was it done in the past 6 months?<br><br>4. does this facility provide newborn intensive care unit? if yes, was it done/provided in the past 6 months?	6. Baby's temperature taken after birth<br><br>7. when was the baby first taken out of skin to skin contact with mother.		
3. The health facility has the supplies and materials to provide optimal feeding to preterm babies and support for breastfeeding or alternative feeding (feeding cups and spoons, infant	1. Does the facility provide services on NICU?<br>2. KMC ward/beds?<br><br>3. autoclave for sterilization?<br><br>4. hand washing station?	Facility specific management policies on i. Baby friendly, ii. Early Initiation of Breastfeeding, iii. Rooming in	1.Is Early Initiation and Exclusive breastfeeding as newborn signal function provided in this facility and was it done in the past 6 months?<br><br>2. Does this facility have			

formula, breast pumps, milk-storage facilities, pasteurizers, milk banks, if possible, nasogastric tubes, syringe drivers, intravenous fluids and tubing).	5. graduated cup to measure expressed milk?<br><br>6. small cup for feeding expressed breast milk?<br><br>7. Chair/tool for the patient<br><br>8. IV fluid<br><br>9. Endotracheal tube		recorded evidence of KMC (prolonged STSC, EBF/feeding support, growth monitoring (weight)? If yes, was it done in the past 6 months?<br><br>3. If baby not breastfeeding, teach her to express the milk and feed with a clean cup.			
4. Health care staff in the health facility who work with pregnant and postpartum women and newborns receive in service training		1.How often have they been trained over the past year?<br><br>2. Does the training sessions cover Essential newborn care?	1.Have you received in-service training, and/ or refresher in the care for the low birthweight or preterm newborns in the last 12 months?	List the items in the resuscitation area:<br>1.Clean towels- at least 2<br><br>2. Radiant warmers	What was/were done for the baby(ies) with preterm birth complications?	

and regular refresher sessions in appropriate care of preterm and low birth weight babies at least once every 12 months.		3. Facility specific policies on KMC for babies?	2. Does this facility have recorded evidence of KMC (prolonged STSC, EBF/feeding support, growth monitoring (weight)? If yes, was it done in the past 6 months?	Immediate care of the newborn:<br>3. Baby dried immediately and thoroughly;<br><br>4. Baby placed skin to skin contact with the mother immediately after birth<br><br>5. Time baby was first put to breast<br><br>6. Baby's temperature taken after birth		
Output/process measures	F1.PSFR	F2.MI	F3.SIV	F4.OPCI	F5.CMRR	F6.WEICPC
1.The proportion of all low-birth-	1.Does the facility provide	Facility specific policies on KMC for babies?	1.Have you received in-service training,	Gestational age of the pregnancy	1.Are birth weights	

weight newborns born in the health facility with a birth weight ≤2000g who received near-continuous Kangaroo Mother Care in the first week of life.	services for KMC ward/beds<br><br>2. Does the facility have functioning weighing machine?		and/ or refresher in the care for the low birthweight or preterm newborns in the last 12 months?<br><br>2. Does this facility have dedicated space for Kangaroo Mother Care? if yes, was it done in the past 6 months?<br><br>3. Does this facility have recorded evidence of KMC (prolonged STSC, EBF/feeding support, growth monitoring (weight)? If yes, was it done in	What was the weight of the baby?	recorded for all babies?<br><br>2.What was the baby weight for baby1/baby2/baby3?<br><br>3. What was/were done for the baby(ies) with preterm birth complications?	

			the past 6 months?			
2. The proportion of all unstable low-birth-weight newborns weighing ⩽ 2000 g who cannot receive kangaroo mother care in the health facility who were cared for in a thermo-neutral environment, either under radiant warmers or in incubators, as appropriate	1. Does the facility provide services on NICU?<br><br>2. Does the facility have functioning incubators and radiant warmers?	Do training sessions cover Essential newborn care?	1.Facility provide comprehensive newborn intensive care unit and was is done/provided in the past 6 months?<br><br>2. Have you received refresher/in-service training in the past 12 months on Care for sick newborns?		1.Are birth weights recorded for all babies?<br>2.What was the baby weight for baby1/baby2/baby3?<br><br>3. What was/were done for the baby(ies) with preterm birth complications?	
3. The proportion of all low-birth-weight newborns born in the health	1. Does the facility have functional graduated cup to measure	1.Does the training session cover Essential newborn care?	1.Have you received refresher/in-service training in the past 12 months on	Was the woman counselled on breastfeeding?	1.What was the baby weight for baby1/baby2/baby3?	1.Are you satisfied with the information you received on breastfeeding?

facility whose mothers received additional support to establish breastfeeding.	expressed breast milk?<br><br>2. Does the facility have functional have functional small cup for feeding expressed breast milk?	2. Facility specific management policies on i. Baby friendly, ii. Early Initiation of breastfeeding, iii. Exclusive breastfeeding or breastmilk feeding	Breastfeeding counselling and lactation management?<br><br>Vignettes:<br>2. Ensure breastfeeding is established and provide support if necessary<br><br>3. Watch her breastfeed her baby and teach her good positioning and attachment<br><br>4. Examine the baby's mouth to ensure there are no anatomical deformities<br><br>5. If baby not breastfeeding, teach her to		2. Was the woman counselled on breastfeeding?	2. Do you feel you are competent enough to be able to breast feed your baby on your own?

			express the milk and feed with a clean cup			
Outcome measures	F1.PSFR	F2.MI	F3.SIV	F4.OPCI	F5.CMRR	F6.WEICPC
1.The proportion of all preterm babies (< 28 weeks, 28–32 weeks and 32–37 weeks of gestational age) born in the health facility who died within the first 7 days of life	1.Does this facility routinely conduct audit for early neonatal deaths?<br><br>2. Does this facility routinely conduct audit for stillbirths?	Facility specific management policies on systematic mortality audits		Gestational age of the pregnancy?	1. Number of completed weeks of gestation at delivery?<br><br>2. Delivery outcome of the newborn?<br><br>3. if stillbirth/early neonatal death, what was the primary underlying cause of death?<br><br>Date of delivery?	

					4. Time of delivery?<br><br>5. Date and time of discharge?	
2. The proportion of all low-birth-weight newborns born in the health facility who were exclusively fed on their mother's milk during their stay in the health facility	1. Does the facility have functional graduated cup to measure expressed breast milk?<br><br>2. Does the facility have functional have functional small cup for feeding expressed breast milk?	1.Facility specific management policies on i. Baby friendly, ii. Early Initiation of breastfeeding, iii. Exclusive breastfeeding or breastmilk feeding	1.Have you received refresher/in-service training in the past 12 months on Breastfeeding counselling and lactation management?<br><br>Vignettes:<br>2. Ensure breastfeeding is established and provide support if necessary<br><br>3 If baby not breastfeeding, teach her to express the milk	Was the woman counselled on breastfeeding?	Was the woman counselled on breastfeeding?	1.Are you satisfied with the information you received on breastfeeding?<br><br>2. Do you feel you are competent enough to be able to breast feed your baby on your own?

			and feed with a clean cup<br><br>4. Educate her and encourage her to practice exclusive breastfeeding for the 1st 6 months of the baby's life			
3. The proportion of all live preterm babies born in the health facility who had severe neonatal morbidity (respiratory distress syndrome, intraventricular haemorrhage, necrotizing enterocolitis).	Does this facility have a separate paediatric ward?	1.Are there charts to show coverage and quality?<br><br>2. Is there data on numbers of sick newborns in this facility in a year?<br><br>3. How recent is the latest chart; record month and year	1.Does this facility operate a static child welfare clinic?<br><br>2. Does this facility provide comprehensive Newborn Intensive Care Unit and was it done/ provided in the past 6 months?		If live birth with complications(whether alive or dead), what complication did the baby (ies) have?	

4. The proportion of low-birth-weight baby deaths in the facility attributed to possible serious bacterial infection or sepsis	1.Does this facility routinely conduct audit for early neonatal deaths?<br><br>2. Does this facility routinely conduct audit for stillbirths?<br><br>3. does this facility have a separate paediatric ward?	1.Does this facility conduct systematic mortality audits?<br><br>2. How does the facility monitor its performance?	1.Does this facility operate a static child welfare clinic?<br><br>2. Does this facility provide comprehensive Newborn Intensive Care Unit and was it done/ provided in the past 6 months?		If stillbirth/early neonatal death, what was the primary underlying cause of death?	
Quality statement 1.7a						
Women with or at risk for infections during labour, childbirth or the early postnatal period promptly receive appropriate interventions, according to WHO guidelines.						
Input measures	F1.PSFR	F2.MI	F3.SIV	F4.OPCI	F5.CMRR	F6.WEICPC
1.The health facility has supplies of oral and injectable first- and	Health facility availability of essential drugs (IV antibiotics, Magnesium		Are injectable antibiotics administration provided in this facility and were	1. Did the health worker set an IV line on woman?	1.Did the woman receive any antibiotic?	Were you given any of the IV fluid, medicine to take,

second-line antibiotics (ampicillin or penicillin and gentamicin, clindamycin, cephalosporin and metronidazole) available in sufficient quantities at all times for the expected case load	Sulphate (MgS04), Diazepam, Oxytocic (syntometrine/Ergot)), Dexamethasone /Betamethasone (parenteral), Hydralazine/SL Nifedipine, Ampicillin/Penicillin for IV, Gentamicin for IV, Cephalosporins)		they provided/done in the past 6 months?	2. Was the labour augmented at any time?<br><br>3. Did woman have an IV line access?<br><br>4. Is there oxytocin in the delivery tray?<br><br>Epidural given for the delivery?	2. Did the baby receive any antibiotic?<br><br>What antibiotics was given? (Ampicillin/Penicillin, Gentamicin, Cephalosporins)	Injection (IM), pain relief?
2.The health facility has written, up-to-date clinical protocols for treatment of women with, or at risk for, infections during labour, childbirth and the early	1.Is there a protocol for infection control?<br><br>2. Does the facility provide services for incinerator?<br><br>3. Does the facility have	1. What are the main policies of this facility regarding maternity and newborn care? collect any points.<br><br>2. Facility specific management	1. Is there an IPC policy available in your facility?<br><br>2. Is there an IPC focal person?<br><br>3. How often do you wash your hands before	1.Observe whether skilled attendants wear PPE?<br><br>2. Are staff observed to be using PPE correctly?<br><br>3. Are puncture – proof sharps	If maternal death, was infection the main underlying cause of death?	1.Did you have soap and water to wash your hands after visiting the toilet?<br><br>2. Overall, how satisfied are you with the hygiene

postnatal period in the childbirth and postnatal care areas of the maternity unit that are consistent with WHO guidelines	functional sterile gloves?	policies on Infectious control and notification system<br><br>3. At what level do you have a focal person for infection prevention and control (select as appropriate)	and after examining a patient?<br><br>4. Are you able to mention the "five moments" of hand hygiene?<br><br>5. Do you use Chlorine to decontaminate used equipment?	containers located in all clinical areas?<br><br>4. Are sharp containers no more than ¾ full?<br><br>5. Is there a concrete -lined sharp pit or incinerator for sharps disposal?		standards at the health facility>
3.Health care staff in the health facility who deal with pregnant and postpartum women receive in-service training and regular refresher sessions in the recognition and	Is there a protocol for infection control?	1. Facility specific management policies on Infectious control and notification system<br><br>2. At what level do you have a	1.Have you received in-service training, and/ or refresher in the management of puerperal sepsis in the last 12 months?<br>2. Have you received in-service training, and/ or	1.Observe whether skilled attendants wear PPE?<br><br>2. Are staff observed to be using PPE correctly?<br><br>3. Are puncture – proof sharps containers	If maternal death, was infection the main underlying cause of death?	1.Did you have soap and water to wash your hands after visiting the toilet?<br><br>2. Overall, how satisfied are you with the hygiene standards at the health facility>

management of maternal peri-partum infections at least once every 12 months		focal person for infection prevention and control (select as appropriate)	refresher in the management of abortion complication in the last 12 months?<br><br>3. . Is there an IPC policy available in your facility?	located in all clinical areas?<br><br>4. Are sharp containers no more than ¾ full?		
Output/process measures	F1.PSFR	F2.MI	F3.SIV	F4.OPCI	F5.CMRR	F6.WEICPC
1.The proportion of all women who underwent caesarean section in the health facility who received prophylactic antibiotics before caesarean section	1. Does the facility have a theatre?<br><br>2. Essential drugs: IV antibiotics		Are injectable antibiotics administration provided in this facility and were they provided/done in the past 6 months?		1.Did the woman receive any antibiotic?<br><br>2. What antibiotics was given? (Ampicillin/Penicillin, Gentamicin, Cephalosporins)<br><br>3. was prophylactic	Were you given any of the IV fluid, medicine to take, Injection (IM), pain relief?

					antibiotics administered after surgery?<br><br>4. what was the indication for Caesarean Section	
2. The proportion of all women who gave birth in the health facility with preterm pre-labour rupture of membranes who received antibiotics.	Essential drugs: IV antibiotics		Are injectable antibiotics administration provided in this facility and were they provided/done in the past 6 months?		1.What was done with a woman with obstetric complication?<br>2. Did the woman receive antibiotic?<br>If yes, what antibiotic was given?	Were you given any of the IV fluid, medicine to take, Injection (IM)?
3. The proportion of all women in the health facility with third- or fourth-degree perineal tears who received antibiotics	Essential drugs: IV antibiotics		Are injectable antibiotics administration provided in this facility and were they provided/done in the past 6 months?		1.What was done with a woman with obstetric complication (obstructed labour, pre-eclampsia/ecla mpsia,	Were you given any of the IV fluid, medicine to take, injection (IM)?

					postpartum haemorrhage, third degree tear, prolapsed cord, malpresentation e.g., breech, brow, transverse lie etc, shoulder dystocia, fever >38∝?<br>2. Did the woman receive antibiotic?<br>If yes, what antibiotic was given?	
4. The proportion of all birthing or postpartum women in the health facility with signs of infection who received injectable antibiotics	Essential drugs: IV antibiotics		Are injectable antibiotics administration provided in this facility and were they provided/done in the past 6 months?		1.What was done with a woman with obstetric complication (fever >38∝, etc)?<br>2. Did the woman receive antibiotic?	Were you given any of the IV fluid, medicine to take, injection (IM)

					If yes, what antibiotic was given?	
5.The proportion of all women who gave birth in the health facility who had temperature of 38℃ or other signs of infection foul-smelling or purulent lochia) after childbirth.	1.Thermometer-digital/mercury column<br><br>2. Is there a protocol for infection control	1. Does the training session cover management of birth complications?<br><br>2. Facility specific management policies on infection control and notification system	Is there an IPC policy available in your facility?	1.Did staff wash their hands before and after examining patients?<br><br>2. Are healthcare workers complaint with the hand hygiene "five moments"	1.Did the woman have foul-smelling discharge from the vagina as a complication during pregnancy?<br><br>2. What was done with the woman with foul-smelling discharge from the vagina as a complication during pregnancy?<br><br>3. What was done with a woman with obstetric complication (fever >38∝, etc)	Did you have a temperature or fever complications after the birth?

Outcome measures	F1.PSFR	F2.MI	F3.SIV	F4.OPCI	F5.CMRR	F6.WEICPC
1.The proportion of all women who underwent caesarean section in the health facility who had severe systemic infection or sepsis after the caesarean section.		Facility specific management policies on i. systematic “near miss” audits, ii. Infectious control and notification system	Is there an IPC policy available in your facility?		1.Type of delivery?<br><br>2. Was the surgery complicated by wound infection or burst abdomen?<br><br>3. what prophylactic antibiotics administered after the surgery?<br><br>4. If maternal death, was Infection the main underlying cause of deaths?	

2. The proportion of all women who gave birth in the health facility who had severe systemic infection or sepsis in the postpartum period		Facility specific management policies on i. systematic "near miss" audits, ii. Infectious control and notification system	Is there an IPC policy available in your facility?		1.Was the surgery complicated by wound infection or burst abdomen?<br><br>2. Which of the obstetric complication(s) did the woman have fever >38∝?	
Quality statement 1.7b						
Newborns with suspected or risk factors for infection are promptly given antibiotic treatment, according to WHO guidelines.						
Input measures	F1.PSFR	F2.MI	F3.SIV	F4.OPCI	F5.CMRR	F6.WEICPC
1.The health facility has supplies of injectable antibiotics(at least first-and second-line antibiotics for neonatal sepsis and meningitis)	Health facility availability of essential drugs (IV antibiotics, Magnesium Sulphate (MgS04), Diazepam, Oxytocic (syntometrine/E		Health facility has Injectable antibiotics (Ampicillin/Penicillin and Gentamicin) for management of suspected sepsis, and was done/provided		1.If live birth with complications (whether alive or dead) which complication did the baby(ies) have?	

available in sufficient quantities at all times for the expected case load	rgot)), Dexamethasone /Betamethasone (parenteral), Ampicillin/Penicillin for IV, Gentamicin for IV, Cephalosporins)		in the past 6 months?		2. What was done with the baby(ies) with the above complications?<br><br>3. Did the newborn(s) receive any antibiotic?<br><br>What antibiotics was given? (Ampicillin/Penicillin, Gentamicin, cephalosporins?)	
2. The health facility has a written, up-to-date clinical protocol for early diagnosis and management of neonatal infection in the	Is there a protocol for infection control?	1. Does the facility have a policy on infectious control and notification system?<br><br>3. do you know about the hand	1.Is there an IPC policy available in your facility?<br><br>2. Is there IPC focal person?<br><br>3. Are you able to mention the "five moments"	1.Did staff wash their hands before and after examining patients?<br><br>2. Are healthcare workers complaint with	1.If live birth with complications (whether alive or dead) which complication did the baby(ies) have?	

childbirth areas of the maternity unit that is consistent with WHO guidelines		hygiene "5 moments"?	of hand hygiene?<br><br>4. Have you received in-service training, and/ or refresher on neonatal sepsis in the last 12 months?	the hand hygiene "five moments"	2. What was done with the baby(ies) with the above complications?	
3. Health care staff in the health facility who care for pregnant and postpartum women and newborns receive in-service training and regular refresher sessions in the recognition and management of suspected newborn		1.Are there additional refresher training for the staff?<br><br>2. Are there any training over the past year?<br><br>3. How often have then been trained over the past year?<br><br>4. Does the training session	1.Have you received in-service training, and/ or refresher in the management of sick newborn in the last 12 months.<br>2. Have you received in-service training, and/ or refresher on neonatal sepsis in the last 12 months?		1.If live birth with complications (whether alive or dead) which complication did the baby(ies) have?<br><br>2. What was done with the baby(ies) with the above complications?	

infections at least once every 12 months		cover Essential Newborn care?				
4.Health care staff in the health facility know the signs of newborn sepsis and how to treat it, according to WHO guidelines	Is there a protocol for infection control?	1. Does the facility have a policy on infectious control and notification system?<br><br>3. do you know about the hand hygiene "5 moments"?	1.Have you received in-service training, and/ or refresher in the management of sick newborn in the last 12 months?<br><br>2. Have you received in-service training, and/ or refresher on neonatal sepsis in the last 12 months?		1.If live birth with complications (whether alive or dead) which complication did the baby(ies) have?<br><br>2. What was done with the baby(ies) with the above complications?	
Output/process measures	F1.PSFR	F2.MI	F3.SIV	F4.OPCI	F5.CMRR	F6.WEICPC
1.The proportion of all newborns in the health facility	1. Essential drugs: IV antibiotics?	Does the facility have a policy on infectious control and	1.Does this facility have injectable antibiotics		1.If live birth with complications (whether alive	

with signs of infection who received injectable antibiotics	2. Is there a protocol for infection control?	notification system?	(Ampicillin/Penicillin and Gentamicin) for management of suspected sepsis, and was it done in the past 6 months?<br><br>2. Have you received in-service training, and/ or refresher on neonatal sepsis in the last 12 months?		or dead) which complication did the baby(ies) have?<br><br>2. What was done with the baby(ies) with the above complications?<br><br>3. Did the newborn(s) receive any antibiotic?<br><br>4. If yes, what antibiotics was given?(Ampicillin/Penicillin, Gentamicin, Cephalosporins)	
2. The proportion of all newborns of mothers with signs of infection in the	Essential drugs: IV antibiotics?	Does the facility have a policy on infectious control and notification system?	Does this facility have injectable antibiotics (Ampicillin/Penicillin and		1.Did the newborn(s) receive any antibiotic?	

health facility who received injectable antibiotics			Gentamicin) for management of suspected sepsis, and was it done in the past 6 months?		2. If yes, what antibiotics was given?(Ampicillin/Penicillin, Gentamicin, Cephalosporins)<br><br>3. If the baby(ies) received IV fluid, why was it given?	
Outcome measures	F1.PSFR	F2.MI	F3.SIV	F4.OPCI	F5.CMRR	F6.WEICPC
1.The proportion of newborns treated for sepsis in the health facility who died (case fatality rate)	1.Does the facility routinely conduct audit for early neonatal deaths?<br><br>2. Does the facility routinely conduct audit for stillbirths?	Facility specific management policies on systematic mortality audits	Does this facility have injectable antibiotics (Ampicillin/Penicillin and Gentamicin) for management of suspected sepsis, and was it done in the past 6 months?		Did the newborn(s) receive any antibiotic?<br>If yes, what antibiotics was given?(Ampicillin/Penicillin, Gentamicin, Cephalosporins)	

2.The proportion of neonatal deaths in the health facility that were due to sepsis	1.Does the facility routinely conduct audit for early neonatal deaths?<br><br>2. Does the facility routinely conduct audit for stillbirths?	Facility specific management policies on systematic mortality audits	Does this facility have injectable antibiotics (Ampicillin/Penicillin and Gentamicin) for management of suspected sepsis, and was it done in the past 6 months?		1.If stillbirth/early neonatal death, what was the primary underlying cause of death?	
3.The proportion of all severe neonatal morbidity in the health facility that was due to neonatal sepsis.		Facility specific management policies on systematic "near miss" audits	Does this facility have injectable antibiotics (Ampicillin/Penicillin and Gentamicin) for management of suspected sepsis, and was it done in the past 6 months?		If stillbirth/early neonatal death, was Preterm related, Asphyxia and birth trauma, Infection, Congenital malformation, accidents/injuries, other (specify), unknown, no	

					information, the primary underlying cause of death?	
Quality statement 1.8						
All women and newborns who received care that includes standard precautions for preventing hospital-acquired infections.						
Input measures	F1.PSFR	F2.MI	F3.SIV	F4.OPCI	F5.CMRR	F6.WEICPC
1.The health facility has a reliable water source on site and soap and towels (preferably disposable) or alcohol-based hand rub for hand hygiene	1. what is used for handwashing on the maternity toilet?<br><br>2. how do users of the maternity toilet clean their hands after handwashing?<br><br>3. What is the main source of water for cleaning in the facility?<br><br>4. what is the main source for		How often do you wash your hands before and after examining a patient?	1.Did staff wash their hands before and after examining patients?<br><br>2. How do staff dry their hands?<br><br>3. Are functioning sinks with clean, running water available in all clinical rooms/wards/treatment areas for handwashing (one per ward)?		Did you have soap and water to wash your hands after visiting the toilet?<br><br>How did you dry your hands after washing?

	drinking water for the facility?<br><br>5. is there a system for checking the quality of drinking water in this facility?			4. Are sinks equipped with bar/liquid soap in clinical areas?		
2. The health facility ensures safe handling, storage and final disposal of infectious waste	1.Are there waster bins in the maternity and labour ward?<br><br>2. Are there waste bins in the maternity and labour ward colour coded and labelled?<br><br>3. Are puncture -proof containers provided in each clinical area?	Facility specific management policies on infectious control and notification system	1.Is there an IPC policy available in your facility?<br><br>2. Is there an IPC focal person?	1. Are puncture – proof sharps containers located in all clinical areas?<br><br>2. Are sharp containers no more that ¾ full?<br><br>3. Is there a concrete line sharp pit or incinerator for sharps disposal?<br><br>4. Is there a well ventilated,		

	4. Is the sharp container in the labour ward less than three-quarter full? (check)<br><br>5. Is there a pit for disposal of placentae?<br><br>6. Is the placentae pit well ventilated?<br><br>7. Does the facility provide incinerator services?<br><br>8. Is there a protocol for infection control?			maintained and protected placenta pit (fenced)?		
3. The health facility ensures safe handling, storage	1.Are there waster bins in the maternity	Facility specific management policies on infectious	1.Is there an IPC policy available in your facility?	1. Are puncture – proof sharps containers		

(puncture resistant) and final disposal of sharps waste	and labour ward?<br><br>2. Are there waste bins in the maternity and labour ward colour coded and labelled?<br><br>3. Are puncture -proof containers provided in each clinical area?<br><br>4. Is the sharp container in the labour ward less than three-quarter full? (check)<br><br>5. Is there a pit for disposal of placentae?	control and notification system	2. Is there an IPC focal person?	located in all clinical areas?<br><br>2. Are sharp containers no more that ¾ full?<br><br>3. Is there a concrete line sharp pit or incinerator for sharps disposal?<br><br>4. Is there a well ventilated, maintained and protected placenta pit (fenced)?		

	6. Is the placentae pit well ventilated?<br><br>7. does the facility provide incinerator services?<br><br>8. Is there a protocol for infection control?					
4. The health facility has appropriate sterilizing facilities and disinfectants for instruments	1.Does the facility provide services on Autoclave for sterilization?<br><br>2. Is there a protocol for infection control?	Facility specific management policies on infectious control and notification system	Is there an IPC policy available in your facility?			
5. The health facility has a functioning incinerator or other	Does the facility provide incinerator services?	Facility specific management policies on infectious control and	Is there an IPC policy available in your facility?	1.Is there a concrete-lined sharp pit or incinerator for sharps disposal?		

appropriate method for treatment of infectious waste and used instruments		notification system		2.Is there a well ventilated, maintained and protected placenta pit (fenced)?		
6. The health facility has written, up-to-date guidelines for standard infection control and precautions for transmission	Is there a protocol for infection control?	Facility specific management policies on infectious control and notification system	Is there an IPC policy available in your facility?	1.Did staff wear gloves when handling medical waste?<br><br>2. Was the delivery unit cleaned after the delivery?<br><br>3. Observe whether skilled attendants wear PPE (tick)<br><br>4. Are staff observed to be using PPE correctly?		
7. Health care staff in the childbirth and	Is there a protocol for	1.What additional training is	1.Have you received in-service training,			

neonatal areas of the maternity unit receive training in standard infection control and precautions for transmission at least once every 12 months	infection control?	provided to workers posted in maternity and children's ward?<br><br>2. Are there additional refresher training for the staff?<br><br>3. have there been training over the past year?	and/ or refresher on neonatal sepsis in the last 12 months?<br><br>2. Have you received in-service training, and/ or refresher in the management of puerperal sepsis in the last 12 months?			
Output/process measures	F1.PSFR	F2.MI	F3.SIV	F4.OPCI	F5.CMRR	F6.WEICPC
1.The percentage of health care staff in the health facility who clean their hands correctly as per the WHO "5 moments for	1.Does the facility have functioning hand wash station?<br><br>2. Is there a protocol for	1.Do you know about the hand hygiene "5 moments"?<br><br>2. Is hand hygiene moments part of	1.How often do you wash your hands before and after examining a patient?<br><br>2. Are you able to mention the	Are healthcare workers compliant with the hand hygiene "five moments"?		Did you have soap and water to wash your hands after visiting the toilet?

hand hygiene" audit tool	infection control?	routine practice in the facility?	"five moments" of hand hygiene?			
2. The proportion of newborns with suspected severe bacterial infection who received appropriate antibiotic therapy	1.Did the newborn(s) receive any antibiotic?<br><br>2. If yes, what antibiotics was given? (Ampicillin/Penicillin, Gentamicin, Cephalosporins)<br><br>3. If the baby(ies) received IV fluid, why was it given.		1.Is management of suspected sepsis with injectable antibiotics (Ampicillin/Penicillin and Gentamicin) provided in this facility and was it done in the past 6 months?<br><br>2. Is IV fluid administration provided in this facility and was it done in the past 6 months?		1.What was/were done with baby(ies) with complications?<br><br>2. Did the newborn(s) receive any antibiotic?<br><br>3. What antibiotics was given? (Ampicillin/Penicillin, Gentamicin, Cephalosporin?)	
3.Safe management of health care waste, from the point of generation to	1.Are there waste bins in maternity and labour ward colour coded and labelled?	Facility specific management policies on infectious control and	Is there an IPC policy available in your facility?<br><br>Is there an IPC focal person?	1.Is there a concrete-lined sharp pit or incinerator for sharps disposal?		

the point of disposal	2. Are puncture proof containers provided in each clinical area?<br><br>3. Is there a pit for disposal of placentae?<br><br>4. Does the facility provide and/or has incinerator?<br><br>5. Water proof aprons in delivery suite?	notification system?<br><br>At what level do you have focal persons for infection prevention and control?		2. Is there a well ventilated, maintained and protected placenta pit (fenced)?<br>3. Did staff wear gloves when handling medical waste?<br><br>4. Was the delivery unit cleaned after the delivery?		
4. The percentage of staff members in the health facility who meet biosafety standards when administering parenteral drugs.	Is there a protocol for infection control?	Does the training sessions cover quality of care?<br><br>Facility specific management policies on infectious control and	Is there an IPC policy available in your facility?	1.Are healthcare workers complaint with the hand hygiene “five moments”?<br><br>2. Served using PPE correctly?	Was the woman given parenteral oxytocin?	

		notification system?				
Outcome measures	F1.PSFR	F2.MI	F3.SIV	F4.OPCI	F5.CMRR	F6.WEICPC
1.The proportion of all women who gave birth in the health facility who had a severe systemic infection or sepsis in the postnatal period, including at readmission after delivery in the facility	1.Does this facility have a separate maternity ward?<br><br>2. Does this facility routinely conduct audit for maternal deaths?	Does your facility has a quality improvement programme, which includes quality improvement teams, capturing and use of data, monitoring and evaluation.	Have you received refresher/in-service training in the past 12 months on management of puerperal sepsis?		Was the surgery complicated by wound infection or burst abdomen?	
2.The proportion of all women who gave birth in the health facility who had proven hospital-	1.Does this facility have a separate maternity ward?<br><br>2. Does this facility routinely	Does your facility has a quality improvement programme, which includes quality improvement	Have you received refresher/in-service training in the past 12 months on management of		1.Was the surgery complicated by wound infection or burst abdomen?	

acquired infections	conduct audit for maternal deaths?	teams, capturing and use of data, monitoring and evaluation.	puerperal sepsis?		2.If maternal deaths, was Infection, or other, specify the main underlying cause?	
3.The proportion of all neonates born in the health facility who had hospital-acquired infections	1.Does this facility have a separate paediatric ward?<br><br>2. Does this facility routinely conduct audit for early neonatal deaths?	Does your facility has a quality improvement programme, which includes quality improvement teams, capturing and use of data, monitoring and evaluation.			If stillbirth/early neonatal deaths, what was the primary underlying cause of death?	
Quality statement 1.9						
No woman or newborn is subjected to unnecessary or harmful practices during labour, childbirth and the early postnatal period.						
Input measures	F1.PSFR	F2.MI	F3.SIV	F4.OPCI	F5.CMRR	F6.WEICPC
1.The health facility has written, up-to-date guidance		1. Facility specific management policies on	1.Is there a policy and procedure for addressing			Were you subjected to any physical abuse, verbal abuse,

on harmful practices and unnecessary interventions during labour, childbirth and the early postnatal period		Respectful and dignified care for all<br><br>3. Is there a process for identifying abuse of women and/ or children who seek care in your facility?<br><br>4. If yes, do you have a process of reporting these cases? Are women encouraged to report such abuses from health professionals to managers?<br><br>5. If yes, can you show the most recent report of abuse?	patient concerns in this facility?<br><br>2. Does your facility have clear policies on rights of the patient?<br><br>3. Have you received training on how to treat childbearing women with compassion and dignity (customer care)?<br><br>4. Is there a process for identifying and reporting abuse?			sexual abuse, other, specify...?

2.The health facility does not display infant formula or bottles and teats, including on posters or placards	Does the facility have functioning graduated cup to measure expressed breast milk, small cup for feeding expressed breast milk?	1.Facility specific management policies on Baby Friendly?<br><br>2. Facility specific management policies on Early Initiation of breastfeeding?<br><br>3. Facility specific management policies on Exclusive breastfeeding or breastmilk feeding	1.Have you received refresher/in-service training in the past 12 months on breastfeeding counselling and lactation management?<br><br>Vignettes:<br>2. Ensure breastfeeding is established and provide support if necessary.<br><br>3. Watch her breastfeed her baby and teach her good positioning and attachment<br><br>4. If the baby not breastfeeding,			

			teach her to express the milk and feed with a clean cup.<br><br>5. Encourage infant formula only if EBM is not possible and mother can afford<br><br>6. Educate her and encourage her to practice exclusive breastfeeding for the 1st 6 months of the baby's life.			
3.The health facility does not give newborns food or drink other than breast milk, unless medically indicated, and	1. Does the facility has functioning graduated cup to measure expressed breast milk?	1.Facility specific management policies on Baby Friendly?<br><br>2. Facility specific	1.Have you received refresher/in-service training in the past 12 months on breastfeeding counselling and	Was woman counselled on breastfeeding?	Was the woman counselled on breastfeeding?	1.Are you satisfied with the information you received on breastfeeding?<br><br>2. Do you feel competent

does not give pacifiers (also called “dummies” or “soothers”) to breastfeeding babies.	2.Does the facility has functioning small cup for feeding expressed breast milk?	management policies on Early Initiation of breastfeeding?<br><br>3. Facility specific management policies on Exclusive breastfeeding or breastmilk feeding	lactation management?<br><br>Vignettes:<br>2. Ensure breastfeeding is established and provide support if necessary.<br><br>3. Watch her breastfeed her baby and teach her good positioning and attachment<br><br>4. If the baby not breastfeeding, teach her to express the milk and feed with a clean cup.<br><br>5. Encourage infant formula only if EBM is			enough to be able to breastfeed your baby on your own?

			not possible and mother can afford<br><br>6. Educate her and encourage her to practice exclusive breastfeeding for the 1st 6 months of the baby's life.			
4.Health care staff in the facility receive in-service training and regular refresher sessions on harmful practices and unnecessary interventions at least once every 12 months.		1.Facility specific management policies on Respectful and dignified care for all<br><br>2. Is there a process for identifying abuse of women and/ or children who seek care in your facility?	1.Does your facility have clear policies on rights of the patient?<br><br>2. Have you received training on how to treat childbearing women with compassion and dignity (customer care)?			Were you subjected to any physical abuse, verbal abuse, sexual abuse, other, specify…?

		3. If yes, do you have a process of reporting these cases?<br><br>4. Are women encouraged to report such abuses from health professionals to managers?<br><br>5. If yes, can you show the most recent report of abuse?	3. Is there a process for identifying and reporting abuse?			
5.The health facility does not promote infant formula on the wards, and samples are not distributed to mothers or staff.	1. Does the facility has functioning graduated cup to measure expressed breast milk?<br><br>2.Does the facility has functioning	1.Facility specific management policies on Baby Friendly?<br><br>2. Facility specific management policies on Early	1.Have you received refresher/in-service training in the past 12 months on breastfeeding counselling and lactation management?	Was woman counselled on breastfeeding?	Was the woman counselled on breastfeeding?	1.Are you satisfied with the information you received on breastfeeding?<br><br>2. Do you feel competent enough to be able to breastfeed your

	small cup for feeding expressed breast milk?	Initiation of breastfeeding?<br><br>3. Facility specific management policies on Exclusive breastfeeding or breastmilk feeding	Vignettes:<br>2. Ensure breastfeeding is established and provide support if necessary.<br><br>3. If the baby not breastfeeding, teach her to express the milk and feed with a clean cup.<br><br>4. Encourage infant formula only if EBM is not possible and mother can afford<br><br>5. Educate her and encourage her to practice exclusive breastfeeding for the 1st 6			baby on your own?

			months of the baby's life.			
6.Health-care staff in the health facility receive monthly supportive supervision and mentoring on harmful practices and unnecessary interventions.		1.Do you receive external supervisory visits to this facility?<br><br>2.If yes, how often do you receive these external visits?<br><br>3. Does your facility have a quality improvement programme, which include quality improvement mentoring/coac hing?	1. Have you received training on how to treat childbearing women with compassion and dignity (customer care)?<br><br>2. Is there a process for identifying and reporting abuse?			Were you subjected to any physical abuse, verbal abuse, sexual abuse, other, specify…?
Output/process measures	F1.PSFR	F2.MI	F3.SIV	F4.OPCI	F5.CMRR	F6.WEICPC
1.The proportion of all	1.Does the facility have			1.List contents of the delivery	Type of delivery (spontaneous	Type of delivery (spontaneous

uncomplicated, spontaneous vaginal births in the health facility in which an episiotomy was performed.	functioning delivery forceps?<br><br>2. Sterile gloves<br><br>3. Water proof aprons in delivery suite<br><br>4. Delivery kit			tray (Suture and needle, sharp scissors, oxytocin, cord clamp, lean towels x2)<br><br>2. Episiotomy done?	vaginal delivery, assisted vaginal delivery, a planned caesarean section delivery, an emergency caesarean section delivery)	vaginal delivery, assisted with vacuum instruments, Caesarean Section).
2.The proportion of women undergoing caesarean section in the health facility according to Robson classification groups.	1.Does the facility provide services for Theatre?<br><br>2. Level of the facility (hospital, health centre, tertiary facility)		1.Is Caesarean Section as a signal EmOC function provided in this facility, and was it done in the past 6 months?<br><br>Vignettes:<br>2. Prepare for Caesarean Section;<br><br>3. Refer to hospital where Caesarean	Plan for delivery communicated to mother (SVD/Assisted vaginal delivery/C/S)	Type of delivery (spontaneous vaginal delivery, assisted vaginal delivery, a planned caesarean section delivery, an emergency caesarean section delivery)	Type of delivery (spontaneous vaginal delivery, assisted with vacuum instruments, Caesarean Section).

			Section can be done			
3.The proportion of all women who gave birth in the health facility who received augmentation of labour with no indication of delay in progress of labour.				Was the labour augmented at any time?	1.Was labour augmented?<br>2.If augmentation was done, when was it started? (i. on or to the right of the action line; ii. Between alert and action lines; iii. On or to the right of the action line; iv. Not recorded) ,	
6.The proportion of all babies born through clear amniotic fluid in the health facility who received routine suctioning.	Does the facility have functioning sucker machine?		1. Have you received refresher/in-service training in the past 12 months on Early Essential Newborn Care?<br><br>Is Resuscitation by cleaning the	Buld syringe for aspiration of fluids	Was the liquor meconium stained?	

			airway provided in this facility and was it done in the past 6 months?			
Standard 2: The health information system enables use of data to ensure early, appropriate action to improve the care of every woman and newborn.						
Quality statement 2.1						
Every woman and newborn has a complete, accurate, standardized medical record during labour, childbirth and the early postnatal period.						
Input measures	F1.PSFR	F2.MI	F3.SIV	F4.OPCI	F5.CMRR	F6.WEICPC
1.The health facility has registers, data collection forms, clinical and observation charts in place at all time for routine recording and monitoring of all care processes for women and newborns.	Does this facility admit patient over night?<br><br>Does the facility have a functioning emergency patient register?<br><br>Does this facility have a separate maternity ward?	1.How does the facility monitor its performance?<br><br>2.What does the facility do with data collected on patient care?<br><br>3.Is there data on numbers of deliveries in this facility in a year?	Have you received refresher/in-service training in the past 12 months on partograph use?	1.History taken?<br>2. Woman's pregnancy records reviewed (if available)<br><br>3. Record time of delivery?	1.Was partograph used to monitor labour?<br><br>2. Is/are the birth weights recorded for all babies?<br><br>3. How many times was the woman's temperature checked and recorded	

	Does this facility have a separate paediatric ward?	4. Are there charts to show coverage and quality?<br><br>5. Is there data on numbers of sick newborns in this facility in a year?<br><br>6. Does the facility have quality improvement programme which includes capturing and use of data?			between first exam and delivery?<br><br>4. How many times was the blood pressure checked and recorded between first exam and delivery?<br><br>5. How many times was the mother's pulse checked and recorded between first exam and delivery?<br><br>6. Was the fetal heart rate recorded?	
2.The health facility has a birth and death		1. Facility specific management				

registration system in place that is linked to the to the national vital registration system at all times.		policy on routine review monitoring of care quality<br><br>2. Facility specific management policy on strict maintenance of data collection system and use<br><br>3. What does the facility do with data collected on patient care?				
3.The health facility has a system for classifying diseases and birth outcomes, including death, which is aligned with the ICD.		1. Facility specific management policy on routine review monitoring of care quality<br><br>2. Facility specific				

		management policy on strict maintenance of data collection system and use<br><br>3. What does the facility do with data collected on patient care?				
Output/process measures	F1.PSFR	F2.MI	Fom3	F4.OPCI	F5.CMRR	F6.WEICPC
1.The proportion of all newborns currently in the health facility who have a patient identifier and individual clinical medical record.	Is there data on numbers of sick newborns in this facility in a year?	1.Facility specific management policy on strict maintenance of data collection system and use<br><br>2.Facility specific management policy on routine review	Is baby weight taken/provided in this facility as a newborn signal function and was it done in the past 6 months?	1.What was the weight of the baby?<br><br>2. Record time of delivery?<br><br>3. Birth Attendant notes/shout time of delivery?	1.Is/are birth weights recorded for all babies?<br>2.If yes birth weight baby1/baby2/ba by3<br>3.Was the fetal heart rate recorded?	

		monitoring of care quality				
2.The proportion of all newborns discharged from the health facility within the past 24 h who had an accurately completed record of processes of care, treatments, outcomes and diagnoses (with ICD code)	1.Level of the facility? (hospital/health centre/tertiary facility)<br><br>2. Does this facility admit patients over night?<br><br>3. Does the facility have a separate maternity ward?<br><br>4. Does the facility have a separate paediatric ward?	1.Does the facility specific management policies include pre-discharge evaluation of mother and baby?<br><br>2. Pre-discharge counselling for mother and baby?	1.Does this facility offer 24hr services?<br><br>2. Does this facility operate a static child welfare clinic?<br><br>3. Is comprehensive Newborn Intensive Care Unit provided in this facility and was it provided in the past 6 months?<br><br>4. Do you routinely follow up mothers and newborns after discharge from the facility?		1.Date of delivery?<br><br>2.Time of delivery?<br><br>3.Date of discharge from the health facility?<br><br>4.Time of discharge from the health facility?<br><br>5. Was the woman thoroughly examined any time after delivery before discharge	If you had your way, how would you have preferred your length of stay after delivery?

3.The proportion of all women discharged postpartum within the past 24 h who had an accurately completed record of processes of care, treatments, outcomes and diagnoses (with ICD code)	1.Level of the facility? (hospital/health centre/tertiary facility)<br><br>2. Does this facility admit patients over night?<br><br>3. Does the facility have a separate maternity ward?	1.Does the facility specific management policies include pre-discharge evaluation of mother and baby?<br><br>2. Pre-discharge counselling for mother and baby?	1.Does this facility offer 24hr services?<br><br>2. Are delivery services available for 24hrs in a day?<br><br>3. Does this facility also admit pregnant women who come here and are considered not fit to go home?		1.Date and time of delivery?<br><br>2. Date and time of discharge from the health facility?<br><br>3. How long was the woman hospitalized before discharge?<br><br>4. Was the woman thoroughly examined any time after delivery before discharge	If you had your way, how would you have preferred your length of stay after delivery?
Quality statement 2.2						
Every health facility has a mechanism for data collection, analysis and feedback as part of its activities for monitoring and improving performance around the time of childbirth.						
Input measures	F1.PSFR	F2.MI	F3.SIV	Fom4	F5.CMRR	F6.WEICPC

1.The health facility has conducted reviews of maternal and perinatal deaths and near-misses at least once a month within the past six months and has a mechanism for implementing the recommendations of reviews	1.Does this facility routinely conduct audit for maternal deaths?<br><br>2. Does this facility routinely conduct audit for early neonatal deaths?<br><br>3. Does this facility routinely conduct audit for stillbirths?	1.The facility has specific management policies on systematic mortality audits?<br><br>2. The facility has specific management policies on systematic "near miss" audits.<br><br>3. facility has specific management policies on routine review monitoring of care quality.		1.Spontaneous breathing assessed at birth?<br><br>2. Newborn received additional stimulation?<br><br>3. Newborn begins to breath after additional stimulation.		
2.The health facility has standard operating procedures and	1.Does this facility routinely conduct audit	1.What are the main policies of this facility regarding maternity and	1.Have you received refresher training in the past 12 months		1.How many times was the woman's temperature checked and	1.Were you told your BP was high?

protocols in place at all times for checking, validating and reporting data.	for maternal deaths?<br>2. Does this facility routinely conduct audit for early neonatal deaths?<br>3. Does this facility routinely conduct audit for stillbirths?<br><br>4. Does this facility have a functional emergency patient register?<br><br>5. Does this facility admit patients over night?	newborn care? collect any points.<br>2. Does the facility have specific management policy(ies) on systematic mortality audits, systematic ”near-miss” audits, routine review monitoring of care quality, strict maintenance of data collection system and use?	on labour monitoring, partograph use, and care for the sick newborn?<br><br>2. Does this facility operate a static child welfare clinic (CWC)?<br><br>3. Is comprehensive Newborn Intensive Care Unit provided in this facility and was it done/provided in the past 6 months?<br><br>4. Kangaaro mother Care: Recorded evidence of KMC (prolonged		recorded between first exam and delivery?<br><br>2. How many times was blood pressure checked and recorded between first exam and delivery?<br><br>3. How many times was the mother’s pulse checked and recorded between first exam and delivery?<br><br>4. was the fetal heart rate (FHR) recorded	2. did the health worker before sending you labour ward checked your eyelids, tongue, or nails?<br><br>3. checked your BP<br><br>4. Take your urine and checked<br><br>5. checked whether your were bleeding?<br><br>6. listened to your baby’s heart beats?

			STSC, EBF/feeding support, growth monitoring (weight).<br><br>5. Do you routinely follow up mothers and newborns after discharge from the facility?		5. was the state of the membranes recorded?<br><br>6. was the state of the liquor recorded	
3.The health facility has a data system for collecting and analysing relevant indicators and can produce visual outputs and timely reporting on paper or digitally at all times.	1.Does this facility routinely conduct audit for maternal deaths?<br><br>2. Does this facility routinely conduct audit for early neonatal deaths?	1.Is there data on numbers of deliveries in this facility in a year?<br><br>2. Are there charts to show coverage and quality?<br><br>3. Is there data on numbers of sick newborn in this facility in a year?	1.Do you offer Antenatal Care to pregnant women in this facility?<br><br>2. Are delivery services available 24hrs in a day?<br><br>3. Does the facility offer immunization		1.How many times was the woman's temperature checked and recorded between first exam and delivery?<br><br>2. How many times was blood pressure checked and recorded between first	1.Were you told your BP was high?<br><br>2. Checked your BP<br><br>4. Take your urine and checked<br><br>5. checked whether your were bleeding?

	3. Does this facility routinely conduct audit for stillbirths?	4. Have you used the data to make programme and management decisions?<br><br>5. If yes, please describe the most recent example for maternal and newborn care?	(EPI vaccines) to children?<br><br>4. Does the facility operate a static child welfare clinic?<br><br>5. Do you routinely follow up mothers and newborns after discharge from the facility?		exam and delivery?<br><br>3. How many times was the mother's pulse checked and recorded between first exam and delivery?<br><br>4. was the fetal heart rate (FHR) recorded<br><br>5. was the state of the membranes recorded?<br><br>6. was the state of the liquor recorded	6. listened to your baby's heart beats?
4.Managers and health care workers in the health facility	1. Does this facility routinely conduct audit	1.Facility specific management policy on	Do you routinely follow up mothers and newborns after		1. Delivery outcome for woman	

met at least once a month within the past six months to review process and outcome data.	for maternal deaths?<br><br>2. Does this facility routinely conduct audits for early neonatal deaths?<br><br>3. Does this facility routinely conduct audits for stillbirths?	systematic mortality audits<br><br>2. Facility specific management policy on systematic "near miss" audits<br><br>3. Facility specific management policy on routine monitoring of care quality<br><br>4. Facility specific management policy on strict maintenance of data collection system and use	discharge from the facility?		2. Delivery outcome for newborn<br><br>3. If maternal death, what was the main underlying cause of death?<br><br>4. If stillbirth/early neonatal deaths, what was the primary underlying cause of death?	
5.Managers and health care	1.Does this facility	1.Facility specific	Do you routinely follow			

workers in the health facility used the recommendations in reviews of data for decision-making and for mentoring improved performance within the past six months.	routinely conduct audit for maternal deaths?<br><br>2. Does this facility routinely conduct audit for early neonatal deaths?<br><br>3. Does this facility routinely conduct audit for stillbirths?	management policy on systematic mortality audits<br><br>2. Facility specific management policy on systematic "near miss" audits<br><br>3. Facility specific management policy on routine monitoring of care quality<br><br>4. Facility specific management policy on strict maintenance of data collection system and use	up mothers and newborns after discharge from the facility?			

Output/process measures	F1.PSFR	F2.MI	F3.SIV	F4.OPCI	F5.CMRR	F6.WEICPC
1.The proportion of all perinatal deaths occurring in the health facility that were reviewed with standard audit tools.	1.Does this facility routinely conduct audit for early neonatal deaths?<br><br>2.Does this facility routinely conduct audit for stillbirths?	Does the facility have specific management policy on systematic mortality audits?			If stillbirth/early neonatal death, what was the primary underlying cause of death?	
2.The proportion of all maternal deaths and near-misses occurring in the health facility that were reviewed with standard audit tools.	1. Does this facility routinely conduct audit for maternal deaths?<br><br>2. Does this facility routinely conduct audit for early	1. Does the facility have specific management policy on systematic mortality audits?<br><br>2. Does the facility have specific			If maternal death, what was the main underlying cause of death?	

	neonatal deaths?<br><br>3. Does this facility routinely conduct audit for stillbirths?	management policy on systematic ”near-miss” audits?				
3.The proportion of all maternal deaths and near-misses occurring in the health facility that were notified.	1.Does this facility routinely conduct audit for maternal deaths?<br><br>2. Does this facility routinely conduct audit for early neonatal deaths?<br><br>3. Does this facility routinely conduct audit for stillbirths?	1.Does the facility have specific management policy on systematic mortality audits?<br><br>2.Does the facility have specific management policies on systematic ”near-miss” audits?			1.If maternal death, what was the main underlying cause of death?<br><br>2. which obstetric complications occurred during labour and/ or delivery and/ or immediate postpartum?	

Outcome measures	F1.PSFR	F2.MI	F3.SIV	F4.OPCI	F5.CMRR	F6.WEICPC
1.Data are collected routinely in the health facility during labour, childbirth and the postnatal period and used regularly to make decisions on quality improvement.	1.Does this facility routinely conduct audit for maternal deaths?<br><br>2. Does this facility routinely conduct audit for early neonatal deaths?<br><br>3. Does this facility routinely conduct audit for stillbirths?<br><br>4. Does the facility have emergency functioning register?	1.Does the facility have a quality improvement programme which include, i. quality improvement teams, ii. quality improvement action plans, iii. quality improvement mentoring/coaching, iv. capturing and use of data, v. monitoring and evaluation?<br><br>2. Do you have a system to make postnatal home visits?		1.History taken?<br><br>2. General examination done including the nails/eyelids/tongue for anaemia<br><br>3. Abdominal palpation for uterine contractions<br><br>4. Listened to the fetal heart beats<br><br>5. Blood pressure taken<br><br>6. Urine test done	1.How many times was the woman's temperature checked and recorded between first exam and delivery?<br><br>2. How many times was blood pressure checked and recorded between first exam and delivery?<br><br>3. How many times was the mother's pulse checked and recorded between first	

		3. How are data from the postnatal home visits and facility care linked?		Vaginal examination done	exam and delivery?<br><br>4. was the fetal heart rate (FHR) recorded<br><br>5. Was the state of the membranes and liquor recorded?	
2.The proportion of all recommendations in perinatal death reviews at the health facility that have been fully implemented.	1.Does this facility routinely conduct audit for maternal deaths?<br><br>2. Does this facility routinely conduct audit for early neonatal deaths?<br><br>3. Does this facility	1.Does the facility have specific management policy on systematic mortality audits?<br><br>2.Does the facility have specific management policies on systematic ”near-miss” audits?			If stillbirth/early neonatal death, what was the primary underlying cause of death?	

	routinely conduct audit for stillbirths?	3. Facility specific management policy on routine monitoring of care quality<br><br>4. Facility specific management policy on strict maintenance of data collection system and use				
3.The proportion of all recommendations in maternal death reviews at the health facility that have been fully implemented.	1.Does this facility routinely conduct audit for maternal deaths?<br>2. Does this facility routinely conduct audit for early	1.Does the facility have a specific policy on Systematic mortality audits?<br>2.Does the facility have a specific policy on systematic" near miss" audits.			1.If maternal death, what was the main underlying cause of death?	

	neonatal deaths?<br>3. Does this facility routinely conduct audit for stillbirths?	3.Specific policy on routine review monitoring of care quality care?				
Standard 3: Every woman and newborn with condition(s) that cannot be dealt with effectively with the available resources is appropriately referred.						
Quality statement 3.1						
Every woman and newborn is appropriately assessed on admission, during labour and in the early postnatal period to determine whether referral is required, and the decision to refer is made without delay.						
Input measures	F1.PSFR	F2.MI	F3.SIV	F4.OPCI	F5.CMRR	F6.WEICPC
1.The health facility has written, up-to-date clinical protocols and guidelines for the identification, management (including pre-referral care) and referral of women with complications		1.What are the main policies of this facility regarding maternity and newborn care? collect any points.<br>2.Do you have a system to refer and transfer a woman to a higher level of care?	Have you received refresher/in-service training in the past 12 months on i. Obstetric triage/initial assessment; ii. Management of Pre-eclampsia and/ or eclampsia?		1.Did the woman suffer from Eclampsia complication during pregnancy?<br><br>2.Did Pre-eclampsia/eclampsia occur as an obstetric complication during labour, and/ or delivery	

related to pregnancy and childbirth and in newborns.		3. If yes, do you have guidelines for such referral? Take a copy.			and/ or immediately postpartum?	
2.The health facility is equipped with appropriate medicines and medical supplies for stabilization and pre-referral treatment for referred women and newborns	Health facility availability of essential drugs (IV antibiotics, Magnesium Sulphate (MgS04), Diazepam, Oxytocic (syntometrine/Ergot)), Dexamethasone /Betamethasone (parenteral), Hydralazine/SL Nifedipine, Ampicillin/Penicillin for IV, Gentamicin for IV, Cephalosporins)	1.Do you have a system to refer and transfer a woman to a higher level?<br><br>2. If yes, do you have guidelines for such referral?	In your practice, what do you do to ensure safe and timely referrals?<br><br>How can you ensure that a newborn is warm during referral?	1.Was woman referred from another facility to this one?<br><br>2.Oxygen cylinder with oxygen<br>Ambu bag<br><br>3.Neonatal mask<br><br>4.Oxytocin<br><br>5.Clean gauze pack	1.Did the newborn receive any antibiotic?<br><br>2. Was the newborn given oxygen?<br><br>3. What was done with the woman with a pregnancy complication?<br><br>4.What was done with a woman with an obstetric complication?	Were you given any of the IV fluid/medicine to take/injection(IM), pain relief?
3.Health care staff in the	Does this facility refer	1.What additional	1.Have you ever have to refer a		What as done with a woman	

maternity unit receive in-service training and regular refresher sessions in referral protocols and guidelines at least once every 12 months	patients to other facilities?	training is provided to workers posted in maternity and children's wards?<br><br>2. Are there additional refresher training for the staff?<br><br>3. Do you have a system to refer and transfer women to a higher level of care?<br><br>4. If yes, do you have guidelines for such referral?	mother to another facility?<br><br>2.In your practice, what do you do to ensure safe and timely referrals?<br><br>3.Have you ever have to refer a newborn to another facility?<br><br>4.How can you ensure that a newborn is warm during referral?		with obstetric complications during labour, and/ or delivery, and/ or immediate postpartum? Referred to another facility?/admitte d and managed?/ not recorded?/ not applicable	
Output/process measures	F1.PSFR	F2.MI	F3.SIV	F4.OPCI	F5.CMRR	F6.WEICPC

1.The proportion of women and newborns seen in the health facility in the past three months who fulfilled the facility's criteria for referral who were actually referred.	1. Do you have a system to refer and transfer women to a higher level of care?<br><br>2. If yes, do you have guidelines for such referral?	1. Do you have a system to refer and transfer women to a higher level of care?<br><br>2. If yes, do you have guidelines for such referral?<br><br>3. where do you refer most newborns to?	Have you ever have to refer a newborn to another facility?		1. What was done for the baby (ies) with a complication (whether alive or dead)? Was the baby i) Referred to another facility; ii) admitted and managed; 8) not recorded; 9) not applicable, no complications.<br><br>2. What was done with a woman with an obstetric complications/problem that occurred during labour, and/or delivery, and/ or immediately postpartum? Was the women i) Referred to	

					another facility; ii) admitted and managed; 8) not recorded; 9) not applicable, no complications.	
2.The proportion of all pregnant or postpartum women who could not be managed at the health facility who were transferred to a higher-level facility for childbirth or further management without delay, accompanied by a health care professional and a completed standardized referral note.				Was woman referred from another facility to this one?	1.What was done with a woman with an obstetric complications/problem that occurred during labour, and/or delivery, and/ or immediately postpartum? Was the women 1) Referred to another facility; 2) admitted and managed; 8) not recorded; 9) not applicable, no complications?	Did you plan during pregnancy to deliver in this facility? iii. No, I was referred from another facility?

3.The proportion of all sick, preterm or small newborns who could not be managed at the health facility who were transferred to an appropriate level of care within 1 hour of a decision, accompanied by a health care professional and a complete standard referral note.	Do you have guidelines for referral to a higher level of care?			Was woman referred from another facility to this one?	1.What was done with a woman with an obstetric complications/problem that occurred during labour, and/or delivery, and/ or immediately postpartum? Was the women 1) Referred to another facility; 2) admitted and managed; 8) not recorded; 9) not applicable, no complications?	Did you plan during pregnancy to deliver in this facility? iii. No, I was referred from another facility?
Outcome measures	F1.PSFR	F2.MI	F3.SIV	F4.OPCI	F5.CMRR	F6.WEICPC
1.The proportion of all women admitted to the labour ward who				1.Time of arrival of a woman?	1. Date of presentation at the health facility	How long did you have to wait at the facility before you were first seen by a

reported receiving immediate attention on arrival at the health facility.				2.Time of first professional HW contact?	2. Time of presentation at the health facility<br><br>3. Date and time of delivery	health care professional when you first came in for this delivery?
Quality statement 3.2						
For every woman and newborn who requires referral, the referral follows a pre-established plan that can be implemented without delay at any time.						
Input measures	F1.PSFR	F2.MI	F3.SIV	F4.OPCI	F5.CMRR	F6.WEICPC
1.The health facility has ready access to a functioning ambulance or other vehicle for emergency transport of women and newborns to referral facilities.	1.Does this facility have an ambulance?	1.Do you have a system to refer and transfer a woman to a higher level of care?<br>2. if yes, what type of transportation do you provide for such referrals?<br>3. if yes, is transportation available 24/7	In your practice, what do you do to ensure safe and timely referrals?	1.Does the facility have ambulance access to reception?	What was done for the baby(ies) with the above complication?<br>i. Referred to another facility?<br>ii. admitted and managed, iii. Not recorded,<br>iv. 9=Not applicable, no complication	Did you plan during pregnancy to deliver in this facility?<br>iii. No, I was referred from another facility?

2.There is an up-to-date list of network facilities in the same geographical area that provide referral care for women and children.	Does this facility refer patients to other facilities?	1.Do you have a system to refer and transfer women to a higher level of care?<br>2. If yes, do you have guidelines for such referral?<br>3. Take a copy Where do you refer to most of the babies?<br>4. What means of functional communication s are available in the facility for staff to contact higher level referral facilities during referral for support?	1.Have you ever have to refer a mother to another facility?<br><br>2. Have you ever have to refer a newborn to another facility?	Was woman referred from another facility to this one?	1. What was done for the baby(ies) with the above complication?<br>i. Referred to another facility?<br>ii. admitted and managed, iii. Not recorded,<br>iv. 9=Not applicable, no complication<br><br>2. what was done for the woman with an obstetric complication?<br>i. Referred to another facility?<br>ii. admitted and managed, iii. Not recorded,<br>iv. 9=Not applicable, no complication	Did you plan during pregnancy to deliver in this facility?<br>iii. No, I was referred from another facility?

3.The health facility has local arrangements to ensure that women and newborns who cannot be managed at the health facility are referred to an appropriate level of care without delay, 24 h a day, 7 days a week.	1. Do you have a system to refer and transfer women to a higher level of care?<br>2. If yes, do you have guidelines for such referral?	1. Do you have a system to refer and transfer a woman to a higher level of care?<br>2. if yes, what type of transportation do you provide for such referrals?<br>3. if yes, is transportation available 24/7?	1.Have you ever have to refer a mother to another facility?<br>2. In your practice, what do you do to ensure safe and timely referrals?<br>3. Have you ever have to refer a newborn to another facility?<br>4. How can you ensure that a newborn is warm during referral?	Was woman referred from another facility to this one?	1. What was done for the baby(ies) with the above complication?<br>i. Referred to another facility?<br>ii. admitted and managed, iii. Not recorded,<br>iv. 9=Not applicable, no complication<br><br>2. what was done for the woman with an obstetric complication?<br>i. Referred to another facility?<br>ii. admitted and managed, iii. Not recorded,<br>iv. 9=Not applicable, no complication	Did you plan during pregnancy to deliver in this facility?<br>iii. No, I was referred from another facility?

Output/process measures	F1.PSFR	F2.MI	F3.SIV	F4.OPCI	F5.CMRR	F6.WEICPC
1. The proportion of all newborns who died before or during transfer to a higher-level facility for further management. F1.PSFR- audit	1. Does this facility routinely conduct audit for early neonatal deaths?<br><br>2. Does this facility routinely conduct audit for stillbirths?<br><br>3. Does this facility have a functional emergency patient register?	1. Facility specific management policy on systematic mortality audits.<br><br>2. Facility specific management policy on routine review monitoring of care quality	Have you ever had to refer a newborn to another facility?		If stillbirth/early neonatal death, what was the primary underlying cause of death?	
2.The proportion of all pregnant or postpartum women who died before or during transfer	1. Does this facility routinely conduct maternal deaths?	1. Facility specific management policy on systematic mortality audits.	Have you ever have to refer a mother to another facility?		If maternal death, what was the main underlying cause of death?	

to a higher-level facility for childbirth for further management. F1.PSFR- audit	2. Does this facility have a functional emergency patient register?	2. Facility specific management policy on routine review monitoring of care quality				
3.The proportion of pregnant and postpartum women and newborns who were referred without appropriate emergency transport.	1.Does this facility have an ambulance?<br><br>2. Does the facility have ambulance access to reception?	1.Do you have a system to refer and transfer women to a higher level of care?<br>2. If yes, do you have guidelines for such referral?<br>3. What type of transport do you provide for referral to a higher level of care? i) patient's arranged transport; ii) facility ambulance; iii)	1.Have you ever have to refer a mother to another facility?<br>2. In your practice, what do you do to ensure safe and timely referrals?<br>3. Have you ever have to refer a newborn to another facility?<br>4. How can you ensure that a newborn is warm during referral?	Was woman referred from another facility to this one?	1. What was done for the baby(ies) with the above complication?<br>i. Referred to another facility?<br>ii. admitted and managed, iii. Not recorded,<br>iv. 9=Not applicable, no complication<br><br>2. what was done for the woman with an obstetric complication?	Did you plan during pregnancy to deliver in this facility?<br>iii. No, I was referred from another facility?

		facility tricycle (motor-king); iv) other; 9) NA, no referrals.<br>4. Is facility transportation available 24/7?			i. Referred to another facility?<br>ii. admitted and managed, iii. Not recorded,<br>iv. 9=Not applicable, no complication	
4.The proportion of all women referred from the health facility who contributed financially to communication or to transport.		1.Facility has specific management policies on free emergency care.<br><br>2. Facility has specific management policies on free delivery care<br><br>3.Do women have to pay for the use of ambulance?	1. in case of an obstetric emergency, is payment required before a woman can receive treatment?<br><br>2. Is the woman expected to pay a fee if she brings her newborn to the facility for care?	Are women supposed to pay for maternity services?<br><br>Did the woman or her family pay any monies for the services provided in the facility?		Did you have to pay anything (cash or kind) for the service provided during labour or delivery?
Outcome measures	F1.PSFR	F2.MI	F3.SIV	F4.OPCI	F5.CMRR	F6.WEICPC

1.The proportion of all women referred from the health facility who completed their referral.	Does this facility have a functional emergency register?	1. Do you have a system to refer and transfer women to a higher level of care?<br><br>2. If yes, do you have guidelines for such referral?	1. Have you ever have to refer a mother to another facility?<br><br>Vignette:<br>2. Refer to other health facility immediately<br><br>3. Refer to hospital where Caesarean section can be done			
2.The proportion of all newborns referred from the health facility who completed their referral.	Does this facility have a functional emergency register?	1. Where do you refer newborns to?<br><br>2. If yes, do you have guidelines for such referral?	1. Have you ever had to refer a newborn to another facility?<br><br>Vignette:<br>2. Refer to a hospital/anothe r facility			
3.The proportion of all newborns referred from	Does this facility have a functional	1. Have you ever had to refer a newborn to another facility?	1. Have you ever had to refer a newborn to another facility?			

the facility who reached the referral facility without hypothermia.	emergency register?	Vignette: 2. Refer to a hospital/another facility	Vignette: 2. Refer to a hospital/another facility			
Quality statement 3.3						
For every woman and newborn referred within or between health facilities, there is appropriate information exchange and feedback to relevant health care staff.						
Input measures	F1.PSFR	F2.MI	F3.SIV	F4.OPCI	F5.CMRR	F6.WEICPC
1.The health facility has a standardized referral form to document relevant demographic and clinical information, which includes clinical findings, diagnosis, pre-referral interventions or treatment given and reason for referral.	Does this facility refer patients to other facilities?	1.Do you have a system to refer and transfer women to a higher level of care? 2.If yes, do you have guidelines for such referral? 3.What means of functional communications are available in the facility for staff to contact higher level referral			1. What was done for the baby(ies) with the above complication? i. Referred to another facility? ii. admitted and managed, iii. Not recorded<br><br>2. what was done for the woman with an obstetric complication? i. Referred to another facility?	

		facilities during referral for support?			ii. admitted and managed, iii. Not recorded.	
2.The health facility has reliable communication methods, including a mobile phone, land line or radio, which is functioning at all times, for referrals and consultation on complicated cases.	Does the facility have landline/mobile phone/radio phones?	What means of functional communications are available in the facility for staff to contact higher level referral facilities during referral for support?				
3.Evidence that the health facility has formal agreements, communication arrangements and a feedback system with	1. Does this facility refer patients to other facilities?<br><br>2. Does this facility have an ambulance?	1. Do you have a system to refer and transfer a woman to a higher level of care?<br><br>2. what means of functional communication	In your practice, how do you ensure safe and timely referrals?		1. What was done for the baby(ies) with the above complication?<br>i. Referred to another facility?<br>ii. admitted and managed, iii. Not recorded	

referral centre(s).		s are available in the facility for staff to contact higher level referral facilities during referral for support?			2. what was done for the woman with an obstetric complication?<br>i. Referred to another facility?<br>ii. admitted and managed, iii. Not recorded.	
Output/process measures	F1.PSFR	F2.MI	F3.SIV	F4.OPCI	F5.CMRR	F6.WEICPC
1.The proportion of all referred women seen at the referring facility for whom there was complete counter-referral feedback information.	Emergency patient register	1. Do you have a system to refer and transfer women to a higher level of care?<br><br>2. Do you have guidelines for such referral?	Have you ever have to refer a mother to another facility?	Was woman referred from another facility to this one?	1.Was the woman referred from another facility?<br><br>2.Was the woman with a pregnancy complication referred to another facility?<br><br>3.Was the woman with an	Did you plan during pregnancy to deliver in this facility?<br>iii. No, I was referred from another facility?

					obstetric complication during labour, delivery or immediate postnatal referred to another facility?	
2.The proportion of all referred newborns seen at the referring facility for whom there was complete counter-referral feedback information.			Have you ever had to refer a newborn to another facility?	Was woman referred from another facility to this one?	1Was done/were done for the baby(ies) with the above complications?	Did you plan during pregnancy to deliver in this facility?<br>iii. No, I was referred from another facility?
3.The proportion of all referred women and newborns seen at the referring facility who received timely care at			In your practice, how do you ensure safe and timely referral?	Time of arrival of a woman?<br><br>Time of first professional HW contact?	What was done for the woman with an obstetric complication?<br>i. Referred to another facility?	How long did you have to wait at the facility before you were first seen by a health care professional when you first

the referral facility.					ii. admitted and managed, iii. Not recorded.	came in for this delivery?
Standard 4: Communication with women and their families is effective and responds to their needs and preferences.						
Quality statement 4.1						
All women and their families receive information about the care and have effective interactions with staff.						
Input measures	F1.PSFR	F2.MI	F3.SIV	F4.OPCI	F5.CMRR	F6.WEICPC
1. Easily understood health education materials, in an accessible written or pictorial format, are available in the languages of the communities served by the health facility.		1. Are community stakeholders involved in quality improvement efforts in your facility?<br><br>2. If yes, can you briefly describe how they are involved?<br><br>3. Are there women's support group in the community	Does the facility have clear policies on rights of the patient?<br><br>Are women's support groups available in the community?<br><br>Vignette:<br>Have somebody stay with her all the time in case she starts having seizures	Communication between health worker and client<br><br>Was any companion allowed to be present during delivery?	Was the woman counselled on maternal danger signs/newborn danger signs/breastfeeding/care seeking for illness/postpartum care/immunization?	1. Are you satisfied with the information you received about breastfeeding/postpartum care and hygiene/Family Planning?

		served by this facility?<br><br>If yes, do you have any formal link/communication with these women's support group? If yes, please describe?				
2.Health care staff in the maternity unit are oriented and receive in-service training at least once every 12 months to improve their interpersonal communication and counselling skills and cultural competence.		1.What additional training is provided to workers posted there?<br><br>2. Are there additional refresher training for the staff?<br><br>3. How often have they been trained over the past year?	1.Have you received refresher/in-service training in the past 12 months?<br><br>2. If yes, was it on breastfeeding counselling and lactation management?	1. Was the woman counselled on maternal danger signs?<br><br>2. Was the woman counselled on newborn danger signs?	Was the woman counselled on any of maternal danger signs/newborn danger signs/breastfeeding, care seeking for illness/postpartum care/immunization	1.Were you told about danger signs after childbirth for the mother- when you have to see care again at the health facility?<br><br>2.Were you told about danger signs after childbirth for the newborn – when you have to seek care at a

						nearest health facility?
3.The health facility has a written, up-to-date policy that outlines clear goals, operational plans and monitoring mechanisms to promote the interpersonal communication and counselling skills of health care staff.		1.What are the main policies of this facility regarding maternity and newborn care? collect any points.<br><br>2. Routine review monitoring of care quality?	1.Have you received refresher/in-service training in the past 12 months?<br><br>2. If yes, was it on breastfeeding counselling and lactation management?	1. Was the woman counselled on maternal danger signs?<br><br>2. Was the woman counselled on newborn danger signs?	Was the woman counselled on any of maternal danger signs/newborn danger signs/breastfeeding, care seeking for illness/postpartum care/immunization	1.Were you told about danger signs after childbirth for the mother- when you have to see care again at the health facility?<br><br>2.Were you told about danger signs after childbirth for the newborn – when you have to seek care at a nearest health facility?
4.Health care staff in the maternity unit receive supportive supervision in interpersonal		1.Do you receive external supervisory visits to this facility?	1.Have you received refresher/in-service training in the past 12 months?	Was the woman counselled on maternal danger signs?<br><br>Was the woman counselled on	Was the woman counselled on any of maternal danger signs/newborn danger signs/breastfeed	1.Were you told about danger signs after childbirth for the mother- when you have to see care again

communication, counselling and cultural competence every three months.		2.How often do you receive these external visits?<br>3.When was the last external supervisory visit? Record the date.	2. If yes, was it on breastfeeding counselling and lactation management?	newborn danger signs?	ing, care seeking for illness/postpartum care/immunization	at the health facility?<br><br>2.Were you told about danger signs after childbirth for the newborn – when you have to seek care at a nearest health facility?
Output/process measures	F1.PSFR	F2.MI	F3.SIV	F4.OPCI	F5.CMRR	F6.WEICPC
1.The proportion of all women discharged from the labour and childbirth area of the facility who received written and verbal information and counselling on the following	Does the facility have functioning graduated cup to measure expressed breastmilk?<br><br>Does the facility have functioning small cup for feeding	1.Facility specific management policy on Baby friendly<br><br>2. Facility specific management policy on Early Initiation of breastfeeding	1.Does the facility offer contraceptive services?<br><br>2. Is postpartum family planning services available?<br><br>3. Is Early Initiation and exclusive	1.Counselled on danger signs for the newborn?<br><br>2.Counselled on danger signs for the mother?<br><br>3.Counselled on postnatal care attendance?	1.Was the woman counselled on any of Maternal danger signs?<br><br>2. Was the woman counselled on newborn danger signs?	1.Are you satisfied with the information you received on breastfeeding?<br><br>2.Do you feel competent enough to be able to breastfeed your baby?

elements before discharge: nutrition and hygiene, birth spacing and family planning, exclusive breastfeeding and maintaining lactation, keeping their baby warm and clean, communication and play with the baby, danger signs for the mother and newborn and where to go in case of complications.	expressed breast milk?	3. Facility specific management policy on Exclusive breastfeeding or breastmilk feeding	breastfeeding provided for in this facility as a newborn signal function and was it provided/done in the past 6 months?<br><br>4. Have you received refresher/in-service training in the past 12 months on breastfeeding counselling and lactation management?<br><br>Vignettes:<br>5. Watch her breastfeed her baby and teach her good positioning and attachment.	4.Counselled on family planning?	3. Was the woman counselled on breastfeeding?<br><br>4. Was the woman counselled on care seeking for illness?<br>5. Was the woman counselled on postpartum care and immunization?	3.Are you satisfied with the information you received on family planning?<br><br>4.Are you satisfied with the information you received on postpartum care and hygiene?<br><br>5.Were you told about danger signs after childbirth for the mother, and when you have to seek care again at the health facility?<br><br>6.Were you told about danger signs after childbirth for

			6. If baby not breastfeeding, teach her to express the milk and feed with a clean cup<br><br>7. Educate her and encourage her to practice exclusive breastfeeding for the 1st 6 months of the baby's life.			the newborn, and when you have to seek immediate care at the nearest health facility?
2.The proportion of all women who gave birth in the health facility who reported that they were given the opportunity to discuss their concerns and preferences.	Is there a complaint/suggestions box or other means of receiving anonymous feedback from clientele?	1.Does the training sessions cover respectful maternity care?<br><br>2. Does the training sessions cover patient's rights?	1. Is there a policy and procedure for addressing patient concerns in this facility?<br><br>2. Have you received training on how to treat	1.Did the woman request for anything and was not given?<br><br>2. Was woman told respectfully why the request was refused (if yes above)?		1.Did they allow you to ask questions when you wanted?<br><br>2.Do you think health workers were responsive when you asked for their support?

		3. Facility specific management policy on respectful and dignified care for all?	childbearing women with compassion and dignity (customer care)?<br><br>3. Are community stakeholders involved in addressing disrespect and abuse of women during childbirth?			
3. The proportion of health care staff in the health facility who demonstrated the following skills: active listening, asking questions, responding to questions,				1. Feedback to mother after examination?<br><br>2. Plan for delivery communicated to mother (SVD/Assisted vaginal delivery/C/S)		1. Did they tell you what they found before sending you to the labour ward?<br><br>2. How would you describe the way the health workers talked to you in the

verifying the understanding of women and their families and supporting women in problem-solving.				3. Attitude of the health workers when woman is in pain?<br><br>4. Did the woman request for anything and was not given?		facility when you first come in?<br><br>3. Did they allow you to ask questions when you wanted?<br><br>4. Do you think the health workers were responsive when you ask for their support?
Outcome measures	F1.PSFR	F2.MI	F3.SIV	F4.OPCI	F5.CMRR	F6.WEICPC
1.The proportion of all women who gave birth in the health facility who felt they were adequately informed by the care provider(s)		1.Does the training sessions cover respectful maternity care?<br><br>2. Does the training	Does your facility have clear policies on rights of the patient?<br><br>Have you received training on how	1.Feedback to mother after examination?<br><br>2.Plan for delivery communicated to mother(SVD/ass		Did they tell you what they found before sending you to the labour ward?

about the examinations, actions and decisions taken for their care.		sessions cover patient's rights?<br><br>3. Facility specific management policy on respectful and dignified care for all?	to treat childbearing women with compassion and dignity (customer care)?	isted vaginal delivery/C/S)?		
2.The proportion of all women who gave birth in the health facility who reported that their needs and preferences were taken into account during labour, childbirth and postnatal care.		1.Does the training sessions cover respectful maternity care?<br><br>2. Does the training sessions cover patient's rights?<br><br>3. Facility specific management policy on respectful and dignified care for all?	Does your facility have clear policies on rights of the patient?<br><br>Have you received training on how to treat childbearing women with compassion and dignity (customer care)?	1.Feedback to mother after examination?<br><br>2.Plan for delivery communicated to mother(SVD/assisted vaginal delivery/C/S)?		1.Do you think the health workers took your concerns into considerations in providing care during your stay there?<br><br>2.Was there any time that you were concerned that they were not doing enough for you and the baby?

						3.Do you think that health workers were responsive when you asked for their support?
3.The proportion of all women who gave birth in the health facility who expressed satisfaction with the health services.	Is there a complaint/suggestions box or other means of receiving anonymous feedback from clientele?	1.Does the training sessions cover respectful maternity care?<br><br>2. Does the training sessions cover patient's rights?<br><br>3. Facility specific management policy on respectful and dignified care for all?	1.Does your facility have clear policies on rights of the patient?<br><br>2. Have you received training on how to treat childbearing women with compassion and dignity (customer care)?	1.Did the woman request for anything and was not given?<br><br>2. Was woman told respectfully why the request was refused (if yes above)?<br><br>3. Feedback to mother after examination?<br><br>4. Plan for delivery communicated to mother(SVD/ass		1. Are you satisfied with the level of attention and care given to your baby after delivery?<br><br>2.In general, how satisfied are you with the care you received during your delivery at this facility?

				isted vaginal delivery/C/S)?		
4.The proportion of all women who gave birth in the health facility who reported that they were satisfied with the health education and information they received from care providers.	Is there a complaint/suggestions box or other means of receiving anonymous feedback from clientele?	1.Do the training sessions cover professional ethics?<br><br>2. Do the training sessions cover respectful maternity care?<br><br>3. Do the training sessions cover patient's rights?<br><br>4. Facility specific policies on Baby friendly/early initiation of breastfeeding/exclusive breastfeeding or breastmilk	1.Is there a policy and procedure for addressing patient concerns in this facility?<br><br>2. Does your facility have clear policies on rights of the patient?<br><br>3. Have you received training on how to treat childbearing women with compassion and dignity (customer care)?	Was the woman counselled on maternal danger signs?<br><br>Was the woman counselled on newborn danger signs?	Was the woman counselled on any of maternal danger signs/newborn danger signs/breastfeeding, care seeking for illness/postpartum care/immunization	1.Are you satisfied with the information you received about breastfeeding?<br>2.Do you feel you are competent enough to be able to breastfeed your baby on your own?<br><br>3.Are you satisfied with the information you received about postpartum care and hygiene?<br><br>4.Are you satisfied with the information

		feeding/respectful and dignified care for all				you received family planning?
Quality statement 4.2						
All women and their families experience coordinated care, with clear, accurate information exchange between relevant health and social care professionals.						
Input measures	F1.PSFR	F2.MI	F3.SIV	F4.OPCI	F5.CMRR	F6.WEICPC
1.The health facility has a standard form for clinical progress notes and monitoring events during labour (partograph), birth and after birth to facilitate written hand-over.	Does the facility admit patients over night?	How does the facility monitor its performance?	Does this facility offer 24 hours services?<br><br>2. Is this facility open to offer services all weekends?<br><br>3. Are delivery services available 24hrs in a day?	1.Was she started on partograph?<br>2. started a partograph in the labour ward?<br>3. How often were partographs filled after examination?	1. Was the surgical findings recorded?<br>2. Time partograph was started?<br>3. What type of partograph is used for this client at the facility?<br>4. what type of partograph is used for this client at the facility?	
2.The health facility has written, up-to-date protocols	Does this facility refer patients to other facilities?	1.What are the main policies of this facility regarding	1.Have you ever have to refer a mother to another facility?	Was woman referred from another facility to this one?		Did you plan during pregnancy to deliver in this

for verbal and written hand-over of women and newborns at shift changes, during intra-facility transfer, on referral to other facilities and at discharge.		maternal and newborn care? collect any points.<br><br>2.Do you have a system to refer and transfer a woman to a higher level of care?<br><br>3. If yes, do you have guidelines for such referral?	2. In your practice, what do you do to ensure safe and timely referrals?<br><br>3. Have you ever had to refer a newborn to another facility?<br><br>4. How can you ensure that a newborn is warm during referral?			facility? (No, was referred from another facility; No, was brought in an emergency, yes, always attended ANC here)
3.Health-care staff in the maternity unit are oriented and receive in-service training and regular refresher sessions at least once every 12 months in the	Does this facility refer patients to other facilities?	1.What additional training is provided to workers posted in maternity and children's wards?<br>2. Are there additional refresher	1.Have you received refresher /in-service training in the past 12 month on i) essential care during labour and childbirth? ii) labour monitoring?	Was the woman counselled on maternal danger signs?<br><br>Was the woman counselled on newborn danger signs?	Was the woman counselled on any of maternal danger signs/newborn danger signs/breastfeed ing, care seeking for illness/postpart um	1.Are you satisfied with the information you received about breastfeeding?<br><br>2.Are you satisfied with the information you received

clinical hand-over policy and communication of important information for hand-over, referral or discharge.		training for the staff?<br>3. Have there been training over the past year?<br>4. How often have they been trained over the past year?	iii) partograph use?<br>iv) management of prolonged and/ or obstructed labour?<br><br>2. Have you received training on how to treat childbearing women with compassion and dignity (customer care)?		care/immunizati on	about postpartum care and hygiene?<br><br>3. Are you satisfied with the information you received family planning?
4.The health facility has a functioning, reliable communication system for information exchange among relevant	Does the facility have functioning landline/mobile phone/radio phones?	1.What means of functional communication s are available in the facility for staff to contact higher level referral for support?	1.Have you ever have to refer a mother to another facility?<br><br>2. In your practice, what do you do to			

services providers.		2. Facility specific management policy on routine review monitoring of care quality<br><br>3. How does the facility monitor its performance?	ensure safe and timely referrals?<br><br>3. Have you ever had to refer a newborn to another facility?<br><br>Vignette:<br>4. Refer to other health facility immediately<br><br>5. Refer to hospital where Caesarean section can be done			
Output/process measures	F1.PSFR	F2.MI	F3.SIV	F4.OPCI	F5.CMRR	F6.WEICPC
1.The proportion of women attended during labour and childbirth for whom a	1. Does this facility have a separate maternity ward?	1. Facility specific management policy on routine review monitoring of care quality.	Have you received refresher/in-service training in the past 12 months on partograph use?	Was she started on the partograph, after initial examination on admission?	1.Was a partograph started in the labour ward?<br><br>2.What was the woman's	

partograph has been completed.		2. Facility specific management policy on strict maintenance of data collection system and use			dilatation when the partograph was started?<br><br>3.What was the woman's dilatation on the alert line of the partograph when it was started?<br><br>4.How many times was a vaginal examination carried out and recorded between first exam and delivery (including first exam and delivery).	
Outcome measures	F1.PSFR	F2.MI	F3.SIV	F4.OPCI	F5.CMRR	F6.WEICPC

1.The proportion of all women who gave birth in the health facility who expressed satisfaction with the health services.		1. Do facility training sessions cover patient's rights?<br><br>2. Facility specific management policy on respectful and dignified care for all	1. Is there a policy and procedure for addressing patient concerns in this facility?<br><br>2. Does your facility have clear policies on rights of the patient?<br><br>3. Have you received training on how to treat childbearing women with compassion and dignity (customer care)?	1. Did the woman request for anything and was not given?<br><br>2. Was woman told respectfully why the request was refused (if yes above)?		In general, how satisfied are you with the care you received during your delivery at this facility?
Standard 5: Women and newborns receive care with respect and can maintain their dignity.						
quality statement 5.1						
All women and newborns have privacy around the time of labour and childbirth, and their confidentiality is respected.						

Input measures	F1.PSFR	F2.MI	F3.SIV	F4.OPCI	F5.CMRR	F6.WEICPC
1.The physical environment of the health facility allows privacy and the provision of respectful, confidential care, including the availability of curtains, screens, partitions and sufficient bed capacity.	Does the facility has functioning screen/curtain for privacy?	Does additional refresher training for staff posted to maternity and children's ward cover professional ethics, respectful maternal care, abuse, patient's rights?	1. Does your facility have clear policies on rights of the patient?<br><br>2. Does your facility have clear policies on professional ethics?<br><br>3. Are delivery services available for 24hrs in a day?	1.During delivery, was privacy assured?<br><br>2. Was the woman's privacy maintained during the examination?		1.Were you worried that other people were seeing you when you were being examined by the staff/health care workers.<br><br>2.Were you worried that other people were hearing you when you were being examined by the staff/health care workers.<br><br>3.Do you think health workers treated you with respect?
2.The health facility has written, up-to-	Does the facility has functioning	1. Does the facility have specific	1. Does your facility have clear policies on	1. During delivery, was privacy assured?		1. Were you worried that other people

date protocols to ensure privacy and confidentiality for all women and newborns in all aspects of care.	screen/curtain for privacy?	management policy on Respectful and dignified care for all?<br><br>2. Does additional refresher training for staff posted to maternity and children's ward cover professional ethics, respectful maternal care, abuse, patient's rights?	rights of the patient?<br><br>2. Does your facility have clear policies on professional ethics?	2. Was the woman's privacy maintained during the examination?		were seeing you when you were being examined by the staff/health care workers.<br><br>2.Were you worried that other people were hearing you when you were being examined by the staff/health care workers.<br><br>3. Do you think health workers treated you with respect?
3.The health facility has accountability mechanisms for redress in the event of violations of	Is there a complaint/suggestions box or other means of receiving anonymous	1.What happens if a unit or person is found not to be performing well?	1.Is there a policy and procedure for addressing patient concerns in this facility?			1.Do you think health workers treated you with respect?<br><br>2. Were you worried that

privacy, confidentiality or consent.	feedback from clientele?	2.Are there reward sanctions?<br><br>3. Are women encouraged to report such abuses from health professionals to managers?<br><br>4. what measures did you take to prevent future occurrence of such abuse?	2. Does your facility have clear policies on rights of the patient?			other people were seeing you when you were being examined by staff/health care workers?
Output/process measures	F1.PSFR	F2.MI	F3.SIV	F4.OPCI	F5.CMRR	F6.WEICPC
1.The proportion of procedures in the health facility that require written consent for	1. Does this facility offer services for Theatre, laboratory, X-ray, Ultrasound.	1. Do you have a system to refer and transfer women to a higher level of care?	Is Caesarean Section delivery a signal EmOC function provided in this facility and was	Plan for delivery communicated mother (SVD/assisted vaginal delivery/C/S)?	Was it an emergency or elective C/S?	1.Were you told why the caesarean section was done?<br>2.Did they ask for you to agree

which there is an associated record of the woman's consent.	2. Does this facility refer patients to other facilities?	2. If yes, do you have guidelines for such referral?	it done in the past 6 months?			before the operation was done?
2.The proportion of all women undergoing examinations or procedures in the health facility who reported that their permission was sought before the examination or procedures were performed.		Does the training session cover patient's rights?	Does your facility have clear policies on rights of the patient?	Plan for delivery communicated to mother(SVD/assisted vaginal delivery/C/S)?		1.Were you told why the caesarean section was done?<br><br>2.Did they ask for you to agree before the operation was done?
Outcome measures	F1.PSFR	F2.MI	F3.SIV	F4.OPCI	F5.CMRR	F6.WEICPC
1.The proportion of all women who gave birth in the health facility	Does the facility have functional screen/curtain for privacy?	1. Does the training session cover patient's rights?	1. Does your facility have clear policies on rights of the patient?	1.Were women's privacy assured/maintained during interaction with		1.Were you worried that people were seeing you when you were being

who were satisfied with the degree of privacy during their stay in the labour and childbirth areas.		2. Facility specific management policy on respectful and dignified care for all.	2. Have you received training on how to treat childbearing women with compassion and dignity (customer care)?	the health worker?<br>2.Was the woman's privacy maintained during the examination?<br>3.During delivery, was privacy ensured?		examined by staff/health care workers?<br><br>2.Were you worried that people were hearing you when you were talking with the staff/health care workers?
2.The proportion of all women examined and treated in the health facility who expressed satisfaction with the degree of privacy during examinations and treatment.	Does the facility have functional screen/curtain for privacy?	1. Does the training session cover patient's rights?<br><br>2. Facility specific management policy on respectful and dignified care for all.	1. Does your facility have clear policies on rights of the patient?<br><br>2. Have you received training on how to treat childbearing women with compassion and dignity	1.Were women's privacy assured/maintained during interaction with the health worker?<br>2.Was the woman's privacy maintained during the examination?<br>3.During delivery, was		1.Were you worried that people were hearing you when you were talking with the staff/health care workers?<br><br>2.Were you worried that people were seeing you when you were being examined by

			(customer care)?	privacy ensured?		staff/health care workers?
3.The proportion of all women who gave birth in the health facility who expressed satisfaction with the health services.		1. Do facility training sessions cover patient's rights?<br><br>2. Facility specific management policy on respectful and dignified care for all	1. Is there a policy and procedure for addressing patient concerns in this facility?<br><br>2. Does your facility have clear policies on rights of the patient?<br><br>3. Have you received training on how to treat childbearing women with compassion and dignity (customer care)?			1.In general, how satisfied are you with the care you received during your delivery at this facility?
Quality statement5.2						

No woman or newborn is subjected to mistreatment, such as physical, sexual or verbal abuse, discrimination, neglect, detainment, extortion or denial of services.						
Input measures	F1.PSFR	F2.MI	F3.SIV	F4.OPCI	F5.CMRR	F6.WEICPC
1.The health facility has written, up-to-date, zero-tolerance non-discriminatory policies with regard to mistreatment of women and newborns.		1. Does the training session cover patient's rights?<br><br>2. Facility specific management policy on respectful and dignified care for all.	1.Is there a policy and procedure for addressing patient concerns in this facility?<br><br>2. Does your facility have clear policies on professional ethics?<br><br>3. Does your facility have clear policies on rights of the patients?	1. Did the woman request for anything and was not given?<br><br>2. Was woman told respectfully why the request was refused (if yes above)?		Were you subjected to any physical abuse/verbal abuse/sexual abuse/ other specify...during your stay at the facility?
2.The health facility has a system whereby the mothers of small, sick newborns can	Does this facility have a separate paediatric ward?	Does the facility have specific management policies on Rooming in?	1.Does this facility operate a static child welfare clinic?			

be close to and nurse their babies.		2. Does the facility have specific management policies on KMC for babies?	2. How many days of the week do you offer PNC services?			
3.The fee structures for maternity and newborn care are equitable, affordable and clearly displayed.		Does the facility have specific management policies on free emergency care, and free delivery care?<br><br>Do women have to pay for the use of ambulance?	1. Is a woman expected to pay a fee for a normal delivery?<br><br>2. If a woman is expected to pay a fee, how much do they usually pay?<br><br>3. Is a woman expected to pay a fee if she brings her newborn to the facility for care?<br><br>4. What is the estimated average cost of	1. Are women supposed to pay for maternity services?<br><br>2. Are families supposed to pay for sick newborn care?<br><br>3. Did the woman and her family pay any monies for the services provided in the facility?<br><br>4. How much did the woman pay in total for the services		1.Did you have to pay anything (cash or kind) for the service provided during labour or delivery?<br><br>2. How much did you have to pay in total for sleeping in the facility/supplies, drugs/ultrasoun d scan/laboratory investigations, food provided by the facility/delivery care, and other, specify…?

			care for sick newborns including admission costs if they were in the facility for 3 nights?	since entering the facility till discharge?<br><br>5. Did the patient or the family complain about any monies being charged?<br><br>6. Was any service or supply withheld due to inability to pay?		
4.The health facility has written accountability mechanisms for redress in the event of mistreatment.	Is there a complaint/suggestions box or other means of receiving anonymous feedback from clientele?	1.What happens if a unit or person is found not to be performing well?<br><br>2.Are there reward sanctions?	1.Is there a policy and procedure for addressing patient concerns in this facility?<br><br>2. Does your facility have clear policies on			Were you subjected to any physical abuse/verbal abuse/sexual abuse/ other specify…during your stay at the facility?

		3. Are women encouraged to report such abuses from health professionals to managers?<br><br>4. what measures did you take to prevent future occurrence of such abuse?	rights of the patient?			
5.The health facility has a written, up-to-date policy and protocols that outline women's and families' right to make a complaint about the care received and has an easily accessible	Is there a complaint/suggestion box or other means of receiving anonymous feedback from clientele?	1.What are the main policies of this facility regarding maternity and newborn care? collect any points.<br><br>2.Is there a complaint system?	1. there a policy and procedure for addressing patient concerns in this facility?<br><br>2. Does your facility have clear policies on rights of the patient?	1. Did the woman request for anything and was not given?<br><br>2. Was woman told respectfully why the request was refused (if yes above)?		Did they allow you ask questions when you wanted?<br><br>Do you think health workers were responsive when you ask for their support?

mechanism (e.g., a box) for handing in complaints.		3.Is there a person responsible for the complaints?				
6.Health care staff in the maternity unit receive in-service training and supportive supervision in respecting the rights of mothers and newborns, respectful care and accountability mechanisms. Orientation is provided for new staff.		1.Are there additional trainings provided to workers posted in maternity and children's wards?<br><br>2.Are there additional refresher training for the staff in maternity and children's wards?<br><br>3.Have there been training over the past year?	Have you received training on how to treat childbearing women with compassion and dignity (customer care)?	1. Did the woman request for anything and was not given?<br><br>2. Was woman told respectfully why the request was refused (if yes above)?		Were you subjected to any physical abuse/verbal abuse/sexual abuse/ other specify…during your stay at the facility?

		4.How often have they been trained over the past year?				
7.The health facility policy for payment specifically precludes detention of a woman or baby for non-payment.		Does the facility have specific management policies on free emergency care, and free delivery care?<br><br>Do women have to pay for the use of ambulance?	1. Is a woman expected to pay a fee for a normal delivery?<br><br>2. If a woman is expected to pay a fee, how much do they usually pay?<br><br>3. Is a woman expected to pay a fee if she brings her newborn to the facility for care?<br><br>4. What is the estimated average cost of care for sick newborns	1. Are women supposed to pay for maternity services?<br><br>2. Are families supposed to pay for sick newborn care?<br><br>3. How much did the woman pay in total for the services since entering the facility till discharge?<br><br>4. any service or supply withheld due to inability to pay?		1.Did you have to pay anything (cash or kind) for the service provided during labour or delivery?<br><br>2. How much did you have to pay in total for sleeping in the facility/supplies, drugs/ultrasoun d scan/laboratory investigations, food provided by the facility/delivery care, and other, specify...?

			including admission costs if they were in the facility for 3 nights?			
8.The health facility has a complaints box, which is easily accessible to women and their families, is periodically emptied and the contents reviewed.	Is there a complaint/suggestions box or other means of receiving anonymous feedback from clientele?	1.Is there a complaint system?<br><br>2.Is there a person responsible for the complaints?	1. Are community stakeholders involved in addressing disrespect and abuse of women during childbirth?<br><br>2. Is there a process for identifying and reporting abuse?	1.Did the woman request anything and was not given?<br><br>2.Was woman told respectfully why the request was refused (if yes above)?		1.Were you given the opportunity to ask questions or express your concern during the care?<br><br>2. Do you think health workers took your concerns into consideration in providing care during your stay there?
Output/process measures	F1.PSFR	F2.MI	F3.SIV	F4.OPCI	F5.CMRR	F6.WEICPC
1.The proportion of all women who		1.Is there a process for identifying	1. Are community stakeholders			Were you subjected to any physical abuse,

gave birth in the health facility who reported physical, verbal or sexual abuse, to themselves or their newborns, during labour or childbirth or after birth.		abuse of women and/ or children who seek care in your facility?<br><br>2. if yes, do you have a process f reporting these cases?<br><br>3. Are other staff encouraged to report abuses of clients by their colleagues (with protection of their identities) **Whistle-blowers?**<br><br>4. Are women encouraged to report such abuses from health professionals to managers?	involved in addressing disrespect and abuse of women during childbirth?<br><br>2. Is there a process for identifying and reporting abuse?<br><br>3. Have you encountered a victim of abuse during your practice?<br><br>4. Have you received training on how to treat childbearing women with compassion and dignity			verbal abuse, sexual abuse, other, specify…during your stay at the facility?

		5. if yes, can you show the most recent report of abuse?	(customer care)?			
2.The proportion of women who gave birth in the health facility who were satisfied that the facility met their religious and cultural needs.		1.Do the training for workers posted in maternity and children's ward cover respectful maternity care, abuse, patient's rights?<br><br>2. Does facility specific management policies include respectful and dignified care for all?	1.Does your facility have clear policies on rights of the patient?<br><br>2. Have you received training on how to treat childbearing women with compassion and dignity (customer care)?<br><br>3. Are community stakeholders involved in addressing disrespect and abuse of women			1.In general, how satisfied are you with the care you received during your delivery at this facility?<br><br>2. in general, what did you like?<br>3. what did you not like?

			during childbirth?			
3.The proportion of women who attended the health facility who were refused care because of their inability to pay.		Does the facility have specific management policies on free emergency care, and free delivery care?<br><br>Do women have to pay for the use of ambulance?	1. Is a woman expected to pay a fee for a normal delivery?<br><br>2. Is a woman expected to pay a fee if she brings her newborn to the facility for care?	1. Are women supposed to pay for maternity services?<br><br>2. Did the woman and her family pay any monies for the services provided in the facility?<br><br>3. Was any service or supply withheld due to inability to pay?		1.Did you have to pay anything (cash or kind) for the service provided during labour or delivery?
4.The proportion of women who gave birth in the health facility who were aware of the existence and location of	Is there a complaint/suggestions box or other means of receiving anonymous feedback from clientele?	1.Are women encouraged to report such abuses from health professionals to managers?	1.Is there a policy and procedure for addressing patient concerns in this facility?	1.Did the woman request anything and was not given?<br><br>2. was woman told respectfully why the request		Were you given the opportunity to ask questions or express your concern during the care?

a complaints box.		2. if yes, can you show the most recent report of abuse?<br><br>3. is there a complaint system?	2. Does your facility have clear policies on rights of the patient?<br><br>3. Are community stakeholders involved in addressing disrespect and abuse of women during childbirth?	was refused (if yes above)?		
Outcome measures	F1.PSFR	F2.MI	F3.SIV	F4.OPCI	F5.CMRR	F6.WEICPC
1.The proportion of all women who gave birth in the health facility who expressed satisfaction with the health services.		1. Do facility training sessions cover patient's rights?<br><br>2. Facility specific management policy on respectful and	1. Is there a policy and procedure for addressing patient concerns in this facility?<br><br>2. Does your facility have			1.In general, how satisfied are you with the care you received during your delivery at this facility?<br><br>2. overall, how satisfied were

		dignified care for all	clear policies on rights of the patient?<br><br>3. Have you received training on how to treat childbearing women with compassion and dignity (customer care)?			you with the hygiene standards at the health facility?
2.The proportion of all women who gave birth in the health facility who reported having been treated with respect and their dignity preserved.		1.Do the training of workers posted in maternity and children's ward cover respectful maternity care?/abuse/patient's rights?<br><br>2. Does the facility specific management	1.Does your facility have clear policies on rights of the patient?<br><br>2. Have you received training on how to treat childbearing women with compassion and dignity	Were women's privacy ensured/maintained during interaction with the health worker?		1.Do you think the health workers treated you with respect?<br><br>2. Do you think the health workers were responsive when you ask for their support?

		policies include respectful and dignified care for all?	(customer care)?<br><br>3. Are community stakeholders involved in addressing disrespect and abuse of women during childbirth?			
3.The proportion of all women in the health facility who made a complaint whose complaints were acted upon without repercussions.	Is there a complaint/suggestion box or other means of receiving anonymous feedback from clientele?	1. are women encouraged to report such abuses from health professionals to managers?<br>2. if yes, can you show the most recent report of abuse?<br>3. How did you respond to the most recent case of abuse?	1.Does your facility have clear policies on professional ethics?<br><br>2. Have you ever encountered a victim of abuse during your practice?<br><br>3. If yes, what help did you offer the victim?			1.Were you subjected to any physical abuse/verbal abuse/sexual abuse/other, specify…during your stay at the facility?<br><br>2.Do you think the health workers were responsive when you ask

		4. what measures did you take to prevent future occurrence of such abuse?	4. Can you mention at least two preventive measures that are implemented in your facility against abuses?			for their support?
Quality statement 5.3						
All women can make informed choices about the services they receive, and the reasons for interventions or outcomes are clearly explained.						
Input measure	F1.PSFR	F2.MI	F3.SIV	F4.OPCI	F5.CMRR	F6.WEICPC
1.The health facility has a written, up-to-date policy for obtaining informed consent from women before examinations and procedures.		1.What are the main policies of this facility regarding maternity and newborn care? collect any points.<br><br>2. Does the training for workers posted in maternity and children's ward sessions	Does the facility have clear policies on rights of the patient?	Plan for delivery communicated to mother (SVD/Assisted vaginal delivery/C/S)		1.Were you told why the caesarean section was done?<br><br>2. Did they ask for you to agree before the operation was done?<br><br>3. were you told your BP was high?

		cover patient's rights?				
2.The health facility has a standard informed consent form that helps health care staff to provide easily understandable information to women in order to obtain their fully informed consent.		1.What are the main policies of this facility regarding maternity and newborn care? collect any points.<br><br>2. Does the training for workers posted in maternity and children's ward sessions cover patient's rights?	Does the facility have clear policies on rights of the patient?	Plan for delivery communicated to mother (SVD/Assisted vaginal delivery/C/S)		1.Were you told why the caesarean section was done?<br><br>2. Did they ask for you to agree before the operation was done?
3.Health care staff in the health facility receive in-service training and supportive supervision in effective informed		1. Are there additional trainings provided to workers posted in maternity and children's wards?	1.Have you received refresher/in-service training in the past 12 months?<br><br>2. Have you received	1. Plan for delivery communicated to mother (SVD/Assisted vaginal delivery/C/S)		1.Were you told why the caesarean section was done?<br><br>2. Did they ask for you to agree before the

consent procedures and in women's right to choose care at childbirth. Orientation is provided for new staff.		2. How often have they been trained over the past year?<br><br>3. Does trainings for staff posted in maternity and children's ward cover patient's rights?	training on how to treat childbearing women with compassion and dignity (customer care)?	2. Woman asked for her preferred delivery position?		operation was done?
4.The health facility has written accountability mechanisms for redress in the event that women are denied informed choice, and the mechanism is displayed.	Is there a complaint/sugg estions box or other means of receiving anonymous feedback from clientele?	1.What happens if a unit or person is found not be performing well? Are there reward sanctions?<br><br>2.Is there a complaint system?<br><br>3.Is there a person	1. Is there a policy and procedure for addressing patient concerns in this facility?<br><br>2. Does your facility have clear policies on rights of the patient?	1. Did the woman request anything and was not given?<br><br>2. Was woman told respectfully why the request was refused (if yes above)?		Were you told why the caesarean section was done?<br><br>Did they allow you to ask questions when you wanted?<br><br>Do you think that the health workers were responsive when you ask

		responsible for the complaints?				for their support?
Output/process measures	F1.PSFR	F2.MI	F3.SIV	F4.OPCI	F5.CMRR	F6.WEICPC
1.The proportion of procedures in the health facility that require written consent for which there is an associated record of consent signed by the woman or a family member.	Does this facility admit patients over night?<br><br>Does this facility provide laboratory, Ultrasound, X-ray, Theatre services?		1. Was blood transfusion services provided as a signal EmOC function and done in the past 6 months?<br><br>2. Was caesarean section delivery provided as a signal EmOC function and done in the past 6 months?	Plan for delivery communicated to mother(SVD/Assisted vaginal delivery/C/S)?	1.What type of delivery did she have?<br>2. Was it an emergency or elective C/S?<br>3. Did they mention what was done for the baby?	1.Were you told why the caesarean section was done?<br>2.Did they ask you to agree before the operation was done?
2.The proportion of all women who gave birth in the health facility who felt	Does this facility provide laboratory, Ultrasound, X-ray, Theatre services?		1. Is there a policy and procedure for addressing patient	Feedback to mother after examination		1. Were you told why the caesarean section was done?

adequately informed by health care staff regarding decisions taken about their care.			concerns in this facility?<br><br>2. Does your facility have a clear policies on rights of the patient?			2. Did they ask you to agree before the operation was done?<br><br>3. Did they tell you what they found before sending you to labour care?
3. The proportion of women who received care in the health facility who were aware that they had the right to accept or refuse treatment.		1. Do the training sessions cover patient's rights?<br><br>2. Facility specific management policy on respectful and dignified care for all.	1. Does your facility have clear policies on rights of the patient?<br><br>2. Have you received training on how to treat childbearing women with compassion and dignity (customer care)?			

Outcome measures	F1.PSFR	F2.MI	F3.SIV	F4.OPCI	F5.CMRR	F6.WEICPC
1.The proportion of women who gave birth in the health facility by caesarean section who were aware of the reason for the caesarean section.			1. Was caesarean section delivery provided as a signal EmOC function and done in the past 6 months?<br><br>Vignettes:<br>2. Plan for delivery within the next 24hrs<br><br>3. Prepare for Caesarean section	Plan for delivery communicated to mother (SVD/Assisted vaginal delivery/C/S)	Was it an emergency or elective caesarean section?	Were you told why the caesarean section was done?
2.The proportion of all women who gave birth in the health facility who expressed satisfaction		1. Do facility training sessions cover patient's rights?<br><br>2. Facility specific management	1. Is there a policy and procedure for addressing patient concerns in this facility?			1.In general, how satisfied are you with the care you received during your delivery at this facility?

with the health services.		policy on respectful and dignified care for all	2. Does your facility have clear policies on rights of the patient?<br><br>3. Have you received training on how to treat childbearing women with compassion and dignity (customer care)?			2.Are you satisfied with the level of attention and care given to your newborn baby after delivery?
3.The proportion of women who gave birth in the health facility who felt they had shared decisions about their labour, birth and postnatal care.		1.Does the facility receive feedback on the care provided to clients?<br><br>2. Does the facility specific management policies include respectful and		Plan for delivery communicated to mother(SVD/assisted vaginal delivery/C/S).		1.Did they tell you what they found before sending you to labour care?<br><br>2.Were you given the opportunity to ask questions or express your

		dignified care for all?<br><br>3. Does the training for workers posted in maternity and children's ward include sessions on abuse and patient's rights?				concern during the care?
Standard 6: Every woman and her family are provided with emotional support that is sensitive to their needs and strengthens the woman's capability.						
Quality statement 6.1						
Every woman is offered the option to experience labour and childbirth with the companion of her choice.						
Input measures	F1.PSFR	F2.MI	F3.SIV	F4.OPCI	F5.CMRR	F6.WEICPC
1.The labour and childbirth areas are organized in such a way as to allow a physical private space for the woman and her companion	Does the facility have functioning screen/curtain for privacy?<br><br>What is the total bed capacity?	1.Does the facility specific management policies include respectful and dignified care for all?<br><br>2.Does the facility specific		1.Were women's privacy ensured/maintained during interaction with the health worker?<br><br>2. Was the woman's		1.Were you allowed companion with you in the labour ward during labour? Companion means husband or family

at the time of birth.		management policies include partner/family involvement in maternity and newborn care?		privacy maintained during examinations?<br><br>3. How is the layout of the labour ward?<br><br>4.How is the layout of the delivery ward?<br><br>5.How many beds are in labour ward?		member or friend).<br><br>2. Was a companion allowed to be with you during counselling on the care of the baby?<br><br>3. Was the companion called to be with you during counselling on the care of the baby?
2.The health facility has a written, up-to-date protocol, which is explained to women and their families, to encourage all women to have		1.Does the facility specific management policies include respectful and dignified care for all?<br><br>2.Does the facility specific		1.Was any companion allowed to be present during delivery?<br><br>How is the layout of the labour ward?		1.Were you allowed companion with you in the labour ward during labour? Companion means husband or family

at least one person of their choice, as culturally appropriate, with them during labour, childbirth and the immediate postnatal period.		management policies include partner/family involvement in maternity and newborn care?		2.How is the layout of the delivery ward?		member or friend).<br><br>2. Was a companion allowed to be with you during counselling on the care of the baby?<br><br>3. Was the companion called to be with you during counselling on the care of the baby?
3.Health care staff in the health facility are oriented and receive in-service refresher training sessions at least once every 12 months on the		1.Are there additional trainings provided to workers posted in maternity and children's wards?	1. Have you received training on how to treat childbearing women with compassion and dignity (customer care)?	Was any companion allowed to be present during delivery?		1. Were you allowed companion with you in the labour ward during labour? Companion means husband or family

evidence for and positive impact of the presence of a chosen companion during labour and birth.		2.Are there additional refresher training for the staff in maternity and children's wards?<br><br>3.Have there been training over the past year?<br><br>4.How often have they been trained over the past year?<br><br>4.Does trainings for staff posted in maternity and children's ward cover patient's rights?	2. Does your facility have clear policies on rights of the patients?			member or friend).<br><br>2. Was a companion allowed to be with you during counselling on the care of the baby?<br><br>3. Was the companion called to be with you during counselling on the care of the baby?
4. Orientation sessions and information		What additional training is provided to	1. Does your facility have clear policies on	Was any companion allowed to be		1. Were you allowed companion with

(written or pictorial) are available to orient the companion on his or her role in supporting the woman during labour and birth.		workers posted in maternity ward?<br><br>Are there additional refresher training for staff?	rights of the patient?<br><br>Vignette:<br>2. Have somebody stay with her all the time in case she starts having seizures	present during delivery?		you in the labour ward during labour? Companion means husband or family member or friend).<br><br>2. Was a companion allowed to be with you during counselling on the care of the baby?<br><br>3. Was the companion called to be with you during counselling on the care of the baby?
Output/process measures	F1.PSFR	F2.MI	F3.SIV	F4.OPCI	F5.CMRR	F6.WEICPC

1.The proportion of all women who gave birth in the health facility who had a companion of their choice during labour and childbirth.		1.Does the facility specific management policies include respectful and dignified care for all?<br><br>2.Does the facility specific management policies include partner/family involvement in maternity and newborn care?		Was any companion allowed to be present during delivery?		1. Were you allowed companion with you in the labour ward during labour? Companion means husband or family member or friend).<br><br>2. Was a companion allowed to be with you during counselling on the care of the baby?<br><br>3. Was the companion called to be with you during counselling on the care of the baby?

Outcome measures	F1.PSFR	F2.MI	F3.SIV	F4.OPCI	F5.CMRR	F6.WEICPC
1.The proportion of all women who gave birth in the health facility who expressed satisfaction with the health services.		1. Do facility training sessions cover patient's rights?<br><br>2. Facility specific management policy on respectful and dignified care for all	1. Is there a policy and procedure for addressing patient concerns in this facility?<br><br>2. Does your facility have clear policies on rights of the patient?<br><br>3. Have you received training on how to treat childbearing women with compassion and dignity (customer care)?	Did the woman request for anything and was not given?<br><br>Was woman told respectfully why the request was refused (if yes above)?		1. In general, how satisfied are you with the care you received during your delivery at this facility?<br><br>2. Overall, how satisfied were you with the hygiene standards at the health facility?
Quality statement 6.2						

Every woman receives support to strengthen her capability during childbirth.						
Input measures	F1.PSFR	F2.MI	F3.SIV	F4.OPCI	F5.CMRR	F6.WEICPC
1.Health care staff in the labour and childbirth areas of the maternity unit were oriented in non-pharmacological and pharmacological pain relief and received in-service training or sessions at least once in the preceding 12 months.	Does the facility provide incinerator/Autoclave for sterilization/Uristix/Dipstick//glucometer/IV Canula/IV stand/Freezer/incubator/radiant warmer… Essential drugs: IV antibiotics/Magnesium Sulphate (MgS04), IV Diazepam, Oxytocic/Dexamethasone/Betamethasone (parenteral)/Ampicillin/Penicillin for IV/Gentamicin for IV.	1.What additional training is provided to workers posted in maternity and children's wards?<br><br>2. Are there additional refresher training for the staff?<br><br>3. have there been training over the past year?<br><br>4. How often have they been trained over the past year?	Have you received refresher/in-service training in the past 12 months?<br><br>Have you received refresher/in-service training in the past 12 months on essential care during labour and childbirth?	Did the woman have an IV-line access?	1.What was done with the woman with an obstetric complication?<br><br>2. Did the woman receive any antibiotic?<br><br>3. What antibiotic was given?<br><br>4. If women received IV fluid, which fluids were given?<br><br>5. If woman HIV positive, did the newborn receive PMTCT of HIV treatment?	Were you given any IV flued/medicine to take/injection(IM) and pain relief?

2.The health facility has a written, up-to-date protocol, which is explained to women and their families, to minimize unnecessary interventions, support normal labour and strengthen the woman's capability, so that she feels in control of her childbirth experience.		What are the main policies of this facility regarding maternity and newborn care? collect any points.	1.Is there a policy and procedure for addressing patient concerns in this facility?<br><br>2. Does your facility have clear policies on rights of the patient?			
3.Health care staff in the labour and childbirth areas of the maternity unit were oriented and received in-		1.What additional training is provided to workers posted in maternity and children's ward?	1.Have you received refresher/in-service training in the past 12 months?			

service training or refresher sessions at least once in the preceding 12 months to strengthen their interpersonal and cultural competence in providing emotional support.		2. Are there additional refresher training for the staff?<br><br>3. Have there been training over the past year?<br>4. How often have they been trained over the past year?	2. Have you received training on how to treat childbearing women with compassion and dignity (customer care)?			
4.The health facility has a referral mechanism for women and families with complex emotional needs, and refers them for specialist care.	1.Does this facility refer patients to other facilities?<br><br>2. Does this facility have an ambulance?	1.Do you have a system to refer and transfer a woman to a higher level of care?<br><br>2.If yes, do you have guidelines for such referral?	1.Have you ever have to refer a mother to another facility?<br><br>2. Have you ever have to refer a newborn to another facility?<br><br>3. In your practice, what do you do to	1.Does the facility have ambulance access to reception?<br><br>2. Was woman referred from another facility to this one?	What was done for the woman with an obstetric complication? (Referred to another facility/admitte d and managed/not recorded)	Did you plan during pregnancy to deliver in this facility? (i. yes, always attended ANC here; ii. yes, because I like it here, iii. No was referred from another facility, iv. No,

			ensure safe and timely referrals?<br><br>4. How can you ensure that newborn is warm during referral?			was brought in an emergency.
Output/process measures	F1.PSFR	F2.MI	F3.SIV	F4.OPCI	F5.CMRR	F6.WEICPC
1.The proportion of all women who gave birth in the health facility who did so in the labour position of their choice.				Woman asked for her preferred delivery position.		
2.The proportion of all women undergoing bereavement or an adverse outcome who received				Mothers who lost their babies referred to social workers for psycho social support.		Do you think health workers took your concerns into consideration in providing care during your stay there?

additional emotional support from health facility staff.						
Outcome measures	Fom1	F2.MI	F3.SIV	F4.OPCI	F5.CMRR	F6.WEICPC
1.The proportion of all women who gave birth in the health facility who expressed satisfaction with the health services.		1. Do facility training sessions cover patient's rights?<br><br>2. Facility specific management policy on respectful and dignified care for all	1. Is there a policy and procedure for addressing patient concerns in this facility?<br><br>2. Does your facility have clear policies on rights of the patient?<br><br>3. Have you received training on how to treat childbearing women with			1. In general, how satisfied are you with the care you received during your delivery at this facility?<br><br>2. Overall, were you satisfied with the hygiene standards at the health facility?

			compassion and dignity (customer care)?			
2.The proportion of all women who gave birth in the health facility who would recommend childbirth in that facility.						Would you recommend this health facility to a relative or friend for them to deliver?
3.The proportion of all women who gave birth in the health facility who reported a positive birth experience.						1. Do you think health care workers took your concerns into consideration in providing care during your stay here?<br><br>2. Are you satisfied about the attitude of the health workers?

						3. in general what did you like?<br>4. what did you not like?
4.The proportion of all women who gave birth in the health facility who were satisfied that their choices and preferences were respected.						1.Do you think health workers took your concerns into consideration in providing care during your stay there?<br><br>2. Do you think health workers treated you with respect?
Standard 7: For every woman and newborn, competent, motivated staff are consistently available to provide routine care and manage complications.						
Quality statement 7.1						
Every woman and child has access at all times to at least one skilled birth attendant and to support staff for routine care and management of complications.						
Input measures	F1.PSFR	F2.MI	F3.SIV	F4.OPCI	F5.CMRR	F6.WEICPC

1.The health facility has skilled birth attendants available at all times, in sufficient numbers to meet the anticipated work load.		1.Does the maternity department have a staffing plan or duty roster?<br>2. If yes, can you describe the staffing plan?<br>3.If yes, does staffing plan includes the number and categories of staff needed per shift?<br>4.Is the maternity staffing plan based on the work load, e.g., nurse to patient ratio?<br>5.If the staffing levels do not meet the needs of the maternity or newborn	1. Have you received formal training in the care of pregnant women?<br>2. Have you received formal training in the care of newborn babies?<br>3. Have you been trained in identifying newborn health complications?<br>4. Does this facility offer 24hr services?<br>5. Is this facility open to offer services on all weekdays?	1.How was the initial reception of the health worker to patients/client?<br><br>2. Did the health worker wash the hands before examination?<br><br>3. Did the health worker set an IV line on woman?	1.Who performed the surgery?<br><br>2. Did the surgeon specify fluid management of the woman after C/S?	Did the health worker do any of the following before sending you to the labour ward?<br>I. Check your eye lids, tongue or nails;<br>ii. check your BP<br>iii. take your urine and checked<br>iv. checked whether you were bleeding?<br><br>2. Did a health worker examine your baby after birth?

		wards, what actions do you take?				
2.The health facility has written up to date staffing policy, listing the numbers, types and competence of staff, that is reviewed continuously according to the work load.		1.What are the main policies of this facility regarding maternity and newborn care? collect any points<br><br>2. Does the maternity department have a staffing plan or duty roster?<br><br>3. if yes, staffing plan included the number and categories of staff needed per shift?<br><br>4. is the maternity	1. What is your designation (professional qualification)<br><br>2. In which department or unit do you work?<br><br>3. What is your position or rank<br><br>4. Have you received formal training in the care of pregnant women?<br><br>5. Have you had formal training in the management of sick or low	1. What are minimum qualification to work in the children's and maternity ward?<br><br>2. Does the training session cover management of pregnancy complications, management of birth complications/quality care/newborn resuscitation/essential newborn care	Who performed the surgery? Obstetrician/Gynaecologist/General surgeon/general practitioner/non physician clinician (health officer/clinical officer)	

		staffing plan based on workload, e.g. nurse to patient ratio?<br><br>5. if the staffing levels do not meet the needs of the maternity or newborn wards, what actions do you take?	birthweight newborns?			
3.The health facility has a roster that is displayed in all areas, giving the names of staff on duty, the times of their shifts and their specific roles and responsibilities.		1. Does the maternity department have a staffing plan or duty roster?<br><br>2. If yes, can you describe the staffing plan?<br><br>3. If yes, does staffing plan includes the				

		number and categories of staff needed per shift?<br><br>4.Is the maternity staffing plan based on the work load, e.g., nurse to patient ratio?				
4.The health facility has a written, up-to-date policy on triage and waiting times for emergency and non-emergency consultations and treatment.		1.What are the main policies of this facility regarding maternity and newborn care? collect any points.<br><br>2.Does the facility trainings to staff posted in maternity and children's wards cover triage,	Have you received refresher/in-service training in the past 12 months on Obstetric triage/initial assessment?			How long did you have to wait at the facility before you were first seen by a health care professional when you first came in for this delivery?

		management of pregnancy complications, management of birth complications, essential newborn care, and patient's rights?				
5.The health facility has clear communication channels to reach staff on duty at all times.	Does the facility have a functioning landline/mobile phone/radio phone?	1.What means of functional communication are available in the facility for staff to contact higher level referral facilities during referral for support?<br><br>2. What type of follow up postnatal visits are done? (i. physically present for visit; ii. Phone				

		contact; iii. 9=N/A, not done<br><br>3. if by telephone contact, do they keep phone contacts of the all clients who come to the facility?				
Output/process measures	F1.PSFR	F2.MI	F3.SIV	F4.OPCI	F5.CMRR	F6.WEICPC
1.The proportion of available posts in the health facility that were filled by staff with the necessary competence for the job description to allow the facility to	1.How many professional neonatologist, obstetrician/Gynaecologist, Paediatricians, Medical Officers, Midwives, Enrolled nurse midwives, other nurses, others, specify, do you	1.What is the minimum qualifications to work in the children's ward?<br><br>2. What is the minimum qualifications to work in the maternity ward?	Have you received refresher/in-service training in the past 12 months?<br><br>Does this facility offer 24hr services?			

provide 24-h service.	have in this facility?<br>2. How many are in the maternity?	3. are there additional refresher training for the staff?				
2.The proportion of staff who have been oriented to their functions, roles and responsibilities in the facility or unit to which they are assigned.		1.What additional training is provided to workers posted in maternity and children's wards?<br><br>2.Are there additional refresher training for the staff in maternity and children's ward?	Have you received refresher/in-service training in the past 12 months?			
3.The proportion of women who attended the health facility who reported receiving			Have you received refresher/in-service training in the past 12 months on Obstetric			1. How long did you have to wait at the facility before you were first seen by the health care professional

attention within the appropriate time for their condition as per facility policy on triage and waiting time			triage/initial assessment?			when you first came in for this delivery?<br><br>2.How long did you have to wait at the labour ward before you were first seen by a health professional when you first came in for this delivery?
Outcome measures	F1.PSFR	F2.MI	F3.SIV	F4.OPCI	F5.CMRR	F6.WEICPC
1.The proportion of women and their newborns in the health facility who were attended by a skilled birth attendant (as per the definition)		What is the minimum qualifications to work in the children's ward<br><br>What is the minimum qualifications to work in the maternity ward		1.Time of first professional Health worker contact<br><br>2. Was the woman examined in the labour ward	Who performed the surgery?	1. How long did you have to wait at the facility before you were first seen by the health care professional when you first came in for this delivery?

during and after childbirth.				3. Did the birth attendant wear protective clothing?<br><br>4. Birth attendant washes hands with soap and water?		2. Was the baby examined from head to toe before discharge?<br><br>3. Did health workers examine you at any time after birth?
2.The proportion of all women who gave birth at the health facility who reported having been informed about danger signs for her and her baby and emergency preparedness.				1. Was the woman counselled on maternal dangers signs?<br><br>2. Was the woman counselled on newborn danger signs?	Was the woman counselled on maternal danger signs/newborn danger signs/care seeking for illness/postpartum care?	1. Were you told about danger signs after childbirth for the mother- when you have to seek care again at the health facility?<br><br>2.Were you told about danger signs after childbirth for the newborn- when you have to seek

						immediate care at the nearest health facility?
3.The proportion of women who attended the health facility who were satisfied with the health care they received.		1. Do facility training sessions cover patient's rights?<br><br>2. Facility specific management policy on respectful and dignified care for all	1. Is there a policy and procedure for addressing patient concerns in this facility?<br><br>2. Does your facility have clear policies on rights of the patient?<br><br>3. Have you received training on how to treat childbearing women with compassion and dignity (customer care)?			In general, how satisfied are you with the care you received during your delivery at this facility?

Quality statement 7.2						
The skilled birth attendants and support staff have appropriate competence and skills mix to meet the requirements of labour, childbirth and the early postnatal period.						
Input measures	F1.PSFR	F2.MI	F3.SIV	F4.OPCI	F5.CMRR	F6.WEICPC
1.The health facility has a programme for continuing professional development and skills development for all skilled birth attendants and other support staff and conducts regular training.		1.What additional training is provided to workers posted in maternity and children's wards?<br>2.Are there additional refresher training for the staff in maternity and children's ward?<br>3.Have there been training over the past year?<br>4.How often have they been trained over the past year?	1.Have you received refresher/in-service training in the past 12 months?<br><br>2.Have you received training on how to treat childbearing women with compassion and dignity (customer care)?			

2.The health facility has standard procedures and plans for recruitment, deployment, motivation (recognition and reward scheme) and retention of all staff.		1.How are staff recruited into the maternity wards?<br>2. How are staff recruited into the children's ward?<br><br>3. what happens if a unit or person is performing exceptionally well? Are there reward system?<br><br>4. what happens if a unit or person is found not to be performing well? Are there reward sanctions?				
3.The health facility periodically		1.How are staff recruited in				

appraises all staff and has a mechanism for recognizing good performance.		maternity wards?<br>2.What are the minimum qualifications to work in the maternity wards?<br>3. what happens if a unit or person is performing exceptionally well? Are there reward system?				
4.The health facility has sufficient numbers of educated, competent, licensed, motivated, regulated skilled birth attendants with an appropriate	1.How many professionals of neonatologist, obstetrician/Gynaecologist, Paediatricians, Medical Officers, Midwives, Enrolled nurse midwives, other nurses, others,	1. What are the minimum qualifications to work in the maternity wards?<br>2. What are the minimum qualification to work in the children's ward?	1.What is your highest level of education?<br>2.What is your designation (professional qualification)?<br>3.In which department/unit do you work?<br>4.What is your position/rank?	1.How many staff were on duty?<br><br>2. Who conducted the delivery?	1.Who performed the surgery?<br><br>2. Did the surgeon specify the fluid management of the woman after the C/S?	Did the health worker examine your baby after birth?

skills mix, working in multidisciplinary teams.	specify, do you have in this facility?<br>2. How many are in the maternity?	3. What additional trainings is provided to workers posted in maternity and children's wards?				
5.The health facility provides an enabling, supportive environment for professional staff development, with regular supportive supervision and mentoring.		1. QI mentoring/coaching.<br><br>2. Do you receive external supervisory visits to this facility?<br><br>3. How often do you receive these external visits?	Have you received refresher/in-service training in the past 12 months?			
6.The health facility facilitates inter-professional collaborative practice, with		1.Does the maternity department have a staffing plan or duty roster?	Have you received refresher/in-service training in the past 12 months?			

clear roles and responsibilities based on the professional scope of practice and care needs during labour, childbirth and the early postnatal period.		2. if yes, staffing plan included the number and categories of staff needed per shift?<br>3. is the maternity staffing plan based on workload e.g., nurse to patient ratio?				
Output/process measures	F1.PSFR	F2.MI	F3.SIV	F4.OPCI	F5.CMRR	F6.WEICPC
1. The proportion of skilled birth staff at the health facility who received a written job description on deployment to the facility.	How many professionals do you have in this facility? How many are in maternity?	1. Minimum qualification to work in the maternity wards?<br><br>2. What are the minimum qualification to work in the children's wards?		1.How many staff are on duty?<br><br>2. who conducted the delivery?	Who performed the surgery?	

		3. Does the facility have specific policy on task shifting?<br><br>4. At what level do you have focal persons for infection prevention and control?				
2.The proportion of skilled birth attendants at the health facility who received in-service training, a refresher session or mentoring within the past 12 months.		Does your facility have a quality improvement programme, which includes Quality improvement mentoring/coac hing?	Have you received refresher/in-service training in the in the past 12 months?			
3.The number of supervisory visits to support		1.Do you receive external supervisory				

clinical competence and performance improvement (in the past three months).		visits to this facility?<br>2.How often do you receive these external visits?				
4.The proportion of staff at the health facility who were assessed at least once in the preceding 12 months.		How does the facility monitor staff performance?	Vignettes:<br>1. Which of the signal EmOC functions does this facility provide and which of those was done in the past 6months?<br><br>2. Which of the signal newborn functions does this facility provide and which of those was done in the past 6months?			
5.The number of team meetings held per month to review		1.Does your facility have a quality improvement				

competence and quality improvement activities.		programme which includes<br>i) quality improvement teams.<br>ii) QI action plans<br>iii)QI mentoring/coaching<br>iv) capturing and use of data<br>v) monitoring and evaluation				
6.The number of interactions per month with professional mentors to ensure clinical competence and improve performance.		1.Does your facility have a quality improvement programme which includes quality improvement teams?<br>2. Does your facility have a quality improvement programme	Have you received refresher/in-service training in the past 12 months?			

		which includes quality improvement action plans?				
7.The proportion of all staff at the health facility who were engaged in at least two active quality improvement team meetings and participated in quality improvement activities in the preceding six months.	1.Does this facility routinely conduct audit for maternal deaths?<br>2. Does this facility routinely conducts audit for early neonatal deaths?<br>3. Does this facility routinely conduct audit for stillbirths?	Does your facility have a quality improvement programme, which includes i)quality improvement teams?<br>ii) QI action plans<br>iii) monitoring and evaluation	Have you received refresher/in-service training in the past 12 months?			
Outcome measures	F1.PSFR	F2.MI	F3.SIV	F4.OPCI	F5.CMRR	F6.WEICPC
1.The proportion of all women who		1. Do facility training	1. Is there a policy and procedure for			1.In general, how satisfied are you with the

gave birth at the health facility who were satisfied with the care and support from facility staff.		sessions cover patient's rights?<br><br>2. Facility specific management policy on respectful and dignified care for all	addressing patient concerns in this facility?<br><br>2. Does your facility have clear policies on rights of the patient?<br><br>3. Have you received training on how to treat childbearing women with compassion and dignity (customer care)?			care you received during your delivery at this facility?<br><br>2.Are you satisfied with the level of attention and care given to your newborn baby after delivery?<br><br>3.Do you think the health workers were responsive when you ask for their support.
2. The proportion of skilled birth attendants and support staff at the health facility whose		1. How does the facility monitor staff performance?<br><br>2. What happens if a				

preceding performance appraisal was satisfactory.		unit or person is performing exceptionally well? are there rewards system?<br><br>3. What happens if a unit or person is found not to be performing well? are there reward sanctions?				
4.The proportion of all staff at the health facility who could identify and report on at least one clinical improvement activity in which they were personally	1. Does this facility routinely conduct audit for maternal deaths?<br><br>2. Does this facility routinely conduct audit for early neonatal deaths?	1. Have you used the data to make programme and management decisions?<br><br>2. If yes, please describe the most recent example for maternal and newborn care.				

involved in the past six months.	3. Does this facility routinely conduct audit for stillbirths?	3. Does your facility have a quality improvement programme, which includes i) quality improvement teams; ii) QI action plans; iii) QI mentoring/coaching; iv) Monitoring and evaluation				
5.The proportion of all staff at the health facility who were actively considering looking for a new job.		1.What happens if a specially – trained staff working on maternity/newborn units apply for a higher post in another department?				
Quality statement 7.3						
Every health facility has managerial and clinical leadership that is collectively responsible for developing and implementing appropriate policies and fosters an environment that supports facility staff in continuous quality improvement.						

Input measures	F1. PSFR	F2.MI	F3.SIV	F4.OPCI	F5.CMRR	F6.WEICPC
1.The health facility has a written, up-to-date plan for improving the quality of care and a patient safety programme.	1. Does this facility routinely conduct audit for maternal deaths?<br><br>2. Does this facility routinely conduct audit for early neonatal deaths?<br><br>3. Does this facility routinely conduct audit for stillbirths?	1.Does the facility have quality improvement programme?<br>2.Does the facility have Quality improvement teams?<br><br>3.Does the facility have Quality improvement action plans?<br>4. Does the facility have Quality improvement mentoring/coaching?<br>5. Does the facility have Capturing and use of data?	In your practice, what do you do to ensure safe and timely referrals?<br><br>Does your facility have clear policies on rights of the patient?			

		6. Does the facility have monitoring and evaluation?				
2.The health facility has a written, up to date leadership structure, with defined roles and responsibilities and lines of accountability for reporting.	How many of the following professionals do you have in this facility? How many are in maternity? (Neonatologist/Obstetrician/Gynaecologist/Paediatricians/Medical Officers/Midwives/Enrolled nurse midwives/other nurses/Other, specify	1.Does the maternity department have a staffing plan or duty roster?<br><br>2.If yes, can you describe the staffing plan?<br><br>3.If yes, does the staffing plan includes the number and categories of staff needed per shift?<br><br>4.Is the maternity staffing plan based on workload, e.g.,				

		nurse patient ratio?				
3.The health facility has a designated quality improvement team and responsible personnel.	1. Does this facility routinely conduct audit for maternal deaths?<br><br>2. Does this facility routinely conduct audit for early neonatal deaths?<br><br>3. Does this facility routinely conduct audit for stillbirths?	1.Does the facility have a quality improvement programme?<br><br>2.Does the facility have quality improvement teams?<br><br>3. Does the facility have quality improvement action plans, quality improvement mentoring/coaching, capturing and use of data, monitoring and evaluation.				

4.The health facility has a mechanism for regular collection of information on patient and provider satisfaction.	Is there a complaint/suggestions box or other means of receiving anonymous feedback from clientele?	1. Facility specific management policy on routine review monitoring of care quality.<br><br>2. Facility specific management policy on strict maintenance of data collection system and use	Routinely follow up mothers and newborns after discharge from the facility?	1.Woman's pregnancy records reviewed (if available)?<br><br>2.History taken<br><br>3. how was the initial reception of the health worker to patient/client<br><br>4. communication between health worker and client	1.Is/are birth weight recorded for all babies?<br><br>2.How many times was the women's temperature checked and recorded?<br><br>3. How many times was blood pressure checked and recorded between first exam and delivery?<br><br>4. How many times was mother's pulse checked and recorded between first exam and delivery?	

					5. Was the fetal heart rate recorded?<br><br>6. How many times was a vaginal examination carried out and recorded between first exam and delivery?<br><br>7. Was the state of the membranes and liquor recorded?	
5.The health facility holds at least one monthly meeting to review data, monitor quality improvement performance,	1. Does this facility routinely conduct audit for maternal deaths?<br><br>2. Does this facility	1.Does the facility have quality improvement programme?<br><br>2. Does the facility have Quality	Routinely follow up mothers and newborns after discharge from the facility?			

make recommendations to address any identified problems, honour those who have performed well and encourage staff who are struggling to improve.	routinely conduct audit for early neonatal deaths?<br><br>3. Does this facility routinely conduct audit for stillbirths?	improvement mentoring/coaching?<br><br>5. Does the facility have Capturing and use of data? Monitoring and evaluation?<br><br>6. Does the facility have minutes of management meetings?				
6.All standard governing procedures (policies and protocols) are in place and accessible to all relevant staff.		1.what are the main policies of this facility regarding maternity and newborn care? collect any point	1.Is there a policy and procedure for addressing patient concerns in this facility?<br><br>2. Does your facility have clear policies on			

			professional ethics?<br><br>3. Does your facility have clear policies on rights of the patient?<br><br>4. Is there IPC policy available in your facility?			
7.The proportion of all health facility leaders who were trained in quality improvement and leading change (use of information, enabling behaviour, continuous learning).	1. Does this facility routinely conduct audit for maternal deaths?<br><br>2. Does this facility routinely conduct audit for early neonatal deaths?	Does the training sessions for staff posted in maternity and children's ward cover quality of care, triage, essential newborn care, professional ethics, patient's rights?	Have you received refresher/in-service training in the past 12 months?			

	3. Does this facility routinely conduct audit for stillbirths?					
8.The health facility holds at least two annual meetings with stakeholders (e.g., the community, service users, partners) to review its performance, identify problems and make recommendations for joint actions to improve quality.		1. Are community stakeholders involved in quality improvement efforts in your facility?<br><br>2. If yes, briefly describe how they are involved?<br><br>3. Are there women's support groups in the community served by this facility?	Are community stakeholders involved in addressing disrespect and abuse of women during childbirth?			

		4.If yes, do you have any formal link/communication with these women's support group? If yes, please describe?				
9.The proportion of all health facility leaders who were trained in leadership and management skills.		1.Does the facility have quality improvement programme, which includes i. Quality improvement mentoring/coaching, ii. Monitoring and evaluation?<br><br>2. Do you have minutes of management meetings?	Have you received refresher/in-service training in the past 12 months?			
10. A policy is in place for staff to provide	Is there a complaint/suggestion box or	1. Routine review				

feedback to the facility management on quality improvement and their performance.	other means of receiving anonymous feedback from clientele?	monitoring of care quality<br><br>2. How does the facility monitor its performance?<br><br>3. Do you have minutes of management meetings?<br><br>4. what does the facility do with data collected on patient care?<br><br>6. Have the data been used to make programme and management decisions?				
Output/process measures	F1.PSFR	F2.MI	F3.SIV	F4.OPCI	F5.CMRR	F6.WEICPC

1.Health facility leaders communicated the performance of the facility through established mechanisms for monitoring (e.g., a dashboard of key metrics) to all relevant staff.	1. Does this facility routinely conduct audit for maternal deaths?<br><br>2. Does this facility routinely conduct audit for early neonatal deaths?<br><br>3. Does this facility routinely conduct audit for stillbirths?	1.Does the facility have quality improvement programme that has Quality improvement teams?<br><br>2. quality improvement action plans?<br><br>3. Quality improvement mentoring/coaching?<br><br>4. Capturing and use of data?<br>5. Monitoring and evaluation?<br><br>6. Do you have minutes of management meetings?				

2.The proportion of monthly meetings on the quality of care that were actually held in the preceding 12 months.	1. Does this facility routinely conduct audit for maternal deaths?<br><br>2. Does this facility routinely conduct audit for early neonatal deaths?<br><br>3. Does this facility routinely conduct audit for stillbirths?	1.Does the facility have quality improvement programme, which includes Quality improvement action plans? Quality improvement mentoring/coaching, and Monitoring and evaluation?<br>2.Do you have minutes of management meetings?				
Outcome measures	F1.PSFR	F2.MI	F3.SIV	F4.OPCI	Fomr5	F6.WEICPC
1.The proportion of all women who gave birth at the health facility		1. Do facility training sessions cover patient's rights?	1. Is there a policy and procedure for addressing patient			In general, how satisfied are you with the care you received during your

who were satisfied with the care and support from facility staff.		2. Facility specific management policy on respectful and dignified care for all	concerns in this facility?<br><br>2. Does your facility have clear policies on rights of the patient?<br><br>3. Have you received training on how to treat childbearing women with compassion and dignity (customer care)?			delivery at this facility?<br><br>2. are you satisfied with the level of attention and care given to your newborn baby after delivery?
2.Evidence for improved performance of the system (according to the facility dashboard).	1. Does this facility routinely conduct audit for maternal deaths?<br><br>2. Does this facility	1.How does the facility monitor its performance?<br>2.How does the facility monitor staff performance?				

	routinely conduct audit for early neonatal deaths?<br><br>3. Does this facility routinely conduct audit for stillbirths?	3.Is there data on number of deliveries in this facility in a year?<br>4.How frequently are charts produced?<br>5.Is there data on numbers of sick newborns in this facility in a year?<br>6.How recent is the recent chart; record month and year?<br>7.Have the facility used the data to make programme and management decisions?<br>8.If yes, describe the most recent				

		example for maternal and newborn care.				
Standard 8: The health facility has an appropriate physical environment, with adequate water, sanitation and energy supplies, medicines, supplies and equipment for routine maternal and newborn care and management of complications.						
Quality statement 8.1						
Water, energy, sanitation, hand hygiene and waste disposal facilities are functioning, reliable, safe and sufficient to meet the needs of staff, women and their families.						
Input measures	F1.PSFR	F2.MI	F3.SIV	F4.OPCI	F5.CMRR	F6.WEICPC
1.The health facility has a functioning source of safe water located on the premises that is adequate to meet all demands for drinking, personal hygiene, medical interventions, cleaning, laundry and cooking for use by staff, women,	1.What is the main source of water for cleaning in the facility?<br><br>2.What is the man source of drinking water for the facility?<br><br>3.Is there a system for checking the quality of drinking water in this facility?			Are functioning sinks with clean, running water available in all clinical rooms/wards/treatment areas for handwashing (one per ward)?		Did you have soap and water to wash your hands after visiting the toilet?

newborns and their families.						
2.The health facility has leak-proof, covered, labelled waste bins and impermeable sharps containers available in every treatment area, to allow segregation of waste into four categories: sharps, non-sharps infectious waste, general non-infectious waste (e.g., food, packaging) and anatomical waste (e.g., placenta).	1.Are there waste bins in the maternity and labour ward?<br><br>2.Are there waste bins in the maternity and labour ward colour coded and labelled?<br><br>3.Are puncture proof containers provided in each clinical area?<br><br>4.Is the sharp container in the labour ward less than three-quarter full?(check)	Facility specific management policies on infectious control and notification system	Is there an Infection Prevention and Control (IPC) policy available in your facility?	1.Are puncture proof containers located in all clinical areas?<br><br>2.Are sharp containers no more than ¾ full?		

	5.Is there a concrete-lined sharp pit or incinerator for sharps disposal?					
3.The health facility has at least one functioning hand hygiene station per 10 beds, with soap and water or alcohol-based hand rubs, in all wards.	1. Is there handwashing facility on the maternity toilet?<br><br>2. What is used for handwashing on the maternity toilet?<br><br>3. How do users of the maternity toilet clean their hands after handwashing?	Facility specific management policies on infectious control and notification system	1.Is there an Infection Prevention and Control (IPC) policy available in your facility?<br><br>2. How often do you wash your hands before and after examining a patient?<br><br>3. Are you able to mention the "five moments" of hand hygiene?	1. Are functioning sinks with clean running water available in all clinical rooms/wards/treatment areas for hand washing (one per ward/room)<br><br>2.Are sinks equipped with bar/liquid soap in clinical areas?		Did you have soap and water to wash your hands after visiting the toilet?
4.The health facility has energy infrastructure	1.What is the main source of power for the facility?					

(e.g., solar, generator, grid) that can meet all the electricity demands of the facility and associated infrastructure at all times, with a backup power source.	2. If the source of power is national grind, is there a backup power supply?					
5.The health facility has written, up-to-date protocols and awareness-raising materials (posters) on cleaning and disinfection, hand hygiene, operating and maintaining water, sanitation and hygiene	1. Is there a protocol for infection control?<br><br>2. Are there waster binds in the maternity and labour ward colour-coded and labelled?<br><br>3. is there a pit disposal of placentae?	Facility specific management policies on infectious control and notification system	1.Is there an Infection Prevention and Control (IPC) policy available in your facility?<br><br>2. Do you use Chlorine to decontaminate used equipment?<br><br>3. What is the stock strength	Did staff wash their hands before and after examining patients?<br><br>2. was delivery unit cleaned after the delivery?<br><br>3. are healthcare workers compliant with the hand		

facilities and safe waste management; these are posted in the areas in which the activities are conducted.			of chlorine in your facility?<br><br>4. What is the stock strength of chlorine in your facility?<br><br>5. How often is this solution changed per day?	hygiene "five moments"		
6.The health facility has sanitation facilities on premises that are usable, appropriately illuminated at night, accessible to people with limited mobility and separated by gender for staff and patients; they	1.Is there toilet facility for clientele on the maternity ward?<br><br>2.Is there a separate toilet for clientele on the maternity ward?<br>1.Is the maternity toilet clean?(inspect).	Facility specific management policies on infectious control and notification system	1.Is there an Infection Prevention and Control (IPC) policy available in your facility?	1.Are functioning sinks with clean running water available in all clinical rooms/wards/treatment areas for handwashing (one per ward)<br><br>2.Are sinks equipped with bar/liquid soap		1. Is there a toilet for clients in the maternity or children's ward?<br><br>2. What is the state of the cleanliness on the toilet?<br><br>3. Did you have soap and water to wash your hands after

include at least one toilet that meets the needs for menstrual hygiene management, with hand-washing stations and soap and water (at least 1 latrine per 20 users for inpatient settings).				in clinical areas?		visiting the toilet?
7.The health facility has sufficient trained, competent staff on site when needed, with clear descriptions of their responsibilities for cleaning, operating and	1. How many of the (neonatologist, obstetrician/Gynaecologist, Paediatricians, Medical Officers, Midwives, Enrolled nurse midwives, other nurses, others, specify)	1.What are the minimum qualifications to work in the maternity wards?<br><br>2. What are the minimum qualifications to work in the children's wards?	1. What is your highest level of education?<br><br>2.What is your designation(professional qualification)<br><br>3. are you able to mention the "five moments"	How many staff were on duty?	Who performed the surgery?	

maintaining water, sanitation, hygiene and health care waste facilities.	professionals do you have in this facility?<br><br>2. How many are in the maternity?	3. are there additional refresher training for the staff?	of hand hygiene?<br><br>4. is there an IPC focal person?			
8.The health facility has sufficient funds for rehabilitation, improvement and continuous operation and maintenance of water, sanitation, hygiene and health care waste services.		1.How are drugs and supplies purchased for use in the maternal and newborn care wards?F2.MI who makes the request?<br>2. Is there a system of justification for the request?<br>3. Who does the justification and how? Rank<br>4. If justified, how are procurement made? centrally				

		or by department?<br>5. How are equipment maintained in the facility?<br>6. What happens if an equipment is damaged?				
9.The heath facility has a fuel management plan and a local buffer stock, supported by an adequate budget for all the fuel needs for vehicles, cooking and heating, as relevant and as required, at all times.		1.How are drugs and supplies purchased for use in the maternal and newborn care wards?<br>2. if justified, how are procurement made? centrally or by department?<br>3. How are equipment maintained in the facility?				

10.The health facility has a preventive risk plan for managing and improving water, sanitation and hygiene services, including for infection prevention and control.	1.Is there a system for checking the quality of drinking water in this facility?<br><br>2.Is there a protocol for infection control?	1.Does the facility have a specific policy on infectious control and notification system?	Is there an Infection Prevention and Control (IPC) policy available in your facility?	1.Are puncture proof sharps containers located in all clinical areas?<br><br>2. Is there a concrete line sharp pit or incinerator for sharps disposal?<br><br>3. Are functioning sinks with clean, running water available in all clinical rooms/wards/treatment areas for handwashing (one per ward)		
11.The heath facility has an energy management plan supported by an adequate	1. Level of the facility (hospital, health centre, tertiary facility)	1.How are drugs and supplies purchased for use in the maternal and	1.Does this facility offer 24hr services?<br><br>2. Are delivery services			

budget, maintained by appropriately trained staff and regulated by a competent authority.	2. Designation of the main respondent.<br><br>3. If the source of power is mainly national grind, is there a backup power supply?<br><br>4. Does the facility admit patients over night?<br><br>5. how many of the professionals do you have in this facility? How many are in the maternity?	newborn care wards?<br><br>2. Who does the justification and how? Rank<br><br>3.How are equipment maintained in the facility?<br><br>4. What happens if an equipment is damaged?	available for 24hrs in a day?<br><br>3. Are patients admitted to this facility overnight?			
Outcome measures	F1.PSFR	F2.MI	F3.SIV	F4.OPCI	F5.CMRR	F6.WEICPC
1.The proportion of						1.Overall, how satisfied were

women and their families who attended the health facility who were satisfied with the water, sanitation and energy services and would recommend the health facility to friends and family.						you with the hygiene standards at the health facility?<br><br>2.Would you recommend this health facility to a relative or friend for them to deliver?
2.The proportion of all health care staff at the health facility who were satisfied with the water, sanitation and energy services and considered that these services contribute positively to						1.Overall, how satisfied were you with the hygiene standards at the health facility?

providing high-quality care.						
3.The proportion of women and their families who attended the health facility who were satisfied with the power and lighting source and would recommend the health facility to friends and family.						1.In general, how satisfied are you with the care you received during your delivery at this facility?<br><br>2.In general, what did you like?<br><br>3.What did you not like?
Quality statement 8.2						
Areas for labour, childbirth and postnatal care are designed, organized and maintained so that every woman and newborn can be cared for according to their needs in private, to facilitate the continuity of care.						
Input measures	F1.PSFR	F2.MI	F3.SIV	F4.OPCI	F5.CMRR	F6.WEICPC
1.The health facility has a dedicated area in the labour and childbirth	1. Does the facility have functioning Stethoscope, Thermometer –		Does this facility provide resuscitation by cleaning of airway,	1.list the items in the resuscitation area:		

area for resuscitation of newborns, which is adequately equipped with a table or resuscitative, radiant warmer, light and appropriate resuscitation equipment and supplies.	digital/mercury column, sucker machine, filled oxygen cylinder, oxygen cylinder carrier, bag and mask (for babies), spot light, radiant warmers, IV fluid, IV stand?<br><br>2. Does this facility ‘s ambulance have stretcher or bed, oxygen supply, nasal prongs, bag and mask, Resuscitation tray, means of hanging IV fluids, drugs or blood, Cannulae, fetal heart monitor, ventilator, Tocolytics?		stimulation, Bag and mask ventilation and were they done in the past 6 months?<br><br>Does this facility provide comprehensive services such as newborn intensive care unit, advanced resuscitation, oxygen (blended), intubation, nasal CPAP, surfactant, third generation cephalosporin, IV fluid administration, ACS administration and were the	clean towels- at least 2, ambu bag, neonatal sized mask, radiant warmer, build syringe for aspiration of fluids, oxygen cylinder with oxygen.<br><br>2. newborn resuscitation equipment checked for functioning status.		

			provided in the past 6 months?			
2.The health facility has a labour ward and an adequate number of birthing rooms or areas for the estimated number of births in the service area.	Does the facility have a separate maternity ward?<br><br>What is the total bed capacity?	Are maternity and children's ward run separately?	Are delivery services available for 24hrs in a day?	1.How is the layout of the labour ward.<br><br>2. How many beds are in the labour wards?<br><br>3. How is the layout of the delivery ward?	1.Time of delivery<br><br>2. Type of delivery<br><br>3. Delivery outcome for newborn<br><br>4. Was the woman examined any time after delivery before discharge?<br><br>5. Was/were baby(ies) examined any time after birth before discharge?	Did they tell you what they found before sending you to labour ward?
3.The health facility has clean,	1.Does the facility have functioning			1.Were women's privacy ensured/maintai		1. What was the state of the

appropriately illuminated, well-ventilated labour, childbirth and neonatal areas and surroundings that allow for privacy and are adequately equipped, regularly cleaned and maintained.	screen/curtains for privacy?<br><br>2. Is the facility clean(free of litter, walls and doors unstained, furniture and facilities in good order in all areas?			ned during interaction with the health worker?<br><br>2. Was the woman's privacy maintained during the examinations?		cleanliness of the toilets?<br><br>2. Overall, how satisfied were you with the hygiene standards at the health facility?<br><br>3. were you worried that other people were seeing you when you were being examined by the staff/healthcare worker?<br><br>4. were you worried that other people were hearing you when you were talking to staff/healthcare.

4.The health facility practises and enables rooming-in for all women to allow mothers and babies to remain together 24 h a day.	1.Does this facility have a separate maternity ward?<br><br>2. Does this facility provide KMC ward/beds?<br><br>3. Graduated cup to measure expressed breast milk?<br><br>4. Small cup for feeding breastmilk?	1.Does the facility have specific management policies on i) Baby friendly, ii) Rooming in, and iii) partner/family involvement in maternity and newborn care?	1.Does this facility provide skin to skin contact as a newborn signal function and was it done in the past 6 months?<br><br>2. Recorded evidence of KMC (prolonged STSC, EBF/feeding support, growth monitoring (weight) provided in this facility and was it done in the past 6 months?	1. Baby delivered unto mother's abdomen<br><br>2. Baby placed skin to skin contact with the mother immediately after birth<br><br>3. When was the baby taken out of skin to skin contact with the mother?		Was the newborn put skin to skin with the mother immediately after birth?
5.The health facility has a labour and childbirth area or room with a functional,	1. Is there a toilet facility for clientele on the maternity ward?					1.Is there a toilet for clients in the maternity ward?

clean and accessible bathroom or shower room and toilet for use only by women in labour.	2. Is there a separate toilet for clientele on the maternity ward?<br><br>3. Is the maternity toilet clean (inspect).					2. What was the state of the cleanliness on the toilet?
6. A facility offering surgical services has adequately equipped operating theatre located close to and easily accessible from the labour and childbirth areas.	Does the facility operate a theatre?		Is Caesarean Section delivery provided in this facility as a signal EmOC function and was it done in the past 6 months?	Plan for delivery communicated to the mother (SVD/assisted vaginal delivery/C/S)	1.Was it an emergency or elective C/S?<br>2.How long (hours and minutes) after the decision for C/S was the actual surgery performed?	Type of delivery (spontaneous vaginal delivery/assisted with vacuum instruments/Caesarean Section)<br><br>If Caesarean Section, was it already planned during pregnancy?
7.The facility has a dedicated recovery room or area for care of women with complications.	Does this facility have a separate maternity ward?		Does this facility also admit pregnant women who come here and are considered	1.Was the woman referred from another facility to this one?	What was done to a woman with an obstetric complication?	

			not fit to go home?	2. Was there any complications in the labour and the delivery?		
8.The health facility has a dedicated ward for admitting sick and unstable small babies.	Does this facility have a separate paediatric ward?	Are maternity and children's wards separately run?	Does this facility operate a static child welfare clinic?		What was done for the baby(ies) with complications?	
Output/process measures	F1.PSFR	F2.MI	F3.SIV	F4.OPCI	F5.CMRR	F6.WEICPC
1.The proportion of all pregnant women who attended the health facility who reported that it has a clean physical environment conductive for childbirth.	Is the facility clean (free of litter, walls and doors unstained, furniture and facilities in good order in all areas)?					1.Overall, how satisfied were you with the hygiene standards at the health facility?<br>2.In general, what did you like?<br>3.What did you not like?

Outcome measures	F1.PSFR	F2.MI	F3.SIV	F4.OPCI	F5.CMRR	F6.WEICPC
1.The proportion of all women who gave birth in the health facility who were satisfied with the environment of the labour and childbirth area, including the cleanliness, proximity to a toilet, general lightning, level of crowding and privacy.	Is the facility clean (free of litter, walls and doors unstained, furniture and facilities in good order in all areas)?					1.Overall, how satisfied were you with the hygiene standards at the health facility?<br>2.In general, what did you like?<br>What did you not like?<br>3.What is the state of the cleanliness on the toilet?<br>4.Were you worried that other people were seeing you when you were being examined by the staff/health care workers.
Quality statement 8.3						

Adequate stocks of medicines, supplies and equipment are available for routine care and management of complications						
Input measures	F1.PSFR	F2.MI	F3.SIV	F4.OPCI	F5.CMRR	F6.WEICPC
1.The health facility has supplies of antihypertensive agents and magnesium sulphate in sufficient quantities, available at all times, in antenatal, labour, childbirth and postnatal areas for the management of women with pre-eclampsia.	Does the health facility have the (IV antibiotics, Magnesium Sulphate (MgS04), IV Diazepam, Oxytocic (Syntomentrine/ Ergot), Dexamethasone /Betamethasone (parenteral)) essential drugs?		1.Have you received refresher/in-service training in the past 12 months on management of Pre-eclampsia and/ or eclampsia?<br><br>Vignette:<br>2. Giving antihypertensive drug, e.g., hydralazine, labetalol or Nifedine<br><br>3. Give Magnesium sulphate or, if not available, Diazepam.		What was done with a woman who suffered from Eclampsia as a complication during pregnancy?	Were you given IV fluid/medicine to take/injection(IM), pain relief during labour?

2.The health facility has uterotonic drugs and supplies for intravenous infusion (syringes, needles, infusion sets, intravenous fluid solutions and blood) available in sufficient quantities at all times in the childbirth and postnatal care areas for the management of women with postpartum haemorrhage.	Does the health facility have the (IV antibiotics, Magnesium Sulphate (MgS04), IV Diazepam, Oxytocic (Syntomentrine/ Ergot), Dexamethasone /Betamethasone (parenteral), Hydralazine/SL Nifedipine, Ampicillin/Penicillin for IV, Gentamicin for IV, Cephalosporins) essential drugs?		1.Does this facility provide blood transfusion services as signal EmOC functions and was it provided in the last 6 months?<br><br>2.Does this facility provide injectable oxytocic (synto/ergot) drug administration as signal EmOC functions and was it provided in the last 6 months?		Was the woman given parenteral oxytocin?	Were you given IV fluid/medicine to take/injection(IM), pain relief during labour?
3.The health facility has supplies of antenatal corticosteroids	Does the health facility have the (IV antibiotics, Magnesium Sulphate		1.Is injectable antibiotics administration provided in this facility as signal		Did the woman receive any antibiotic?	Were you given IV fluid/medicine to take/injection(I

(dexamethasone or betamethasone) , antibiotics and magnesium sulphate available in sufficient quantities at all times to manage preterm births.	(MgS04), Dexamethasone /Betamethasone (parenteral)) essential drugs?		EmOC function and was it done/provided in the past 6 months?<br><br>Vignette:<br>2. Give magnesium sulphate or, if not available, diazepam		What antibiotics was given?	M), pain relief during labour?
4.The health facility has functioning essential equipment and supplies for the detection of complications (e.g., thermometers, sphygmomano meters, foetal stethoscopes, urine dipsticks, pulse oximeter)	Does the facility has functioning glucometer, monitor (pulse oximeter), Ultrasound scan machine, fetoscope/sonic aid, BP apparatus/Sphy gmomanometer, stethoscope and uristix/dipstick?	1.Laboratory equipment?<br>2.Sphygmoman ometers?<br>3.Ultrasound machines<br>4.X-ray machines Glucometers	1.Vignettes#1: measure woman's blood pressure/check her urine for protein/check her reflexes/check fetal heart rate<br><br>2.Vignette#2: Giving antihypertensiv e drug, e.g., hydralazine,	1.Listened to fetal heart?<br>2. Blood pressure taken?<br>3. Urine test done?	1.How many times was the woman's temperature checked and recorded between first exam and delivery?<br><br>2. How many times was blood pressure the checked and recorded	1.Check your BP<br>2. Take your urine and checked<br>3. Examined your abdomen<br>4. Listened to your baby's fetal heart rate?

in sufficient quantities at all times in the labour and childbirth areas of the maternity unit.			labetalol or nifedine/ Give magnesium sulphate or, if not available, diazepam/ Give diuretics		between first exam and delivery?<br><br>3. How many times was the mother's pulse checked and recorded between first exam and delivery?<br><br>4. Was the fetal heart rate recorded?	
5.The health facility has supplies of first- and second-line injectable antibiotics and other essential medicines available at all times for the	Does the health facility have essential drugs of IV antibiotics, Magnesium Sulphate (MgS04), IV Diazepam, Oxytocic		1.Is injectable antibiotics administration provided in this facility as signal EmOC function and was it done/provided in the past 6 months?	1.Did the health worker set an IV line on woman?<br><br>2. Was the labour augmented at any time?	1.Did the woman receive any antibiotic?<br>2. Was the woman given parenteral oxytocin?<br>3. Women received misoprostol?	1.Were you given any of the IV fluid?<br><br>2.medicine to take<br><br>3. injection(IM)<br><br>4. pain relief?

management of women and newborns with, or at risk for, infections during labour, childbirth and the early postnatal period.	(Syntomentrine/ Ergot), Dexamethasone /Betamethasone (parenteral), Hydralazine/SL Nifedipine, Ampicillin/Penicillin for IV, Gentamicin for IV, Cephalosporins) essential drugs.		Vignettes.<br>2.Giving antihypertensive drug, e.g., hydralazine, labetalol or nifedine<br><br>3.Give magnesium sulphate or, if not available, diazepam<br><br>4.Give diuretics		Woman received IV fluid?	
6.The health facility has essential laboratory supplies and tests (blood glucose, haemoglobin or packed cell volume, blood group and cross-matching,	Does the facility provide laboratory, uristix /dipstick, glucometer services?	1.Laboratory equipment<br><br>2.Glucometers	Does this facility offer laboratory services?	Was her blood sample taken?		

bilirubin, urine protein, full blood count, blood culture, electrolytes, renal and liver function tests, syphilis, HIV and malaria rapid diagnostic tests) to support the management of women and newborns.						
7.The health facility has essential supplies and functioning equipment (including childbirth beds, vacuum, forceps, incubators, weighing machine, sterile	1.What is the total bed capacity?<br><br>2.Does the facility has functioning BP apparatus/Sphygmomanometers, sterile gloves, weighing machine, incubator,	Is there a form of external calibration of equipment used in this facility such as standards board's/authority: weighing scales, laboratory equipment, Sphygmomano	Was Vitamin K, Weight as Newborn signal function provided in this facility and were the provided/done in the past 6 months?	1.Baby weighed?<br><br>2.Did the staff wear gloves when handling medical waste?<br><br>3.Puts on sterile gloves?	1.Is/are birth weights recorded for all babies?<br><br>2.Birth weight of baby1/baby2/baby3	Did the health worker Check your BP, take your urine and checked, listened to your baby's heart beats before sending you to labour ward?

gloves) available in sufficient quantities at all times in the labour and childbirth areas.	delivery forceps, and vacuum aspirator?	meters, ultrasound machines, X-ray machines, glucometer?				
8.The health facility has supplies and functioning equipment for the emergency care and resuscitation of women (well-stocked resuscitation trolley, suction device, pulse oximeter, airways, laryngoscope, endotracheal tubes, adult bag valve masks, infusion sets, intravenous fluids) available	Does the facility have functioning sucker machine, pulse oximeter, IV fluid, IV stand, IV canula, and endotracheal tube, filled oxygen cylinder, oxygen cylinder carrier?		Is resuscitation provided in this facility by cleaning of airway, stimulation, bag and mask ventilation and were the done in the past 6 months?	1.Check delivery trolley and instruments for their functioning status?<br><br>2. Newborn resuscitation equipment checked for functioning status	1.Woman received IV fluid?<br>2.If woman received IV fluid why was it given?	Were you given any of the IV fluid?/Injection (IM)

in sufficient quantities all times in areas designated for labour, childbirth and postnatal care.						
9.The health facility has a safe, uninterrupted oxygen source and delivery supplies (nasal prongs, catheters and masks), including nasal continuous positive airway pressure, available at all times in labour, childbirth and neonatal areas and the operating	1.What is the main source of power for the facility?<br><br>2.Does the facility have bag and mask (for babies), nasal oxygen prongs.<br><br>3. filled oxygen cylinder<br><br>4. oxygen cylinder carrier		Are delivery services available for 24hirs in a day?<br><br>Is/are Newborn Intensive Care Unit, advanced resuscitation, oxygen (blended), intubation, Nasal CPAP, Acute Chest Syndrome (ACS administration provided in this facility as comprehensive services and were they			

theatre (when available).			done/provided in the past 6 months?			
10.The health facility has supplies and functioning equipment for emergency care and resuscitation of newborns (resuscitation table, well-stocked neonatal resuscitation trolley, warmer, suction device, pulse oximeter, laryngoscope) available all times in areas designated for labour, childbirth and neonatal care.	Does the facility have functioning sucker machine, pulse oximeter, IV fluid, IV stand, IV canula, radiant warmer and endotracheal tube?		Have you received refresher/in-service training in the past 12 months on Asphyxia/Resus citation?	List the items in the resuscitation area(Clean towels-at least 2/ambu bag/neonatal sized mask/radiant warmer/Buld syringe for aspiration of fluids/oxygen cylinder with oxygen?)		

11.The health facility has an on-site pharmacy and a medicine and supplies stock management system managed by a trained pharmacist or dispenser.	1.Does the facility has the pharmacy/dispensary services?<br><br>2.How many of the following professionals do you have in this facility?	1.How are drugs and supplies purchased for use in the maternal and newborn care wards?<br><br>2. Who makes the requests?<br><br>3. Is there a system of justification for the request?<br><br>4. Who does the justification and how? rank				
12.The health facility has a dedicated budget for essential medicines, equipment (and its maintenance)	Level of the facility (hospital, health centre, tertiary facility)<br><br>Does the facility admit patients over night	1.How are drugs and supplies purchased for use in the maternal and newborn care wards?				

and medical supplies for maternal and newborn care.	Facility has essential drugs (IV antibiotics, Magnesium Sulphate (MgS04), IV Diazepam, Oxytocic (Syntometrine/Ergot), Dexamethasone /Betamethasone (parenteral), Hydralazine/SL Nifedipine, Ampicillin/Penicillin for IV, Gentamicin for IV, Cephalosporins)	2.Who makes the requests for purchases?<br>3.Is there a system of justification for the request?<br>4.Who (rank) does the justification and how?<br>5.If justified, how are the procurement made, centrally?<br>6.How are equipment maintained in the facility?<br>7.What happens when the equipment is damaged?				
13.The health facility has a functioning diagnostic ultrasound	Does the facility provide Ultrasound?	1.Is there a form of external calibration of equipment used in this facility				

machine and trained health staff who can conduct a basic obstetric ultrasound examination to determine the number of foetuses present, gestational age, prenatal diagnosis of foetal anomalies or early diagnosis of placental insufficiency.		such as standards board/authority: ultrasound machines?				
Output/process measures	F1.PSFR	F2.MI	F3.SIV	F4.OPCI	F5.CMRR	F6.WEICPC
1. Availability of essential life-saving medicines (oxytocin, magnesium	1.Does the facility have essential drugs such as Magnesium Sulphate(MgS04		1.Does the facility provide injectable oxytocin (synto/Ergot) drugs	1. mmunized for HepB0,BCG,bOPV.<br><br>2. Was the woman	What antibiotics was given (Ampicillin/Penicillin, Gentamicin)?	Were you given any of IV fluid/medicine to take/injection(I

sulphate, dexamethasone, vitamin K, injectable and oral amoxicillin, benzyl penicillin, gentamicin, ceftriaxone, metronidazole, antimalarial drugs, antiretroviral drugs and vaccines against tuberculosis, hepatitis B, poliomyelitis) in the past three months.	), Oxytocic (Syntometrine/Ergot), Dexamethasone /Betamethasone (parenteral), Ampicillin/Penicillin for IV, Gentamicin for IV, and IV antibiotics.		administration as part of EmOC signal functions and was it provided in the last 6 months?<br><br>2. Does the facility provide Vitamin K as part of Newborn signal functions and was it provided in the last 6 months?<br><br>3. Does the facility manage suspected sepsis with injectable antibiotics (Ampicillin/Penicillin and Gentamicin)?	counselled on immunization.<br><br>3. Does the list of contents of the delivery tray include Oxytocin.<br><br>4. Oxytocin administered.		M) during labour?
2.The proportion of all women who had severe pre-	Essential drug availability: Magnesium		Give Magnesium Sulphate or if		What was done for the woman with a pregnancy	

eclampsia or eclampsia in the health facility who did not receive the full dose of magnesium sulphate because of a stock-out.	Sulphate(MgS04)?		not available Diazepam.		complication (Pre-eclampsia, eclampsia, gestational diabetes, severe anaemia, Antepartum haemorrhage, urinary tract infection…).	
3.The proportion of all women who gave birth in the health facility who purchased gloves and other necessary items.	Does the facility have sterile gloves?	Is the woman expected to buy supplies for her delivery in this facility?	1.Is a woman expected to pay a fee for a normal delivery?<br><br>2. Is a woman expected to buy supplies for a normal delivery?<br><br>3. If a woman is expected to buy supplies, can you mention some of these supplies?	1.Are women supposed to pay for maternity services?<br><br>2.Did the woman or her family pay any monies for the services since entering the facility till discharge?<br><br>3.Did the patient complaint about		1.Did you have to pay anything (cash or kind) for the service provided during labour or delivery?<br>2.Were you asked to purchase some items for yourself or for the baby?

				any monies being charged?		
Outcome measures	F1.PSFR	F2.MI	F3.SIV	F4.OPCI	F5.CMRR	F6.WEICPC
1.The proportion of all nulliparous women with a singleton cephalic foetus at ⩾ 37 weeks of gestation who underwent caesarean section during spontaneous labour (Robson group 1).	Does the facility have a theatre?		Was Caesarean section delivery provided as a signal EmOC functions in this facility and was it done in the past 6 months?	Plan for delivery communicated to mother (SVD/assisted vaginal delivery/C/S)	1.Gravida of woman.<br>2.Number of completed weeks of gestation at delivery.<br>3.Type of delivery.<br>4.Parity of the woman.<br>5.Birth weight baby1/baby2/baby3.	1.Number of all previous pregnancies (including this one)<br><br>2. Type of delivery (spontaneous vaginal delivery/assisted with vacuum instruments/Caesarean Section)
2.The proportion of unmet need for caesarean section as a result of lack of supplies or staff trained to conduct	1.Does the facility have a theatre?<br><br>2.How many of the Neonatologist, Obstetrician/Gynaecologist,	1. What are the minimum qualifications to work in the maternity wards?<br><br>2. What is the minimum	Is Caesarean Section as a signal EmOC function provided in this facility and was it performed in the last 6 months?	Plan for delivery communicated to mother(SVD/assisted vaginal delivery/CS)?	Did the surgeon specify fluid management of the woman after the C/S?	Type of delivery

caesarean section.	Paediatricians, Medical Officers, Midwives, Enrolled nurse midwives, other nurses, other, specify do you have in this facility?<br><br>3.How many are in maternity?	qualifications to work in the children's ward?<br><br>3. What additional training is provided to workers posted in maternity and children's wards?				

Appendix B.

Output/process measures not being captured by any of the tools

Standard	Descriptions
1.9	4.The proportion of all babies born in the health facility who received early bathing and removal of the vernix within 6 h of birth.
	5.The proportion of all women who gave birth in the health facility who received routine pubic or perineal shaving before a vaginal birth.
	7. The proportion of all women who gave birth in the health facility who received routine enemas at any time before vaginal birth.
4.2	2. The proportion of all women who gave birth in the health facility who reported that health care staff introduced themselves and showed good knowledge of the women's history and the care that had been given to date.
6.1	2. The proportion of all companions who were satisfied with the orientation given on their role during labour and childbirth.
6.2	3. The proportion of all women who gave birth in the health facility who reported having sufficient food and drink during labour.
	4.The proportion of all women who gave birth in the health facility who were ambulatory during the first stage of labour.

Outcome quality measures not being assessed by any of the utilized tools

Standard	Descriptions
4.2	2. The proportion of health care staff, by cadre, and social professionals who were satisfied with the communication during clinical hand over among members of the health care team in the health facility.
7.2	3.The proportion of all staff at the health facility who reported being `highly satisfied' with their job.
