# Supplementary material for: Monitoring healthcare improvement for mothers and newborns: A quantitative review of WHO/UNICEF/UNFPA standards using Every Mother Every Newborn assessment tools
Source: Front Pediatr. 2022 Sep 12;10:959482. doi: 10.3389/fped.2022.959482 (PMC9510702; doi:10.3389/fped.2022.959482)
Supplement: Supplementary file 2 [file Data_Sheet_4.PDF]

Figure S2. Every Mother Every Newborn (EMEN) Standards.

EMEN Standards
**Clinical care**
Standard1. Evidence-based safe care is provided during labour and childbirth
Standard2. Evidence based safe postnatal care is provided to all mothers and newborns
**Respect and dignified**
Standard3. Human rights are observed, and the experience of care is dignified and respectful for every woman and newborn
**Governance**
Standard4. A governance system is in place to support the provision of quality maternal and newborn care
**Essential physical resources**
Standard5. The physical environment of the health facility is safe for providing maternal and newborn care
Standard6. Essential medications, supplies, functional equipment and diagnostic services are consistently available for maternal and newborn care
**Competent and motivated human resources**
Standard7. Qualified and competent staff are available in adequate numbers to provide safe, consistent and quality maternal and newborn care.
**Actional information**
Standard8. Health information systems are in place to manage patient clinical records and service data
**Functional referral system**
Standard8. Services are available to ensure a continuity of care for all pregnancy women, mothers and newborns.
