# Supplementary material for: Monitoring healthcare improvement for mothers and newborns: A quantitative review of WHO/UNICEF/UNFPA standards using Every Mother Every Newborn assessment tools
Source: Front Pediatr. 2022 Sep 12;10:959482. doi: 10.3389/fped.2022.959482 (PMC9510702; doi:10.3389/fped.2022.959482)
Supplement: Supplementary file 3 [file Data_Sheet_5.PDF]

Supplementary Material

Table S2. : Comparative WHO/UNICEF/UNFPA STANDARDS for improving quality of maternal Newborn Care in health facility

**Standards of care and quality statements**	
**Standard 1: Every woman and newborn receives routine, evidence-based care and management of complications during labour, childbirth and the early postnatal period, according to WHO guidelines.** *Quality statements*	**EQUIVALENT EMEN Standard**
1.1a: Women are assessed routinely on admission and during labour and childbirth and are given timely, appropriate care.	1. Evidence-based safe care is provided during labour and childbirth.
1.1b: Newborns receive routine care immediately after birth.	2. Evidence-based safe postnatal care is provided for all mothers and the newborns.
1.1c: Mothers and newborns receive routine postnatal care.	2. Evidence-based safe postnatal care is provided for all mothers and the newborns.
1.2: Women with pre-eclampsia or eclampsia promptly receive appropriate interventions, according to WHO guidelines.	1. Evidence-based safe care is provided during labour and childbirth.
1.3: Women with postpartum haemorrhage promptly receive appropriate interventions, according to WHO guidelines.	1. Evidence-based safe care is provided during labour and childbirth.
1.4: Women with delay in labour or whose labour is obstructed receive appropriate interventions, according to WHO guidelines.	1. Evidence-based safe care is provided during labour and childbirth.
1.5: Newborns who are not breathing spontaneously receive appropriate stimulation and resuscitation with a bag-and-mask within 1 min of birth, according to WHO guidelines.	2. Evidence-based safe postnatal care is provided for all mothers and the newborns.
1.6a: Women in preterm labour receive appropriate interventions for both themselves and their babies, according to WHO guidelines.	1. Evidence-based safe care is provided during labour and childbirth.

1.6b: Preterm and small babies receive appropriate care, according to WHO guidelines.	2. Evidence-based safe postnatal care is provided for all mothers and newborns.
1.7a: Women with or at risk for infection during labour, childbirth or the early postnatal period promptly receive appropriate interventions, according to WHO guidelines.<br><br>1.7b: Newborns with suspected infection or risk factors for infection are promptly given antibiotic treatment, according to WHO guidelines.	1. Evidence-based safe care is provided during labour and childbirth.<br>2. Evidence-based safe postnatal care is provided for all mothers and newborns.<br><br>2. Evidence-based safe postnatal care is provided for all mothers and newborns.
1.8: All women and newborns receive care according to standard precautions for preventing hospital-acquired infections.<br><br>1.9: No woman or newborn is subjected to unnecessary or harmful practices during labour, childbirth and the early postnatal period.	5. The physical environment of the health facility is safe for providing maternal and newborn care.<br><br>3. Human rights area observed and the experience of care is dignified and respectful for every woman and newborn.
**Standard 2: The health information system enables use of data to ensure early, appropriate action to improve the care of every woman and newborn.**	**EQUIVALENT EMEN Standard**
Quality statements	
2.1: Every woman and newborn has a complete, accurate, standardized medical record during labour, childbirth and the early postnatal period.<br>2.2: Every health facility has a mechanism for data collection, analysis and feedback as part of its activities for monitoring and improving performance around the time of childbirth.	8. Health information systems are in place to manage patient clinical records and service data
**Standard 3: Every woman and newborn with condition(s) that cannot be dealt with effectively with the available resources is appropriately referred.**	**EQUIVALENT EMEN Standard**
Quality statements	
3.1: Every woman and newborn is appropriately assessed on admission, during labour and in the early postnatal period to determine	9. Services are available to ensure continuity of care for all pregnant women, mothers and newborns.

whether referral is required, and the decision to refer is made without delay.<br><br>3.2: For every woman and newborn who requires referral, the referral follows a pre-established plan that can be implemented without delay at any time.<br>3.3: For every woman and newborn referred within or between health facilities, there is appropriate information exchange and feedback to relevant health care staff.	
**Standard 4: Communication with women and their families is effective and responds to their needs and preferences.**	**EQUIVALENT EMEN Standard**
Quality statements	
4.1: All women and their families receive information about the care and have effective interactions with staff.<br>4.2: All women and their families experience coordinated care, with clear, accurate information exchange between relevant health and social care professionals.	3. Human rights are observed and the experience of care is dignified and respectful for every woman and newborn
**Standard 5: Women and newborns receive care with respect and preservation of their dignity.**	**EQUIVALENT EMEN Standard**
Quality statements	
5.1: All women and newborns have privacy around the time of labour and childbirth, and their confidentiality is respected<br>5.2: No woman or newborn is subjected to mistreatment, such as physical, sexual or verbal abuse, discrimination, neglect, detainment, extortion or denial of services.<br><br>5.3: All women have informed choices in the services they receive, and the reasons for interventions or outcomes are clearly explained.	3. Human rights are observed and the experience of care is dignified and respectful for every woman and newborn

**Standard 6: Every woman and her family are provided with emotional support that is sensitive to their needs and strengthens the woman's capability.**	**EQUIVALENT EMEN Standard**
Quality statements	
6.1: Every woman is offered the option to experience labour and childbirth with the companion of her choice.<br>6.2: Every woman receives support to strengthen her capability during childbirth.	3. Human rights are observed and the experience of care is dignified and respectful for every woman and newborn
**Standard 7: For every woman and newborn, competent, motivated staff are consistently available to provide routine care and manage complications.**	**EQUIVALENT EMEN Standard**
Quality statements	
7.1: Every woman and child has access at all times to at least one skilled birth attendant and support staff for routine care and management of complications.<br>7.2: The skilled birth attendants and support staff have appropriate competence and skills mix to meet the requirements of labour, childbirth and the early postnatal period.	7. Qualified and competent staff are available in adequate numbers to provide safe, consistent and quality maternal and newborn care.
7.3: Every health facility has managerial and clinical leadership that is collectively responsible for developing and implementing appropriate policies and fosters an environment that supports facility staff in continuous quality improvement.	4. A governance system is in place to support the provision of quality maternal and newborn care
**Standard 7: For every woman and newborn, competent, motivated staff are consistently available to provide routine care and manage complications.**	**EQUIVALENT EMEN Standard**
Quality statements	
7.1: Every woman and child has access at all times to at least one skilled birth attendant and support staff for routine care and management of complications.	7. Qualified and competent staff are available in adequate numbers to provide safe, consistent and quality maternal and newborn care.

7.2: The skilled birth attendants and support staff have appropriate competence and skills mix to meet the requirements of labour, childbirth and the early postnatal period.	
7.3: Every health facility has managerial and clinical leadership that is collectively responsible for developing and implementing appropriate policies and fosters an environment that supports facility staff in continuous quality improvement.	5. A governance system is in place to support the provision of quality maternal and newborn care
**Standard 8: The health facility has an appropriate physical environment, with adequate water, sanitation and energy supplies, medicines, supplies and equipment for routine maternal and newborn care and management of complications.**	**EQUIVALENT EMEN Standard**
Quality statements	
8.1: Water, energy, sanitation, hand hygiene and waste disposal facilities are functional, reliable, safe and sufficient to meet the needs of staff, women and their families.<br>8.2: Areas for labour, childbirth and postnatal care are designed, organized and maintained so that every woman and newborn can be cared for according to their needs in private, to facilitate the continuity of care.	5. The physical environment of the health facility is safe for providing maternal and newborn care
8.3: An adequate stock of medicines, supplies and equipment is available for routine care and management of complications.	6. Essential drugs, supplies and functional equipment and diagnostic services are consistently available for maternal and newborn care.
