# Supplementary material for: Monitoring healthcare improvement for mothers and newborns: A quantitative review of WHO/UNICEF/UNFPA standards using Every Mother Every Newborn assessment tools
Source: Front Pediatr. 2022 Sep 12;10:959482. doi: 10.3389/fped.2022.959482 (PMC9510702; doi:10.3389/fped.2022.959482)
Supplement: Supplementary file 4 [file Data_Sheet_6.PDF]

Supplementary Material

**Table S3: EMEN Quality of care assessment framework: A composite to assess inputs, processes and outputs together with user-perspectives**					
A. Is the necessary infrastructure availability to support QoC?	B. Is there good governance with enabling policy environment for QoC/	C. Are the requisite person-power and skills available to provide QoC?	D. What are the actual practices around care for mothers and newborns?	E. Evidence that observed practices have been routine or influenced by assessor's presence?	F. What are the client's perspectives on the QoC?
**Infrastructure &inputs**:<br>a. Physical space - Dedicated maternal and newborn wards &KMC and/ or intensive care units, client toilets, etc.<br>b. Services – pharmacy, laboratory, radiology, sterilization & incineration, 24hr delivery, ANC, PNC, PMTCT, KMC, NICU, Mental health, EmONC, etc.<br>c. **Equipment-** sphygmomanometers, stethoscopes, fetoscopes/sonic aids, ultrasound machines, pulse oximeters, incubators, bags &masks, ventilation support, glucometers.<br>d. Drugs – antibiotics, Magnesium sulphate, corticosteroids,	**Documentation and existence** of the following:<br>**a.** Facility policies for QOC**-**<br>i. Baby friendly facility and/or breastfeeding guidelines,<br>ii. Procurement<br>iii. Clinical care guidelines development and adaptation process,<br>iv. Quality assurance,<br>v. Right-based and respectful care,<br>vi. Complaint systems and feedback on care giving,	**Person -power and skills to provide quality care to mothers and newborns** including:<br>a. Number of staff- midwives, obstetricians, Neonatal nurses, general Doctors, nurses, other cadres of health professionals, etc.<br>b. Training –<br>i. Recruitment and placement of skilled staff for MNCH,<br>ii. Staff receiving refresher training in the previous year on care for	**Client -provider interaction**:<br>a. Process of care giving-<br>i. Timeliness of care<br>ii. Planning of the care to pre-empt possible complications.<br>iii. Organization of the clinical environment including cleanliness, etc.<br>iv. Privacy of the care giving<br>v. Respectful communication<br>vi. Adherence to infection control protocols.<br>vii. Safe disposal of clinical waste including sharps.	**Review of facility records again** for<br>a. Process of care giving-<br>i. Timeliness of the care<br>b. Content of care – assess thoroughness of the care provided including<br>i. Adherence to protocols<br>ii. Use of the right equipment<br>iii. Availability and use of the right drugs<br>C. Documentation of care –<br>i. Use of correct documents to	**Interviews with clientele and accompanying persons for their perspectives** on<br>a. Process of care giving-<br>i. Timeliness of the care<br>ii. Privacy of the care giving<br>iii. Respectful communication<br>iv. Thoroughness of care -history, exams, investigations<br>v. Duration of stay at facility, abuses and payment for services - approved or unapproved.<br>vi. Ease of transmission through care giving areas.

oxytocic, antimalarials, iron &folate, antihypertensives and antidiabetics.<br>e. Supplies- Sharps containers, PPE, etc<br>f. Ancillary but essential services- Patient toilets in the maternity wards, water, and soap for handwashing, etc.	vii. Abuse prevention and resolution,<br>viii. Essential drug lists,<br>ix. Payment for services,<br>x. Care for the poor & needy,<br>xi. Working hours and outreach services,<br>xii. Recruitment,<br>xiii. Use of data to inform QoC/facility policy, etc.<br>b. Guidelines and protocols-<br>i. Care for pregnant women, newborns, and children<br>ii. Use of partograph	pregnant women and newborns.<br>c. Knowledge of staff – Do staff have the requisite knowledge to provide interventions for pregnant women and newborns at optimal quality?<br>d. Motivation of staff – are staff motivated to provide quality; What motivation measures?	viii. Ease of transmission through various departments in care giving<br>b. Content of care – assess thoroughness of the care provided including<br>i. Adherence to protocols<br>ii. Use of the right equipment.<br>iii. Availability and use of the right drugs<br>c. Documentation of care – use of correct documents to record care provided e.g., Partographs, etc.?	record care provided<br>ii. Record of the diagnosis<br>iii. Record of communication with the client and how this affected the direction of care.<br>iv. Outcome of care including complications, prolonged admissions, referrals, and deaths.	vii. Satisfaction with care provided<br>b. Content of care – assess content of care provided including<br>i. Use of the right equipment.<br>ii. Availability and use of the right drugs<br>iii. Counselling and support for care of women and baby during stay and after discharge from facility.
**Mode of assessment:** Observation, inventory taking and testing functional status of equipment	**Mode of assessment:** Desk review of documents, interviews with staff and facility leadership	**Mode of assessment**: Interviews, Desk review of governance records, skills demonstration, or vignettes.	**Mode of assessment**: Observation (passive) of care	**Mode of assessment**: Desk review of client care records, partographs, etc.	**Mode of assessment:** Client and accompanying family interview
