# Supplementary material for: Monitoring healthcare improvement for mothers and newborns: A quantitative review of WHO/UNICEF/UNFPA standards using Every Mother Every Newborn assessment tools
Source: Front Pediatr. 2022 Sep 12;10:959482. doi: 10.3389/fped.2022.959482 (PMC9510702; doi:10.3389/fped.2022.959482)
Supplement: Supplementary file 5 [file Data_Sheet_7.PDF]

Table 1. Performance assessment of UNICEF EMEN tools

	Proportion of quality measures captured*					
	F1. PSFR	F2. MI	F3. SIV	F4. OPCI	F5. CMRR	F6. WEICPC
**Standard 1:** Every woman and newborn receives routine, evidence-based care and management of complications during labour, childbirth and the early postnatal period, according to WHO guidelines.						
Quality statement 1.1a: Women are assessed routinely on admission and during labour and childbirth and are given timely, appropriate care (n=12	*58*	*58*	*83*	*67*	*83*	*33*
Quality statement 1.1b: Newborns receive routine care immediately after birth (n=12)	*75*	*67*	*100*	*83*	*42*	*42*
Quality statement 1.1c: Mothers and newborns receive routine postnatal care (n=19)	*74*	*89*	*89*	*95*	*84*	*79*
Quality statement 1.2: Women with pre-eclampsia or eclampsia promptly receive appropriate interventions, according to WHO guidelines (n=9)	*100*	*33*	*67*	*56*	*100*	*56*
Quality statement 1.3: Women with postpartum haemorrhage promptly receive appropriate interventions, according to WHO guidelines (n=11)	*73*	*45*	*91*	*27*	*100*	*55*
Quality statement 1.4: Women with delay in labour or whose labour is obstructed receive appropriate interventions, according to WHO guidelines (n=16)	*56*	*38*	*81*	*88*	*100*	*44*
Quality statement 1.5: Newborns who are not breathing spontaneously receive appropriate stimulation and resuscitation with a bag-and-mask within 1 min of birth, according to WHO guidelines (n=9)	*100*	*100*	*89*	*89*	*100*	*0*
Quality statement 1.6a: Women in preterm labour receive appropriate interventions for both themselves and their babies, according to WHO guidelines (n=10)	*80*	*30*	*60*	*60*	*90*	*30*
Quality statement 1.6b: Preterm and small babies receive appropriate care, according to WHO guidelines (n=11)	*91*	*100*	*100*	*55*	*91*	*18*
Quality statement 1.7a: Women with or at risk for infection during labour, childbirth or the early postnatal period promptly receive appropriate interventions, according to WHO guidelines (n=10)	*80*	*50*	*100*	*40*	*100*	*80*
Quality statement 1.7b: Newborns with suspected infection or risk factors for infection are promptly given antibiotic treatment, according to WHO guidelines (n=9)	*78*	*89*	*100*	*11*	*100*	*0*
Quality statement 1.8: All women and newborns receive care according to standard precautions for preventing hospital-acquired infections (n=14)	*100*	*86*	*93*	*57*	*36*	*14*
Quality statement 1.9: No woman or newborn is subjected to unnecessary or harmful practices during labour, childbirth and the early postnatal period (n=12)	*50*	*58*	*58*	*50*	*50*	*58*
**Standard 2:** The health information system enables use of data to ensure early, appropriate action to improve the care of every woman and newborn.						
Quality statement 2.1: Every woman and newborn has a complete, accurate, standardized medical record during labour, childbirth and the early postnatal period (n=6)	*67*	*100*	*67*	*33*	*67*	*33*
Quality statement 2.2: Every health facility has a mechanism for data collection, analysis and feedback as part of its activities for monitoring and improving performance around the time of childbirth (n=11)	*100*	*100*	*36*	*18*	*91*	*18*
**Standard 3:** Every woman and newborn with condition(s) that cannot be dealt with effectively with the available resources is appropriately referred.						
Quality statement 3.1: Every woman and newborn is appropriately assessed on admission, during labour and in the early postnatal period to determine whether referral is required, and the decision to refer is made without delay (n=7)	*57*	*57*	*57*	*57*	*100*	*57*
Quality statement 3.2: For every woman and newborn who requires referral, the referral follows a pre-established plan that can be implemented without delay at any time (n=10)	*90*	*100*	*90*	*60*	*60*	*50*
Quality statement 3.3: For every woman and newborn referred within or between health facilities, there is appropriate information exchange and feedback to relevant health care staff (n=6)	*67*	*67*	*67*	*50*	*83*	*50*
**Standard 4:** Communication with women and their families is effective and responds to their needs and preferences.						

Quality statement 4.1: All women and their families receive information about the care and have effective interactions with staff (n=11)	*45*	*82*	*91*	*100*	*36*	*100*
Quality statement 4.2: All women and their families experience coordinated care, with clear, accurate information exchange between relevant health and social care professionals (n=8)	63	75	63	38	50	63
**Standard 5:** Women and newborns receive care with respect and can maintain their dignity.						
Quality statement 5.1: All women and newborns have privacy around the time of labour and childbirth, and their confidentiality is respected (n=8	*75*	*100*	*100*	*75*	*25*	*100*
Quality statement5.2: No woman or newborn is subjected to mistreatment, such as physical, sexual or verbal abuse, discrimination, neglect, detainment, extortion or denial of services (n=16)	38	100	100	56	0	88
Quality statement 5.3: All women can make informed choices about the services they receive, and the reasons for interventions or outcomes are clearly explained (n=10)	30	60	80	80	20	100
**Standard 6**: Every woman and her family are provided with emotional support that is sensitive to their needs and strengthens the woman's capability.						
Quality statement 6.1: Every woman is offered the option to experience labour and childbirth with the companion of her choice (n=7)	*14*	*86*	*43*	*100*	*0*	*86*
Quality statement 6.2: Every woman receives support to strengthen her capability during childbirth (n=12)	*17*	*42*	*42*	*33*	*17*	*58*
**Standard 7:** For every woman and newborn, competent, motivated staff are consistently available to provide routine care and manage complications.						
Quality statement 7.1: Every woman and child has access at all times to at least one skilled birth attendant and to support staff for routine care and management of complications (n=11)	*18*	*91*	*45*	*27*	*36*	*55*
Quality statement 7.2: The skilled birth attendants and support staff have appropriate competence and skills mix to meet the requirements of labour, childbirth and the early postnatal period (n=18)	*22*	*94*	*50*	*11*	*11*	*11*
Quality statement 7.3: Every health facility has managerial and clinical leadership that is collectively responsible for developing and implementing appropriate policies and fosters an environment that supports facility staff in continuous quality improvement (n=14)	*71*	*100*	*57*	*14*	*7*	*14*
**Standard 8:** The health facility has an appropriate physical environment, with adequate water, sanitation and energy supplies, medicines, supplies and equipment for routine maternal and newborn care and management of complications.						
Quality statement 8.1: Water, energy, sanitation, hand hygiene and waste disposal facilities are functioning, reliable, safe and sufficient to meet the needs of staff, women and their families (n=14)	*64*	*64*	*50*	*50*	*7*	*43*
Quality statement 8.2: Areas for labour, childbirth and postnatal care are designed, organized and maintained so that every woman and newborn can be cared for according to their needs in private, to facilitate the continuity of care (n=10)	*100*	*30*	*60*	*60*	*40*	*70*
Quality statement 8.3: Adequate stocks of medicines, supplies and equipment are available for routine care and management of complications (n=18)	*100*	*56*	*83*	*56*	*61*	*72*

Green boxes indicate complete fulfilment of all measures included in the quality statement. Yellow boxes indicate partial (<100%) fulfilment of the measures under the quality statement. Red boxes indicate the tool does not include any measures in that quality statement.

F1. PSFR= Physical, structural, and functional readiness. F2.MI=Management Interviews. F3.SIV=Staff Interviews with Vignettes. F4. OPCI=Observation of Provider-Care Interactions. F5. CMRR=Client Medical Record Review. F6. WEICPC=Women Exit Interviews and Companion Perspectives of Care.
